# CT-Based Intratumoral and Peritumoral Radiomics Nomograms for the Preoperative Prediction of Spread Through Air Spaces in Clinical Stage IA Non-small Cell Lung Cancer

**DOI:** 10.1007/s10278-023-00939-1

**Published:** 2024-01-10

**Authors:** Yun Wang, Deng Lyu, Lei Hu, Junhong Wu, Shaofeng Duan, Taohu Zhou, Wenting Tu, Yi Xiao, Li Fan, Shiyuan Liu

**Affiliations:** 1 Department of Radiology, Second Affiliated Hospital of Navy Medical University, Shanghai, 200003 China; 2 Department of Radiology Medicine, The People’s Hospital of Chizhou, Chizhou, Anhui 247100 China; 3 Department of Radiology Medicine, The People’s Hospital of Guigang, Guigang, Guangxi Zhuang Autonomous Region, 537100 China; 4 GE Healthcare, Precision Health Institution, Shanghai, China

**Keywords:** Spread through air spaces, Nomogram, Radiomics, Prediction, Non-small cell lung cancer

## Abstract

The study aims to investigate the value of intratumoral and peritumoral radiomics and clinical-radiological features for predicting spread through air spaces (STAS) in patients with clinical stage IA non-small cell lung cancer (NSCLC). A total of 336 NSCLC patients from our hospital were randomly divided into the training cohort (*n* = 236) and the internal validation cohort (*n* = 100) at a ratio of 7:3, and 69 patients from the other two external hospitals were collected as the external validation cohort. Univariate and multivariate analyses were used to select clinical-radiological features and construct a clinical model. The GTV, PTV5, PTV10, PTV15, PTV20, GPTV5, GPTV10, GPTV15, and GPTV20 models were constructed based on intratumoral and peritumoral (5 mm, 10 mm, 15 mm, 20 mm) radiomics features. Additionally, the radscore of the optimal radiomics model and clinical-radiological predictors were used to construct a combined model and plot a nomogram. Lastly, the ROC curve and AUC value were used to evaluate the diagnostic performance of the model. Tumor density type (OR = 6.738) and distal ribbon sign (OR = 5.141) were independent risk factors for the occurrence of STAS. The GPTV10 model outperformed the other radiomics models, and its AUC values were 0.887, 0.876, and 0.868 in the three cohorts. The AUC values of the combined model constructed based on GPTV10 radscore and clinical-radiological predictors were 0.901, 0.875, and 0.878. DeLong test results revealed that the combined model was superior to the clinical model in the three cohorts. The nomogram based on GPTV10 radscore and clinical-radiological features exhibited high predictive efficiency for STAS status in NSCLC.

**Supplementary Information:**

The online version contains supplementary material available at 10.1007/s10278-023-00939-1.

## Introduction

In 2015, the World Health Organization (WHO) formally defined STAS (spread through air spaces), that is, tumor cells appear in the lung tissue surrounding the primary tumor in the form of micropapillary cell clusters, solid cancer nests, or single tumor cells, while STAS was identified as the fourth type of invasion mode of lung adenocarcinoma [1]. Regarding STAS-positive patients with clinical or pathological stage IA lung cancer, lobectomy can achieve a superior clinical prognosis compared to sublobectomy and lower the risk of postoperative tumor recurrence and metastasis [2–4]. At present, the gold standard for the diagnosis of STAS is postoperative histopathological examination; consequently, it is challenging to develop appropriate preoperative surgical strategies. Moreover, preoperative puncture and intraoperative frozen pathological examination have drawbacks such as low sensitivity, limited tissue samples, and short diagnosis time [5, 6].

Therefore, scholars attempted to explore the STAS status of lung cancer patients based on preoperative CT images, and the results demonstrated that some radiological features of lung cancer were correlated with STAS status, such as tumor size, solid component size, ratio of solid component size to total tumor size (consolidation-to-tumor ratio, CTR), air bronchogram sign, vacuole sign, spiculation sign, and lobulation sign [7–12]. Additionally, Kim et al. [11] found that STAS was absent in pure ground-glass nodule (pGGN). Based on different inclusion criteria and different multiple regression models, the prediction performance of the model varied. In general, the area under curve (AUC) values of the model ranged from 0.726 to 0.803.

As a characterization algorithm, radiomics offers a non-invasive method to characterize the biological behavior of lesions through high-throughput extraction and analysis of a large number of quantitative image features [13]. Indeed, earlier studies have showcased that intratumoral and peritumoral radiomics techniques have tremendous potential in predicting lymphovascular invasion, lymph node metastasis, and clinical prognosis of lung cancer [14–16]. Both Zhuo et al. [17] and Liao et al. [18] constructed models based on intratumoral and peritumoral radiomics features of clinical T1 stage lung adenocarcinoma including pGGN, but the peritumoral volume of interest (VOI) acquisition methods were different in the two studies, and the conclusions were also varied. In Zhuo’s study [17], a spherical shape was fitted based on the tumor center point, and the spherical peritumoral scope was obtained by extending uniformly to the periphery by 5 mm, 10 mm, and 15 mm, and the results showed that the peritumoral radiomics models were not well fitted. Liao’s study obtained four types of peritumoral segmentation volume (PTV) by extending the range of 5 mm, 10 mm, 15 mm and 20 mm to the periphery along the segmented tumor edge, and the results showed that the gross radiomic signature (GRS) model which combined tumor radiomic signature (TRS) and peritumoral region of 15 mm radiomic signature (PRS-15 mm) achieved the highest values of AUC [18]. Although, the two studies constructed combined models based on the best radiomics signatures, CT morphological features, and clinical information, all of which have achieved good discriminative accuracy based on internal cohort with AUC values of 0.99 and 0.869 in the internal validation cohort, respectively. However, the transportability and generalizability of the models’ predictive efficacy have not been verified through external cohort. Furthermore, previous studies described above only included histologic adenocarcinoma tumors, but STAS was reportedly associated with poor prognosis of other types of lung cancer, such as lung squamous cell carcinoma, lung pleomorphic carcinoma, and lung neuroendocrine tumors [19–21].

In this study, we focused on clinical stage IA NSCLC and excluded pGGN which was clearly STAS-negative. We constructed the radiomics model based on the independent segmented VOI of gross tumor volume (GTV), four types of peritumoral volume (PTV) (5 mm, 10 mm, 15 mm, and 20 mm around the tumor), and their corresponding four types of gross peritumoral and tumor volume (GPTV). We aimed to explore its value in predicting STAS status of clinical stage IA NSCLC and to explore whether it can further improve the diagnostic efficiency combined with relevant radiological features and valuable clinical information in both the internal and external cohorts.

## Materials and Methods

### Patients

The data of NSCLC patients who underwent chest CT examinations and postoperative pathological assessment of STAS status in our hospital and the other two hospitals from September 2019 to September 2022 were retrospectively analyzed. We collected 290 lung cancers presenting as pGGNs, none of which was positive for STAS. As reported in references [11], we excluded pGGNs.

**Fig. 1:**
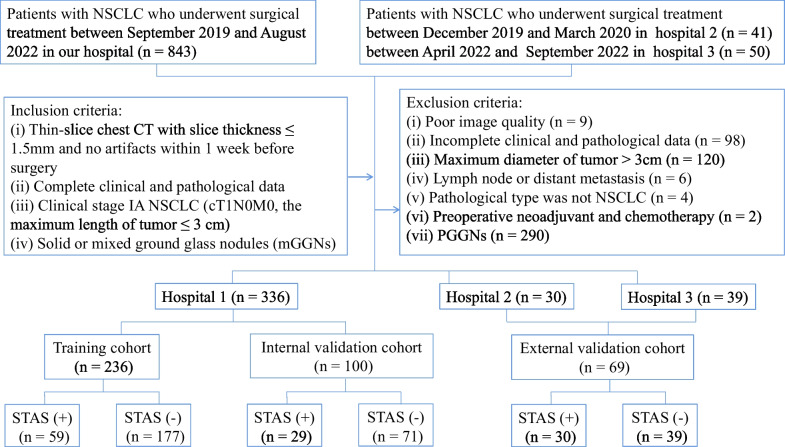
The flow chart for patient selection

The inclusion criteria were as follows: (i) thin-slice chest CT with slice thickness ≤ 1.5 mm and no artifacts within 1 week before surgery; (ii) complete clinical and pathological data; (iii) clinical stage IA NSCLC (cT1N0M0, the maximum tumor diameter ≤ 3 cm); (iv) solid or mixed ground glass nodules (mGGNs). The exclusion criteria were as follows: (i) poor image quality; (ii) incomplete clinical and pathological data; (iii) maximum diameter of tumor > 3 cm; (iv) tumors with lymph node or distant metastasis; (v) the pathological type was not NSCLC; (vi) preoperative neoadjuvant and chemotherapy; (vii) pGGNs. A total of 336 patients were included from our hospital (hospital 1) and randomly divided into the training cohort (*n* = 236) and the internal verification cohort (*n* = 100) at a ratio of 7:3. Furthermore, 69 cases from the other two hospitals were used as the external validation cohort, including 30 patients in hospital 2 and 39 patients in hospital 3. The training cohort was used to train the designed group model, and the validation cohort was used to evaluate the accuracy of the model. The detailed patient inclusion procedure is shown in Fig. 1. The training cohort was used to develop the prediction model, the internal validation cohort was used to test the reproducibility of the model development process, and the external validation cohort was used to evaluate the transportability and generalizability of the model in data from different hospitals.

If multiple lesions in the same patient were surgically removed and met the inclusion criteria, we referred to Dercle et al. [22] to select a representative lesion with the largest tumor size for analysis. Therefore, independent and different patient data were randomly assigned to the training cohort and the internal validation cohort.

This study was approved by the ethics committee of our hospital (hospital 1, decision number: CZ-20220712–03), hospital 2 (decision number: GYLLPJ-20220714–27), and hospital 3 (decision number: CYLLPJ-20220719–08). Due to the retrospective nature of this study, informed consent was waived.

### Equipment and Parameters

Patients in our hospital (hospital 1) underwent preoperative chest CT examinations with four types of CT machines from three vendors, including the Toshiba Aquilion16 row, GE Light Speed VCT64 row, Philips Ingenuity 64 row, and Brilliance iCT 128 row CT machines. In the external cohort, patients from hospital 2 were assessed with American Light Speed 16, Light Speed VCT64 row, and Dutch Philips iCT 256-row CT machines. Hospital 3 utilized the SOMATOM Definition Flash and SOMATOM Drive 64-row CT machines from Germany. The patients were instructed to lie in the supine position during the scan, which covered the entire lung field. The parameters were set as follows: tube voltage was set to 120 kVp, with a tube current of 150–250 mAs or automatic tube current regulation. The scanning slice thickness and slice increment were 5 mm, while the reconstruction slice thickness and slice increment were 1 mm in hospital 1 and hospital 2 and 1.3 mm in hospital 3. The lung algorithm or standard algorithm reconstruction was selected, and non-contrast enhanced images were used for analysis.

### Clinicopathological and Radiological Data

Patient data were collected, including sex, age, clinical symptoms, smoking status, family history of lung cancer, history of malignancy, history of multiple primary lung cancer, CEA levels, surgical type, and pathological type. The DICOM images of patients were imported into software (RadiAnt DICOM Viewer 4.2.1, Medixant, Poland) and analyzed by two independent radiologists with 2 years and 10 years of experience who were blinded to the pathological information. The lung window (width 1500 HU, level − 500 HU), mediastinal window (width 300 HU, level 50 HU), multiplanar reformation (MPR), and maximal intensity projection (MIP) were used to analyze the lesion. For quantitative measures, the average measurements of two independent radiologists were used as the final data. For qualitative indicators, disagreements were discussed until a consensus was reached.

First, the longest diameter of the whole tumor and the consolidation part were measured at the lung window on the MPR images, and the CTR was calculated [7]. Clinical T staging was performed according to the maximum diameter of the solid components of the tumor [23].

Second, we assessed the following qualitative radiological features: tumor location, density type (solid, mGGN), shape (round, irregular), tumor-lung interface (well-defined, ill-defined), margin (lobulation, spiculation), internal characteristics (vacuole sign, cavity/cystic airspace), and external characteristics (vascular convergence, air bronchogram, pleural tags, pleural indentation, ill-defined peripheral opacity, satellite lesions, distal ribbon sign, combined with emphysema).

The definitions of radiological features are described in Supplementary Table S1, and graphical figures of radiological features are shown in Supplementary Fig. S1-S7. Most of the definitions of these features of pulmonary nodules have been previously reported [11, 24–27]

### Image Processing and Model Construction

Standardized image resampling and grayscale discretization were performed on the CT images. ITK-SNAP 3.8.0 software (www.itksnap.org) was used to outline the total volume of the tumor slice by slice along the tumor boundaries, and the GTV was determined, which was used as VOI. GTV was defined as the whole tumor area that was identified within the visible tumor boundary. During segmentation, blood vessels, bronchi, surrounding pleura, and atelectatic lung tissue were avoided as much as feasible. Differences in opinion were resolved by discussion and reaching a consensus. Thirty lesions were randomly selected, and two radiologists with 2 years and 10 years of experience who were blinded to the pathological information independently segmented the tumor to evaluate inter-observer repeatability. One month later, the radiologist with 2 years of working experience performed secondary segmentation on thirty lesions to evaluate intra-observer repeatability. The remaining lesions were segmented by a radiologist with 2 years of working experience. About the definition of peritumoral extent, previous study quantified the histopathologically proven distance between tumor surface and farthest STAS from the tumor edge was 17 mm [28]. Based on the above research, in order to cover all potential STAS, the peripheral extension distance of lung cancer in this study was increased to 20 mm, and four different gradients were set in 5 mm units to make an exploratory study. Python 3.1.1 (https://www.python.org) was used to write the expansion algorithm program to capture the range of 5 mm, 10 mm, 15 mm, and 20 mm peritumoral areas based on the segmented GTV to get the VOIs of GPTV (labeled as GPTV5, GPTV10, GPTV15, and GPTV20, respectively), and pixel filtering was performed on peritumoral non-lung tissues (blood vessels, chest wall, ribs, neck, mediastinum, abdominal cavity) according to the pixel value threshold. Then, the intratumoral mask was subtracted from the GPTV masks to obtain peritumoral areas from the tumor surface, which was the VOIs of PTV (labeled as PTV5, PTV10, PTV15, and PTV20, respectively).

The PyRadiomics open-source software (version 3.0.1, https://pyradiomics.readthedocs.io/en/latest/changes.html) was used to extract radiomics data from images, including 14 morphological features, 18 first-order statistical features, and 68 texture features (22 Gy co-occurrence matrix GLCM, 14 Gy dependence matrix GLDM, 16 Gy size area matrix GLSZM, and 16 Gy run matrix GLRLM). A total of 100 original features were obtained (Supplementary Table S2). In order to acquire high throughput features, the image voxels were transformed by non-linear intensity (square, square root, logarithm, and exponent). Gaussian Laplacian (LoG) conversion was performed with sigma values of 1 mm, 2 mm, 3 mm, 4 mm, and 5 mm, and eight wavelet transforms (LLL, LLH, LHL, LHH, HLL, HLH, HHL, and HHH) were carried out for first-order statistical features and texture features, yielding in a total of 1218 radiomics features (Supplementary Table S3). The definition of each radiomics feature is provided in the Supplementary Table S4. Considering that the CT images of the cases included in this study were collected from multiple hospitals and CT protocols, the intensities of all radiomics features were normalized by the ComBat compensation method (Combat Tool is available here: https://forlhac.shinyapps.io/Shiny_ComBat) and z-score (*z* = *x* − *μ*/*σ*) transformation [29–31].

Intraclass correlation coefficient (ICC) was used to evaluate intra-observer and inter-observer consistency between the segmented intratumoral and peritumoral radiomics features; the “psych” package in R language was used to test the consistency of the radiomics features. Firstly, in order to mitigate overfitting, the maximal redundancy minimal relevance (mRMR) algorithm and the least absolute shrinkage and selection operator (LASSO) logistic regression method were applied to features with a good consistency (ICC > 0.80) in the training cohort to limit the dimension of the features [32]. Secondly, tenfold cross-validation was used to select the optimal regularization parameter *λ* value. Under the optimal *λ* value, features whose coefficients were not equal to 0 were used as the features to construct the radiomics model. Finally, radscore was calculated based on the linear model by selecting the optimal radiomics features, and the Wilcoxon test was used to compare differences between the STAS-positive group and the STAS-negative group. Overall, nine radiomics models were constructed, and their diagnostic efficiency was evaluated. The model with the highest AUC value in the external validation cohort was considered the best radiomics model.

In order to avoid missing meaningful variables, variables with *p* < 0.1 in univariate analysis were involved in multivariate analysis of logistic regression, and backward step-wise selection was applied by using the likelihood ratio test with Akaike’s information criterion (AIC) as the stopping rule to select the best combination of variables to build the clinical prediction model in the training cohort [33].

**Fig. 2:**
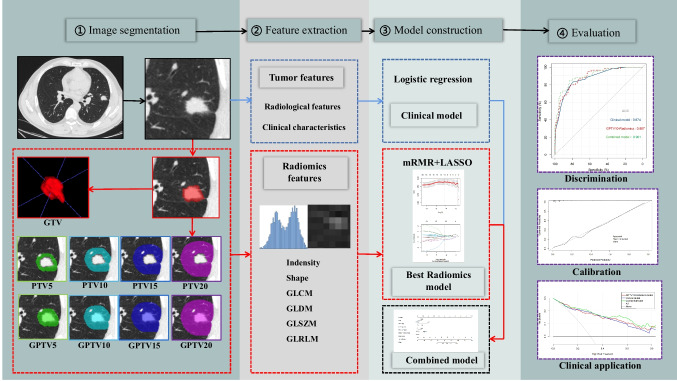
Overall design flow chart of this study

Following this, the radscore of the best radiomics model and clinical predictors were utilized to construct a combined model and design a nomogram, and its predictive efficacy was evaluated centrally in internal and external validation, as illustrated in Fig. 2.

### Pathological Diagnosis

Pathologic diagnosis of each patient included in our study was established by two pathologists, respectively, a junior pathologist and a senior pathologist with more than 10 years of work experience, according to the 2015 WHO definition of STAS [1]. The classification of lung cancer was based on the WHO classification of lung cancer (2015 edition) [1], and the clinical and pathological staging was based on the TNM staging standard of lung cancer (8th edition) [23]. It is important to note that the pathologic diagnoses were determined as part of routine clinical practice, and the specimens were not reviewed specifically for this study.

### Statistical Analysis

SPSS 20.0 software and R statistical software (R version 4.2.2) were used for statistical analysis. Pearson’s chi-squared test, Yate’s correction for continuity, or Fisher’s exact test was used for categorical variables analysis. Regarding the clinical-radiological features selected by univariate and multivariate logistic regression analysis, *P* < 0.05 was considered statistically significant. The ROC curve and AUC value were used to evaluate the diagnostic performance of the model, the DeLong test was used to analyze the difference in AUC value between the models, the Hosmer–Lemeshow test and calibration curve were used to examine the goodness of fit of the model, and decision curve analysis (DCA) was used to analyze the clinical applicability of the model. Inter-observer and intra-observer consistency tests were performed using the “psych” package of R language, the “rms” package of R software was employed for multivariate logistic regression analysis and constructing the nomogram and calibration curves, the “pROC” software package was used for ROC curve analysis, and the “rms” package was applied for internal and external validation. The “dca.R” package was used to analyze the decision curve. The kappa coefficient and ICC were used to evaluate the consistency of qualitative and quantitative parameters among observers, respectively.

## Results

### Clinicopathological Characteristics and Radiological Features

Of the 405 patients with NSCLC, 118 were STAS-positive, and 287 were STAS-negative. Statistically significant differences in sex were observed in the training cohort and the external validation cohort (*P* < 0.05). The difference in the surgical method was statistically significant in the training and internal validation cohorts (*P* < 0.05), whereas the difference in smoking status, carcinoembryonic antigen (CEA) levels, and pathological type was statistically significant exclusively in the training cohort (*P* < 0.05). On the other hand, significant differences were identified in clinical symptoms in the external validation cohort (*P* < 0.05). Concerning radiological features, good consistency was observed in terms of quantitative parameters between the two observers (ICC 0.934–0.935), with strong consistency in qualitative indicators (Kappa 0.852–1.000); the interobserver agreement assessment results of each index are shown in Supplementary Table S5.

### Model Development and Evaluation

The optimal combinations of variables selected by multivariate logistic regression analysis consisted of sex, CEA level, CTR, density type, and distal ribbon sign, among which density type (OR = 6.738, 95% CI 3.107 ~ 15.18, *P* < 0.001) and distal ribbon sign (OR = 5.141, 95% CI 2.272 ~ 12.00, *P* < 0.001) were independent risk factors for STAS (Tables 1 and 2), and there was no multicollinearity (Supplementary Table S6). The AUC values of the clinical model constructed based on the aforementioned variables in the three cohorts were 0.874, 0.822, and 0.810, respectively (Table 3).

**Table 1:** Clinicopathological and radiological characteristics of patients in the training and two validation cohorts

Characteristics	Training cohort (*n* = 236)			Internal validation cohort (*n* = 100)			External validation cohor (*n* = 69)		
	STAS ( −) (*n* = 177)	STAS ( +) (*n* = 59)	*P* value	STAS ( −) (*n* = 71)	STAS ( +) (*n* = 29)	*P* value	STAS ( −) (*n* = 39)	STAS ( +) (*n* = 30)	*P* value
Sex			0.022^a^			0.736^a^			0.003^a^
Male	66 (37.3%)	32 (54.2%)		49 (69.0%)	21 (72.4%)		12 (30.8%)	20 (66.7%)	
Female	111 (62.7%)	27 (45.8%)		22 (31.0%)	8 (27.6%)		27 (69.2%)	10 (33.3%)	
Age (year)			0.108^a^			0.255^a^			0.217^a^
< 65	125 (70.6%)	35 (59.3%)		50 (70.4%)	17 (58.6%)		30 (76.9%)	19 (63.3%)	
≥ 65	52 (29.4%)	24 (40.7%)		21 (29.6%)	12 (41.4%)		9 (23.1%)	11 (36.7%)	
Clinical symptoms			0.153^a^			0.188^a^			0.041^a^
Absent	124 (70.1%)	47 (79.7%)		49 (69.0%)	16 (55.2%)		31 (79.5%)	17 (56.7%)	
Present	53 (29.9%)	12 (20.3%)		22 (31.0%)	13 (44.8%)		8 (20.5%)	13 (43.3%)	
Smoking status			0.015^a^			1.000^b^			0.079^a^
Non-smoker	151 (85.3%)	42 (71.2%)		62 (87.3%)	25 (86.2%)		34 (87.2%)	21 (70.0%)	
Smoker	26 (14.7%)	17 (28.8%)		9 (12.7%)	4 (13.8%)		5 (12.8%)	9 (30.0%)	
Family history of lung cancer			0.441^b^			1.000^b^			N/A
Absent	164 (92.7%)	57 (96.6%)		67 (94.4%)	28 (96.6%)		39 (100.0%)	30 (100.0%)	
Present	13 (7.3%)	2 (3.4%)		4 (5.6%)	1 (3.4%)		0 (0.0%)	0 (0.0%)	
History of malignancy			0.831^a^			1.000^b^			0.528^b^
Absent	151 (85.3%)	51 (86.4%)		60 (84.5%)	25 (86.2%)		35 (89.7%)	29 (96.7%)	
Present	26 (14.7%)	8 (13.6%)		11 (15.5%)	4 (13.8%)		4 (10.3%)	1 (3.3%)	
History of multiple primary lung cancer			0.423^a^			0.321^a^			0.851^b^
Absent	145 (81.9%)	51 (86.4%)		55 (77.5%)	25 (86.2%)		32 (82.1%)	26 (86.7%)	
Present	32 (18.1%)	8 (13.6%)		16 (22.5%)	4 (13.8%)		7 (17.9%)	4 (13.3%)	
CEA (μg/L)			< 0.001^b^			0.685^b^			0.438^b^
< 5	173 (97.7%)	49 (83.1%)		67 (94.4%)	26 (89.7%)		36 (92.3%)	25 (83.3%)	
≥ 5	4 (2.3%)	10 (16.9%)		4 (5.6%)	3 (10.3%)		3 (7.7%)	5 (16.7%)	
Clinical T stage			< 0.001^c^			< 0.001^c^			< 0.001^c^
cT1mi	17 (9.6%)	0 (0.0%)		5 (7.0%)	0 (0.0%)		1 (2.6%)	0 (0.0%)	
cT1a	75 (42.4%)	11 (18.6%)		28 (39.4%)	1 (3.4%)		11 (28.2%)	1 (3.3%)	
cT1b	69 (39.0%)	22 (37.3%)		32 (45.1%)	9 (31.0%)		25 (64.1%)	18 (60.0%)	
cT1c	16 (9.0%)	26 (44.1%)		6 (8.5%)	19 (65.6%)		2 (5.1%)	11 (36.7%)	
CTR (%)			< 0.001^a^			0.008^a^			0.032^c^
< 50	63 (35.6%)	2 (3.4%)		19 (26.8%)	1 (3.4%)		6 (15.4%)	0 (0.0%)	
≥ 50	114 (64.4%)	57 (96.6%)		52 (73.2%)	28 (96.6%)		33 (84.6%)	30 (100.0%)	
Density type			< 0.001^a^			< 0.001^a^			< 0.001^b^
MGGN	154 (87.0%)	19 (32.2%)		63 (88.7%)	11 (37.9%)		21 (53.8%)	3 (10.0%)	
Solid	23 (13.0%)	40 (67.8%)		8 (11.3%)	18 (62.1%)		18 (46.2%)	27 (90.0%)	
Location			0.174^a^			0.783^a^			0.737^a^
RUL	58 (32.8%)	12 (20.3%)		19 (26.8%)	11 (37.9%)		11 (28.2%)	8 (26.7%)	
RML	16 (9.0%)	5 (8.6%)		7 (9.9%)	3 (10.5%)		4 (10.3%)	2 (6.7%)	
RLL	27 (15.3%)	16 (27.1%)		17 (23.9%)	5 (17.2%)		7 (17.9%)	6 (20.0%)	
LUL	53 (29.9%)	16 (27.1%)		17 (23.9%)	5 (17.2%)		10 (25.7%)	5 (16.6%)	
LLL	23 (13.0%)	10 (16.9%)		11 (15.5%)	5 (17.2%)		7 (17.9%)	9 (30.0%)	
Shape			0.307^a^			1.000^b^			0.704^a^
Irregular	26 (14.7%)	12 (20.3%)		12 (16.9%)	5 (17.2%)		20 (51.3%)	14 (46.7%)	
Round/oval	151 (85.3%)	47 (79.7%)		59 (83.1%)	24 (82.8%)		19 (48.7%)	16 (53.3%)	
Tumor-lung interface			0.089^b^			0.026^b^			0.653^a^
Well-defined	167 (94.4%)	51 (86.4%)		68 (95.8%)	23 (79.3%)		34 (87.2%)	25 (83.3%)	
Ill-defined	10 (5.6%)	8 (13.6%)		3 (4.2%)	6 (20.7%)		5 (12.8%)	5 (16.7%)	
Lobulation			0.152^a^			0.095^a^			0.947^a^
Absent	32 (18.1%)	6 (10.2%)		18 (25.4%)	3 (10.3%)		14 (35.9%)	11 (36.7%)	
Present	145 (81.9%)	53 (89.8%)		53 (74.6%)	26 (89.7%)		25 (64.1%)	19 (63.3%)	
Spiculation			< 0.001^a^			0.001^a^			0.042^a^
Absent	156 (88.1%)	34 (57.6%)		64 (90.1%)	18 (62.1%)		32 (82.1%)	18 (60.0%)	
Present	21 (11.9%)	25 (42.4%)		7 (9.9%)	11 (37.9%)		7 (17.9%)	12 (40.0%)	
Vacuole			0.287^a^			0.996^a^			0.685^a^
Absent	127 (71.8%)	38 (64.4%)		49 (69.0%)	20 (69.0%)		31 (79.5%)	25 (83.3%)	
Present	50 (28.2%)	21 (35.6%)		22 (31.0%)	9 (31.0%)		8 (20.5%)	5 (16.7%)	
Cavity/cystic airspace			0.526^a^			0.884^b^			0.501^c^
Absent	161 (91.0%)	52 (88.1%)		66 (93.0%)	26 (89.7%)		37 (94.9%)	30 (100.0%)	
Present	16 (9.0%)	7 (11.9%)		5 (7.0%)	3 (10.3%)		2 (5.1%)	0 (0.0%)	
Bronchial change			0.041^a^			0.359^a^			0.704^a^
Absent	84 (47.5%)	19 (32.2%)		29 (40.8%)	9 (31.0%)		19 (48.7%)	16 (53.3%)	
Present	93 (52.5%)	40 (67.8%)		42 (59.2%)	20 (69.0%)		20 (51.3%)	14 (46.7%)	
Vascular convergence			0.041^a^			0.356^a^			0.009^b^
Absent	150 (84.7%)	43 (72.9%)		53 (74.6%)	19 (65.5%)		35 (89.7%)	18 (60.0%)	
Present	27 (15.3%)	16 (27.1%)		18 (25.4%)	10 (34.5%)		4 (10.3%)	12 (40.0%)	
Pleural tags			0.001^a^			0.135^a^			0.947^a^
Absent	97 (54.8%)	18 (30.5%)		31 (43.7%)	8 (27.6%)		14 (35.9%)	11 (36.7%)	
Present	80 (45.2%)	41 (69.5%)		40 (56.3%)	21 (72.4%)		25 (64.1%)	19 (63.3%)	
Pleural indentation			< 0.001^a^			0.271^a^			0.983^a^
Absent	115 (65.0%)	23 (39.0%)		38 (53.5%)	12 (41.4%)		17 (43.6%)	13 (43.3%)	
Present	62 (35.0%)	36 (61.0%)		33 (46.5%)	17 (58.6%)		22 (56.4%)	17 (56.7%)	
Halo sign			0.015^b^			0.010^b^			1.000^b^
Absent	175 (98.9%)	54 (91.5%)		70 (98.6%)	24 (82.8%)		37 (94.9%)	29 (96.7%)	
Present	2 (1.1%)	5 (8.5%)		1 (1.4%)	5 (17.2%)		2 (5.1%)	1 (3.3%)	
Satellite lesions			0.878^b^			0.360^b^			0.653^a^
Absent	165 (93.2%)	56 (94.9%)		63 (88.7%)	23 (79.3%)		34 (87.2%)	25 (83.3%)	
Present	12 (6.8%)	3 (5.1%)		8 (11.3%)	6 (20.7%)		5 (12.8%)	5 (16.7%)	
Distal ribbon sign			< 0.001^a^			0.001^a^			0.136^a^
Absent	157 (88.7%)	29 (49.2%)		55 (77.5%)	13 (44.8%)		31 (79.5%)	19 (63.3%)	
Present	20 (11.3%)	30 (50.8%)		16 (22.5%)	16 (55.2%)		8 (20.5%)	11 (36.7%)	
ELLC			1.000^b^			0.702^b^			0.103^b^
Absent	163 (92.1%)	54 (91.5%)		69 (97.2%)	27 (93.1%)		38 (97.4%)	25 (83.3%)	
Present	14 (7.9%)	5 (8.5%)		2 (2.8%)	2 (6.9%)		1 (2.6%)	5 (16.7%)	
ERL			1.000^b^			0.416^b^			0.103^b^
Absent	163 (92.1%)	54 (91.5%)		70 (98.6%)	27 (93.1%)		38 (97.4%)	25 (83.3%)	
Present	14 (7.9%)	5 (8.5%)		1 (1.4%)	2 (6.9%)		1 (2.6%)	5 (16.7%)	
Surgery type			0.004^a^			0.023^b^			0.075^b^
Sublobectomy	80 (45.2%)	14 (23.7%)		25 (35.2%)	3 (10.3%)		12 (30.8%)	3 (10.0%)	
Lobectomy	97 (54.8%)	45 (76.3%)		46 (64.8%)	26 (89.7%)		27 (69.2%)	27 (90.0%)	
Pathological type			0.021^c^			0.069^c^			0.588^c^
MIA	14 (7.9%)	0 (0.0%)		7 (9.9%)	0 (0.0%)		1 (2.6%)	0 (0.0%)	
IA	154 (87.0%)	53 (89.8%)		64 (90.1%)	28 (96.6%)		37 (94.8%)	28 (93.2%)	
IMA	5 (2.8%)	4 (6.8%)		0 (0.0%)	0 (0.0%)		1 (2.6%)	0 (0.0%)	
SCC	3 (1.7%)	0 (0.0%)		0 (0.0%)	0 (0.0%)		0 (0.0%)	0 (0.0%)	
ASC	1 (0.6%)	2 (3.4%)		0 (0.0%)	0 (0.0%)		0 (0.0%)	1 (3.4%)	
NSCLC-NOS	0 (0.0%)	0 (0.0%)		0 (0.0%)	1 (3.4%)		0 (0.0%)	1 (3.4%)	

The *P* value represents the univariate analysis. Data are presented as *n* (%)

*STAS* spread through air spaces, *STAS ( −)* STAS-negative, *STAS (* +*)* STAS-positive, *CEA* carcinoembryonic antigen, *cT1mi* tumor with solid component size smaller than 0.5 cm and whole tumor size smaller than 3.0 cm, *cT1a* tumor with solid component size ranged from 0.6 to 1.0 cm and whole tumor size ranged from 0.6 to 3.0 cm, *cT1b* tumor with solid component size ranged from 1.1 to 2.0 cm and whole tumor size ranged from 1.1 to 3.0 cm, *cT1b* tumor with solid component size ranged from 2.1 to 3.0 cm and whole tumor size ranged from 2.1 to 3.0 cm, *CTR* consolidation-to-tumor ratio, *MGGN* mixed ground glass nodule, *RLL* right lower lobe, *RML* right middle lobe, *RUL* right upper lobe, *LUL* left upper lobe, *LLL* left lower lobe, *ELLC* emphysema in the lobe of lung cancer, *ERL* emphysema in the remaining lobes, *MIA* minimally invasive adenocarcinoma, *IA* invasive adenocarcinoma, *IMA* invasive mucinous adenocarcinoma, *SCC* squamous cell carcinoma, *ASC* adenosquamous carcinoma, *NSCLC-NOS* non-small cell lung cancer, not otherwise specified

^a^Pearson’s chi-square

^b^Yate’s correction for continuity

^c^Fisher’s exact test

**Table 2:** Univariable and multivariable logistic regression analysis of factors in the training cohor

Factors	Univariable logistic regression		Multivariable logistic regression	
	OR (95% CI)	*P* value	OR (95% CI)	*P* value
Sex	1.99 (1.10–3.64)	0.023	2.10 (0.98–4.59)	0.058
Smoking status	2.35 (1.15–4.72)	0.017		
CEA	8.83 (2.82–33.31)	< 0.001	3.52 (0.83–17.32)	0.101
Clinical T stage	3.58 (2.34–5.68)	< 0.001		
CTR	15.75 (4.69–98.11)	< 0.001	4.44 (1.16–29.24)	0.057
Density type	14.10 (7.13–29.03)	< 0.001	6.74 (3.11–15.18)	< 0.001
Tumor-lung interface	2.62 (0.95–6.99)	0.054		
Spiculation	5.46 (2.76–10.99)	< 0.001		
Bronchial change	1.90 (1.03–3.60)	0.042		
Pleural tags	2.76 (1.49–5.28)	0.002		
Pleural indentation	2.90 (1.59–5.39)	0.001		
Vascular convergence	2.07 (1.01–4.16)	0.044		
Halo sign	8.10 (1.69–57.70)	0.014		
Distal ribbon sign	8.12 (4.11–16.46)	< 0.001	5.14 (2.27–12.00)	< 0.001

*OR* odds ratio, *CI* confidence interval, *CEA* carcinoembryonic antigen, *CTR* consolidation-to-tumor ratio

**Table 3:** The predictive efficacy of GTV, PTV, GPTV radiomics model in three cohorts

Model	Cohort	AUC (95% CI)	Cut-off	Accuracy	Sensitivity	Specificity
GTV	Training	0.895 (0.849–0.931)	− 0.624	85.17%	76.27%	88.14%
	Internal validation	0.827 (0.738–0.895)		79.00%	68.97%	83.10%
	External validation	0.814 (0.702–0.897)		75.36%	96.67%	58.97%
PTV5	Training	0.874 (0.825–0.914)	− 0.888	82.63%	76.27%	84.75%
	Internal validation	0.767 (0.672–0.846)		71.00%	72.41%	70.42%
	External validation	0.658 (0.534–0.768)		72.46%	53.33%	87.18%
PTV10	Training	0.813 (0.757–0.861)	− 0.701	81.36%	69.49%	85.31%
	Internal validation	0.774 (0.680–0.852)		75.00%	68.97%	77.46%
	External validation	0.621 (0.497–0.735)		66.67%	46.67%	82.05%
PTV15	Training	0.851 (0.799–0.894)	− 1.080	76.69%	79.66%	75.71%
	Internal validation	0.698 (0.598–0.786)		72.00%	62.07%	76.06%
	External validation	0.553 (0.428–0.673)		63.64%	46.67%	79.49%
PTV20	Training	0.841 (0.788–0.885)	− 0.655	81.36%	71.19%	84.75%
	Internal validation	0.672 (0.571–0.763)		78.00%	44.83%	91.55%
	External validation	0.543 (0.418–0.663)		52.17%	86.67%	25.64%
GPTV5	Training	0.880 (0.832–0.919)	− 1.581	77.54%	91.53%	72.88%
	Internal validation	0.852 (0.767–0.915)		76.00%	82.76%	73.24%
	External validation	0.852 (0.746–0.926)		79.71%	86.67%	74.36%
GPTV10	Training	0.887 (0.839–0.924)	− 1.015	81.36%	79.66%	81.92%
	Internal validation	0.876 (0.795–0.933)		71.00%	96.55%	60.56%
	External validation	0.868 (0.764–0.937)		81.16%	86.67%	76.92%
GPTV15	Training	0.901 (0.855–0.936)	− 1.348	79.66%	88.14%	76.84%
	Internal validation	0.847 (0.762–0.912)		68.00%	96.55%	56.34%
	External validation	0.764 (0.647–0.858)		75.36%	80.00%	71.79%
GPTV20	Training	0.832 (0.778–0.877)	− 1.551	68.64%	89.83%	61.58%
	Internal validation	0.860 (0.776–0.921)		81.00%	89.66%	77.46%
	External validation	0.864 (0.760–0.935)		79.71%	90.00%	71.79%

*AUC* area under the curve, *CI* confidence interval, *GTV* gross tumor volume, *PTV* peritumoral tumor volume, *GPTV* gross peritumoral tumor volume

Among the 1218 radiomics features extracted from each VOI, the proportion of radiomics features with inter-observer and intra-observer ICC greater than 0.8 ranged from 86.0 to 99.8% (Supplementary Table S7). Among the features with ICC > 0.80, the mRMR algorithm was first used to eliminate redundant and irrelevant features, and 30 features were retained in each group. Then, LASSO logistic regression method was used to select the optimized feature subset to establish the final model, and tenfold cross-validation was used to select the values of the optimal hyperparameter *λ*, which were identified to be 0.0006 (GTV), 0.0033 (PTV5), 0.0226 (PTV10), 0.0139 (PTV15), 0.0071 (PTV20), 0.0143 (GPTV5), 0.0135 (GPTV10), 0.0054 (GPTV15), and 0.0955 (GPTV20), respectively. With the optimal *λ* values, 19, 16, 9, 15, 16, 11, 10, 12, and 2 features were selected to construct the 9 radiomics models. The features used for radiomics model construction and their ICC details are shown in Supplementary Table S8. The group with the highest AUC value in the external validation cohort was considered the best radiomics model. The results demonstrated that the GPTV10 radiomics model had the best prediction performance, with AUC values of 0.887, 0.876, and 0.868, in the three cohorts (Table 3). Besides, the DeLong test demonstrated that the GPTV10 model significantly outperformed PTV10 and GPTV20 in the training cohort, PTV (5, 10, 15, 20) in the internal validation cohort, and PTV (5, 10, 15, 20) and GPTV15 in the external validation cohort (*P* < 0.05). Detailed results of DeLong tests between radiomics models are shown in Supplementary Table S9-S11.

**Fig. 3:**
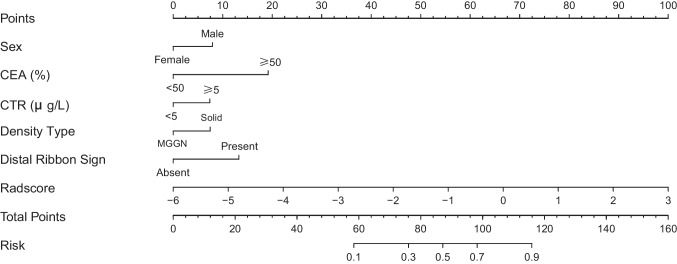
Nomogram for preoperative prediction of STAS status based on intratumoral and peritumoral radiomics and clinical-radiological features in clinical stage IA NSCLC

**Fig. 4:**
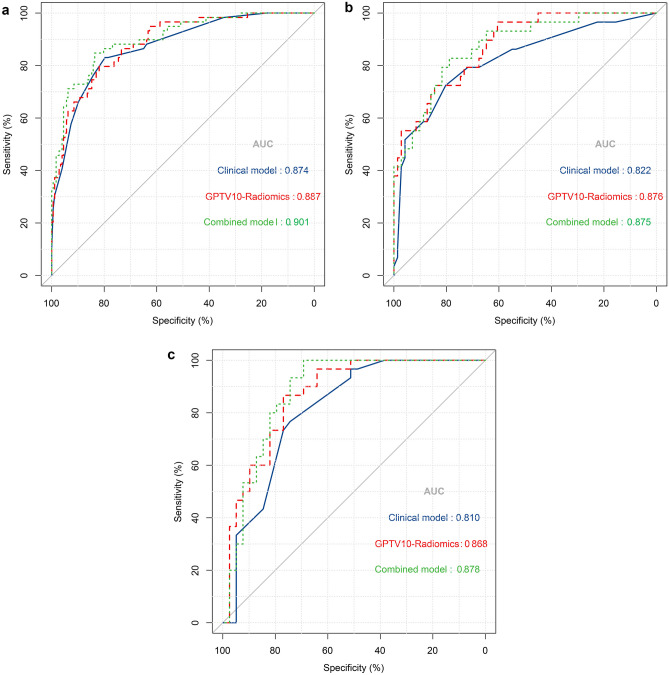
ROC curve analysis of the clinical model, GPTV10 radiomics model, and combined model in three cohorts. **a** The training cohort; **b** the internal validation cohort; **c** the external validation cohort

**Fig. 5:**
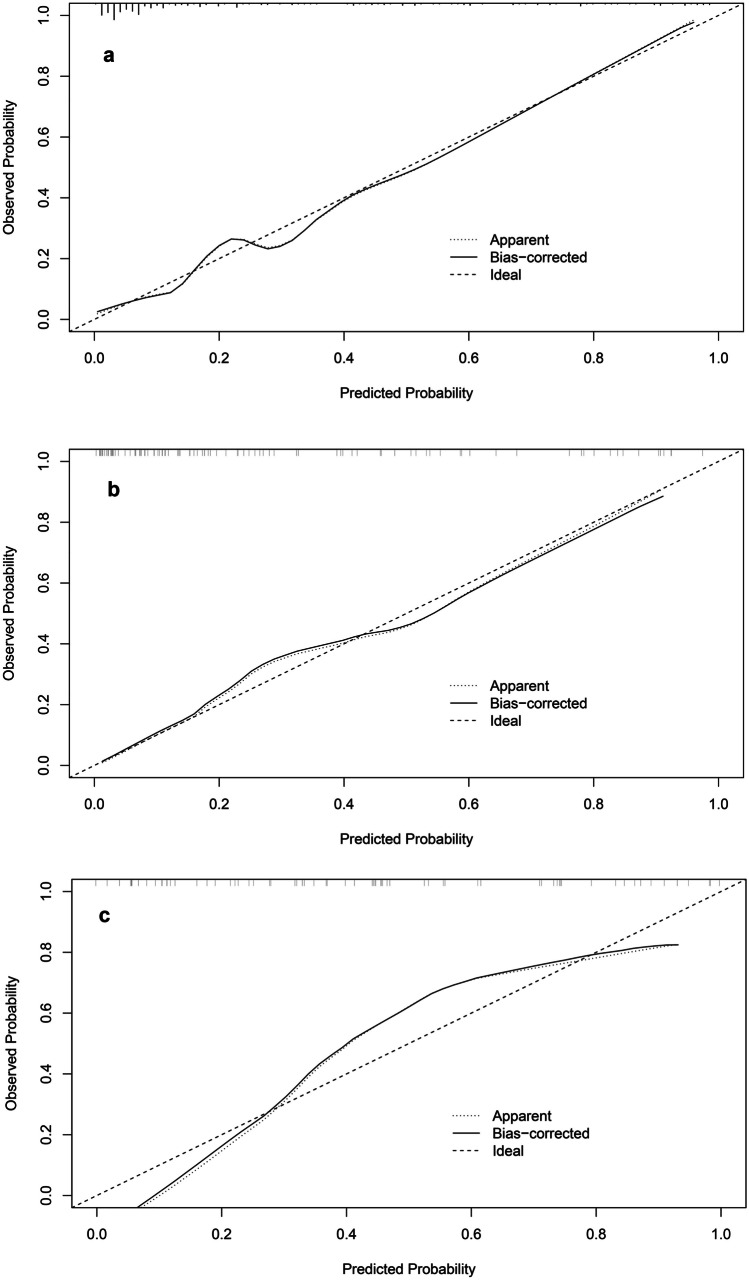
The calibration curves of combined model in the three cohorts. **a** The training cohort; **b** the internal validation cohort; **c** the external validation cohort

**Fig. 6:**
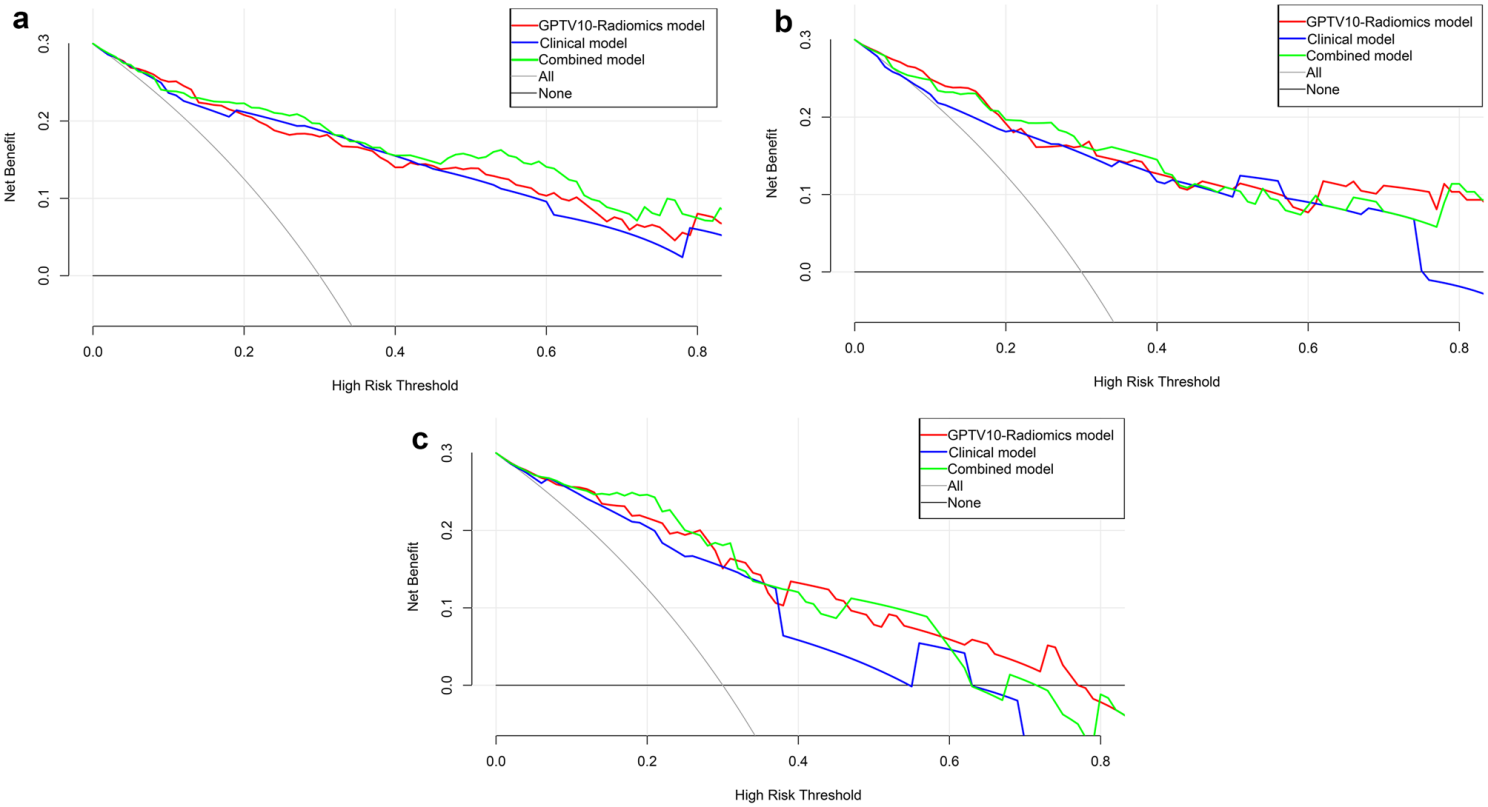
The decision curve shows that the combined model has better clinical application value than the clinical model and GPTV10 radiomics model in the three cohorts. **a** The training cohort; **b** the internal validation cohort; **c** the external validation cohort

Based on GPTV10 radscore and the selected clinical-radiological predictors, a combined model was constructed, and a nomogram was developed, as delineated in Fig. 3. The combined model formula is as follows: Nomoscore = (Intercept) ×  − 1.56 + sex × 0.60 + CEA × 1.46 + CTR × 0.56 + density type × 0.57 + distal ribbon sign × 1.01 + GPTV10 radscore × 0.85, and its AUC values in the three cohorts were 0.901, 0.875, and 0.878 (Table 4). ROC curves of the clinical model, GPTV10 radiomics model, and combined model in the three cohorts are presented in Fig. 4. The DeLong test showed that the combined model was superior to the clinical model in the three cohorts (*Z* = 2.480, 2.068, 2.388, *P* < 0.05), detailed results of DeLong tests between models in three cohorts are shown in Supplementary Table S12. Meanwhile, the Hosmer–Lemeshow test showed that the combined model was well-fitted in all three cohorts (*P* = 0.473, 0.496, 0.246), and the calibration curve portrayed that the predicted probability value of the combined model was in good agreement with the actual situation, as shown in Fig. 5. Lastly, the DCA illustrated that the combined model had superior clinical application value compared to the clinical model or the GPTV10 radiomics model alone, as illustrated in Fig. 6.

**Table 4:** The predictive efficacy of clinical model, GPTV10 radiomics model, and combined model in three cohorts

Model	Cohort	Cut-off	AUC (95% CI)	Accuracy	Sensitivity	Specificity	PPV	NPV
Clinical	Training	0.264	0.874 (0.825–0.914)	80.51%	83.05%	79.66%	57.65%	93.37%
	Internal validation		0.822 (0.733–0.891)	78.00%	72.41%	80.28%	60.00%	87.69%
	External validation		0.810 (0.697–0.894)	75.36%	76.67%	74.36%	70.00%	80.56%
GPTV10	Training cohort	− 1.015	0.887 (0.839–0.924)	81.36%	79.66%	81.92%	59.49%	92.36%
	Internal validation		0.876 (0.795–0.933)	71.00%	96.55%	60.56%	50.00%	97.73%
	External validation		0.868 (0.764–0.937)	81.16%	86.67%	76.92%	74.29%	88.23%
Combined	Training cohort	− 1.226	0.901 (0.856–0.936)	83.90%	84.75%	83.62%	63.29%	94.27%
	Internal validation		0.875 (0.793–0.932)	80.00%	82.76%	78.87%	61.54%	91.80%
	External validation		0.878 (0.777–0.944)	82.61%	100%	69.23%	71.43%	100%

*AUC* area under the curve, *CI* confidence interval, *PPV* positive predictive value, *NPV* negative predictive value, *GPTV* gross peritumoral tumor volume

## Discussion

STAS, as an important invasive mode of lung cancer, affects the postoperative recurrence-free survival (RFS) and overall survival (OS) of patients [2]. Accurate diagnosis of STAS status prior to surgery is conducive to the clinical selection of the optimal surgical method, thereby prolonging survival and enhancing the prognosis of patients [3, 4]. Herein, a combined model was constructed based on GPTV10 radscore and clinical-radiological features to predict STAS status in patients with stage IA non-small cell lung cancer. The results showed that the combined model had high diagnostic efficacy for STAS, with AUC values of 0.901, 0.875, and 0.878, in the three cohorts. Moreover, the accuracy was 83.90%, 80.00%, and 82.61%; the sensitivity was 84.75%, 82.76%, and 100%; and the specificity was 83.62%, 78.87%, and 69.23%. In addition, the created nomogram can transform complex regression equations of the model into a visual graph, which is intuitive and easily interpreted, as well as facilitate the preoperative assessment of STAS status.

In this study, spiculation sign, pleural indentation sign, and vascular convergence sign were more common in the STAS-positive group, which was consistent with previous literature reports [34, 35] and might be related to its pathological mechanism. Spiculation is associated with tumor cell infiltration into adjacent blood and lymphatic vessels, suggesting lung cancer is more aggressive [36], while pleural indentation arises from intratumor reactive fibrous hyperplasia, pulling the adjacent pleura and causing the deviation of the pleura from its original position [37]. Vascular convergence sign is also caused by the reactive fibrous hyperplasia of the tumor, which pulls the adjacent pulmonary vessels, causing them to converge toward the tumor [38]. Notably, a higher degree of tumor infiltration is associated with a higher level of internal reactive fibroplasia and a higher probability of pleural indentation and vascular convergence, indicating a greater risk of STAS. The results of this study revealed that tumor density was an independent risk factor for STAS. Among them, the incidence of STAS in patients with solid nodules on CT images was 63.4% (85/134), while that in patients with mGGNs was 12.1% (33/271), which was consistent with the results of previous studies [11, 39, 40]. Solid components typically represent the more aggressive section of the tumor. It is worthwhile emphasizing that prior studies have pointed out that CTR was positively correlated with STAS while negatively correlated with ground-glass opacity (GGO) positivity. Higher CTR values were associated with more aggressive tumors; the more likely STAS positivity is, the worse the prognosis of patients [40]. In this study, the STAS-positive rate was 36.6% (115/314) in patients with CTR ≥ 50%. Conversely, the STAS-positive rate was merely 3.3% (3/91) in patients with CTR < 50%, signaling a higher risk of STAS in cases with higher solid components on CT images, which was in agreement with the observation of previous research. This study also found that the distal ribbon sign was an independent risk factor for STAS. This might be attributed to tumor cells escaping from the primary lesion, redistributing through the airway, and proliferating along the surrounding alveolar wall, resulting in parenchymal obstruction of the surrounding lung or obstruction of the terminal bronchioles, thereby reducing the gas content in the alveoli. This phenomenon is similar to the ground glass ribbons discovered by Qi et al. [35]. In general, a higher degree of tumor invasion is associated with a higher incidence of STAS, which is reflected by a higher proportion of tumor solid components on CT images and more malignant radiological features. Lobectomy is recommended for this type of early lung cancer.

In the present study, on the premise of accurate tumor segmentation, four peritumoral regions with different gradient ranges were automatically expanded to construct nine radiomics models in order to explore the most efficient radiomics model to predict STAS status, and a multi-center study was conducted to evaluate the generalizability of the model. The results showed that the GPTV10 radiomics model had the highest predictive efficiency. Kadota et al. [28] found that, in 97% (151/155) of the STAS-positive cases, the distance between STAS and the edge of the primary tumor lesion ranged from 0.3 to 10.5 mm, and the peritumoral extension range of 10 mm may accurately cover various high-order features related to the heterogeneity of lung cancer. Based on the VOI of GPTV10, 10 best radiomics features were selected, including the two first-order features, which was correlated with the CT value of the tumor. The higher the CT value, the denser the tumor cells, and the higher the degree of tumor invasion. The eight texture features are Small Area Low Gray Level Emphasis, Gray Level Variance, Zone Entropy, Cluster Shade, Cluster Prominence, Imc2, Large Dependence Low Gray Level Emphasis, and Large dependence High Gray Level Emphasis. Small Area Low Gray Level Emphasis, Gray Level Variance, and Zone Entropy are parameters of GLSZM and principally provide information on the uniform area size of each gray level on the 3D image. Cluster Shade, Cluster Prominence, and Imc2 are parameters of GLCM that chiefly evaluate the spatial relationship between pixels and describe the frequency of appearance of specific pixel combinations in the image. Large Dependence Low Gray Level Emphasis and Large Dependence High Gray Level Emphasis are parameters of GLDM that mainly reflect the grayscale relationship between the central pixel and its neighborhood. More specifically, the larger the values of LDLGLE, GLCM, and GLDM, the more uneven the image texture distribution and the more irregular the gray change, indicating a higher spatial heterogeneity of tumor [16, 41] and reflecting its strong aggressiveness and a greater possibility of STAS. This study also noted that the GPTV radiomics model was superior to the GTV and PTV models; the GTV radiomics model was superior to the PTV model, inferring that the occurrence of STAS was predominantly related to the aggressiveness of the tumor itself and that the peritumoral region also partly reflected the aggressive behavior of the tumor. As a quantitative method, radiomics techniques can be used to quantify internal tumor heterogeneity and differences in the peritumoral microenvironment [42].

In this study, a combined model was constructed based on GPTV10 radscore and clinical-radiological features to predict STAS status. The DeLong test results demonstrated that the predictive value of the combined model in the three cohorts was superior to that of the clinical model. Hosmer–Lemeshow test and calibration curve showed that the combined model fitted well in all three cohorts. DCA validated that the combined model had better clinical application value than the clinical model, which can be ascribed to the excellent predictive efficiency of the GPTV10 radiomics model. The evaluation of traditional CT features of tumors mostly depends on the experience of radiologists, which is subjective to a certain extent, and different conclusions can be obtained from different clinical levels [43]. Radiomics can extract massive high-dimensional features from segmented images, including gray level changes and voxel spatial relationships, and then achieve accurate diagnosis and prognosis assessment of diseases through feature selection and model establishment [44]. It can not only avoid the subjectivity of the observer’s interpretation of the CT morphological features, but also deeply excavate and integrate a large number of digital information in the image which cannot be recognized and distinguished by human eyes [44]. At present, only one study has been conducted on the prediction of lung cancer STAS based on deep learning, Tao et al. [45] constructed a three-dimensional (3D) convolutional neural network (CNN) model based on 203 cases of any stage of NSCLC patients with preoperative enhanced thin-slice CT; the results showed that the 3D CNN model yielded superior performance with AUC values of 0.93 and 0.80 in the training and validation cohorts. With high repeatability and reliability, radiomics and deep learning techniques may become a non-invasive precision diagnostic tool reflecting tumor biological behavior for clinical application in the future [44, 45].

This study has several limitations. Firstly, the sample size of this study was relatively small, but through strict case selection, and excluding pGGN that was clearly STAS negative, the cases were more representative; we will continue to collect more cases and construct prediction models based on deep learning technology to explore whether the prediction performance can be further improved. Secondly, the study was retrospective, resulting in inevitable selection bias; we will prospectively and continuously collect large samples of external center cases and take pathological results as the gold standard to further verify the repeatability of the nomogram constructed in this study. Third, due to the lack of detailed follow-up data, whether the nomogram developed in this study can further accurately predict the prognosis of patients, so as to make prognostic risk stratification, we will conduct this study in the future when more follow-up data are collected.

## Conclusions

In conclusion, the GPTV10 radiomics model performs better than the GTV, all PTV, and the other three GPTV radiomics models in predicting the STAS status of clinical stage IA NSCLC preoperatively. Additionally, the nomogram based on GPTV10 radiomics features and relevant clinical-radiological predictors can further improve predictive efficiency, which will assist in timely providing guidance and aiding in the development of personalized treatment strategies for early lung cancer.

### Supplementary Information

Below is the link to the electronic supplementary material.

Supplementary file1 (DOCX 1221 KB)

## Data Availability

The data that support the findings of this study are available on request from the corresponding author.
