# Supplementary material for: CT-Based Intratumoral and Peritumoral Radiomics Nomograms for the Preoperative Prediction of Spread Through Air Spaces in Clinical Stage IA Non-small Cell Lung Cancer
Source: J Imaging Inform Med. 2024 Jan 10;37(2):520–35. doi: 10.1007/s10278-023-00939-1 (PMC11031508; doi:10.1007/s10278-023-00939-1)
Supplement: Supplementary file 1 — Supplementary file1 (DOCX 1221 KB) [file 10278_2023_939_MOESM1_ESM.docx]

**Supplementary Materials**

**List**

**1.Supplementary Tables**

**2.Supplementary Figures**

**Supplementary Tables**

**Table S1** The definitions of radiological features

| Feature | Definition |
| --- | --- |
| Tumor size | The longest diameter of the whole tumor at the lung window on the MPR image |
| Solid component size | The longest diameter of the solid component of the tumor at the lung window on the MPR image |
|  |  |
| CTR | The proportion of the solid component part (consolidation-to-tumor ratio，CTR) |
| Location | Lobe of tumor originated from |
| Density | mGGN, presence of ground-glass opacity and solid density component; solid, absence of ground-glass opacity, contains solid density component only; |
| Shape | Shape of tumor on the multiplanar reconstructed (MPR) images, including round/oval, irregular shape |
| Tumor-lung interface | Interface of the tumor-lung, including well-defined or ill-defined interface |
| Marginal characteristics |  |
| Lobulation Sign | Petaloid or wavy appearance at the tumor’s margins |
| Spiculation Sign | Short, thin linear strands radiating around the surface of the tumor without reaching the pleural surface |
| Internal characteristics |  |
| Vacuole sign | A small air containing space (≤ 5mm) in the tumor, referring to lung tissue not invaded by the tumor |
| Cavity or cystic airspace | A larger air containing space (>5mm) in the tumor because of intratumoral necrosis or represents spared parenchyma, normal or ectatic bronchi, or focal emphysema |
| External characteristics |  |
| Bronchial change | Air-filled bronchus manifesting as natural, dilated/distorted or cut-off within the lesions, or cut-off at the edge of the lesions. |
| Vascular convergence sign | The convergence of pulmonary vessels around the tumor towards the lesion. |
| Pleural tags sigh | One or multiple high-density linear strands connecting the tumor margin and the pleura. |
| Pleural indentation sigh | The deviation of the pleura from its original position due to tumor traction at the lung window. |
| Halo sigh | Ill-defined peripheral ground-glass opacity or consolidation around the tumor, which should be distinguished from the well-defined ground-glass opacity of a part-solid lesion |
| Satellite lesion sigh | Smaller nodules located within 2 cm of the primary tumor. |
| Distal ribbon sign | A long, thick ribbon strand extending from the distal part of the tumor into the surrounding lung tissue |
| ELLC | Presence of emphysema in the lobe of lung cancer with visual observation |
| ERL | Presence of emphysema in the remaining lobes with visual observation |

**Table S2** A total of 100 Original features

|  | **Shape** | **First order** | **GLCM** | **GLRLM** | **GLSZM** | **GLDM** |
| --- | --- | --- | --- | --- | --- | --- |
| 1 | Elongation | 10Percentile | Autocorrelation | GrayLevelNonUniformity | GrayLevelNonUniformity | DependenceEntropy |
| 2 | Flatness | 90Percentile | JointAverage | GrayLevelNonUniformityNormalized | GrayLevelNonUniformityNormalized | DependenceNonUniformity |
| 3 | LeastAxisLength | Energy | ClusterProminence | GrayLevelVariance | GrayLevelVariance | DependenceNonUniformityNormalized |
| 4 | MajorAxisLength | Entropy | ClusterShade | HighGrayLevelRunEmphasis | HighGrayLevelZoneEmphasis | DependenceVariance |
| 5 | MinorAxisLength | InterquartileRange | ClusterTendency | LongRunEmphasis | LargeAreaEmphasis | GrayLevelNonUniformity |
| 6 | Maximum2DDiameterColumn | Kurtosis | Contrast | LongRunHighGrayLevelEmphasis | LargeAreaHighGrayLevelEmphasis | GrayLevelVariance |
| 7 | Maximum2DDiameterRow | Maximum | Correlation | LongRunLowGrayLevelEmphasis | LargeAreaLowGrayLevelEmphasis | HighGrayLevelEmphasis |
| 8 | Maximum2DDiameterSlice | MeanAbsoluteDeviation | DifferenceAverage | LowGrayLevelRunEmphasis | LowGrayLevelZoneEmphasis | LargeDependenceEmphasis |
| 9 | Maximum3DDiameter | Mean | DifferenceEntropy | RunEntropy | SizeZoneNonUniformity | LargeDependenceHighGrayLevelEmphasis |
| 10 | Sphericity | Median | DifferenceVariance | RunLengthNonUniformity | SizeZoneNonUniformityNormalized | LargeDependenceLowGrayLevelEmphasis |
| 11 | SurfaceArea | Minimum | JointEnergy | RunLengthNonUniformityNormalized | SmallAreaEmphasis | LowGrayLevelEmphasis |
| 12 | SurfaceVolumeRatio | Range | JointEntropy | RunPercentage | SmallAreaHighGrayLevelEmphasis | SmallDependenceEmphasis |
| 13 | VoxelVolume | RobustMeanAbsoluteDeviation | Imc1 | RunVariance | SmallAreaLowGrayLevelEmphasis | SmallDependenceHighGrayLevelEmphasis |
| 14 | MeshVolume | RootMean  Squared | Imc2 | ShortRunEmphasis | ZoneEntropy | SmallDependenceLowGrayLevelEmphasis |
| 15 |  | Skewness | Idm | ShortRunHighGrayLevelEmphasis | ZonePercentage |  |
| 16 |  | TotalEnergy | Idmn | ShortRunLowGrayLevelEmphasis | ZoneVariance |  |
| 17 |  | Uniformity | Id |  |  |  |
| 18 |  | Variance | Idn |  |  |  |
| 19 |  |  | InverseVariance |  |  |  |
| 20 |  |  | MaximumProbability |  |  |  |
| 21 |  |  | SumEntropy |  |  |  |
| 22 |  |  | SumSquares |  |  |  |

**Table S3** 1218 radiomics features

|  | Original | Laplacian of Gaussian filter | Wavelet |
| --- | --- | --- | --- |
| Shape (14) | 14×1 |  |  |
| First-order Statistics (18) | 18×1 | 18×5 | 18×8 |
| Texture (68) | 68×1 | 68×5 | 68×8 |
| Total 1218 radiomics features | | | |

**Table S4** The definitions of radiomics features

| **Shape feature** | **Definition** |
| --- | --- |
| Elongation | Elongation$=\sqrt{\frac{\lambda_{minor}}{\lambda_{major}}}$  Here, $\lambda_{major}$ and $\lambda_{minor}$ are the lengths of the largest and second largest principal component axes. The values range between 1 (where the cross section through the first and second largest principal moments is circle-like (non-elongated)) and 0 (where the object is a single point or 1 dimensional line). |
| Flatness | Flatness$=\sqrt{\frac{\lambda_{least}}{\lambda_{major}}}$  Here, $\lambda_{major}$ and $\lambda_{least}$ are the lengths of the largest and smallest principal component axes. The values range between 1 (non-flat, sphere-like) and 0 (a flat object). |
| Least Axis Length | $\mathrm{LeastAxisLength}=4\sqrt{\lambda_{least}}$  This feature yield the smallest axis length of the ROI-enclosing ellipsoid and is calculated using the largest principal component $\lambda_{least}$. In case of a 2D segmentation, this value will be 0. |
| Major Axis Length | MajorAxisLength$=4\sqrt{\lambda_{major}}$  This feature yield the largest axis length of the ROI-enclosing ellipsoid and is calculated using the largest principal component $\lambda_{major}$. |
| Minor Axis Length | MinorAxisLength$=4\sqrt{\lambda_{minor}}$  This feature yield the second-largest axis length of the ROI-enclosing ellipsoid and is calculated using the largest principal component $\lambda_{minor}$. |
| Maximum 2D Diameter Column | Maximum 2D diameter (Column) is defined as the largest pairwise Euclidean distance between tumor surface voxels in the row-slice (usually the coronal) plane. |
| Maximum 2D Diameter Row | Maximum 2D diameter (Row) is defined as the largest pairwise Euclidean distance between tumor surface voxels in the column-slice (usually the sagittal) plane. |
| Maximum 2D Diameter Slice | Maximum 2D diameter (Slice) is defined as the largest pairwise Euclidean distance between tumor surface voxels in the row-column (generally the axial) plane. |
| Maximum 3D Diameter | Maximum 3D diameter is defined as the largest pairwise Euclidean distance between surface voxels in the ROI.  Also known as Feret Diameter. |
| Sphericity | Sphericity$=\frac{\sqrt[3]{36\pi V^{2}}}{A}$  Sphericity is a measure of the roundness of the shape of the tumor region relative to a sphere. It is a dimensionless measure, independent of scale and orientation.The value range is 0 < sphericity ≤ 1, where a value of 1 indicates a perfect sphere (a sphere has the smallest possible surface area for a given volume, compared to other solids). |
| Surface Area | A$=\sum_{i=1}^{N} \frac{1}{2}\left\vert a_{i}b_{i}{\times a}_{i}c_{i} \right\vert$  N is the number of triangles forming the surface mesh of the volume (ROI) $a_{i}b_{i}$ and$a_{i}c_{i}$ are the edges of the ith triangle formed by points $a_{i}$, $b_{i}$ and $c_{i}$.Surface Area is an approximation of the surface of the ROI in mm2, calculated using a marching cubes algorithm. |
| Surface Volume Ratio | SurfaceVolumeRatio$=\frac{A}{V}$  Here, a lower value indicates a more compact (sphere-like) shape. This feature is not dimension less, and is therefore (partly) dependent on the volume of the ROI. |
| Voxel Volume | $Vvoxel=\sum_{k=1}^{Nv} V_{k}$  The volume of the ROI $\mathrm{Vvoxel}$ is approximated by multiplying the number of voxels in the ROI by the vol ume of a single voxel $V_{k}$. This is a less precise approximation of the volume and is not used in subsequent features. This feature does not make use of the mesh and is not used in calculation of other shape features. |
| Mesh Volume | $V_{i}=\frac{Oa_{i}\times(Ob_{i}\times Oc_{i})}{6}$  $V=\sum_{i=1}^{Nf} V_{i}$  The volume of the ROI $V$ is calculated from the triangle mesh of the ROI. For each face *i* in the mesh, defined by points $a_{i}$,$b_{i}$ and $c_{i}$, the (signed) volume $V_{f}$ of the tetrahedron defined by that face and the origin of the image ($O$) is calculated. The sign of the volume is determined by the sign of the normal, which must be consistently defined as either facing outward or inward of the ROI. Then taking the sum of all V*i*, the total volume of the ROI is obtained. |
| *Nv* represent the number of *voxels* included in the ROI  *Nf* represent the number of faces (triangles) defining the Mesh  *V* the volume of the mesh in mm^3^  *A* the surface area of the mesh in mm^2^ | |

| **First order feature** | **Definition** |
| --- | --- |
| 10Percentile | The 10^th^ percentile of **X** |
| 90Percentile | The 90^th^ percentile of **X** |
| Energy | $\mathrm{Energy}=\sum_{i=1}^{N} ({\boldsymbol{X}\left( i \right)+c)}^{2}$  Here, $c$ is optional value, dened by “voxelArrayShift”, which shifts the inten sities to prevent negative values in **X**. This ensures that voxels with the lowest gray values contribute the least to Energy, instead of voxels with gray level intensity closest to 0. Energy is a measure of the magnitude of voxel values in an image. A larger values implies a greater sum of the squares of these values. |
| Entropy | $\mathrm{En}\mathrm{tropy}=-\sum_{i=1}^{N_{i}} p\left( i \right){log}_{2}(p\left( i \right)+\in)$  Here, $\in$ is an arbitrarily small positive number (≈ 2:2 × 10^-16^).Entropy species the uncertainty/randomness in the image values. It measures the average amount of information required to encode the image values |
| Interquartile Range | InterquartileRange$=P_{75}-P_{25}$  Here $\mathbf{P}_{\mathbf{25}}$ and $\mathbf{P}_{\mathbf{75}}$ are the 25^th^ and 75^th^ percentile of the image array, respectively. |
| Kurtosis | $Kurtosis=\frac{\mu_{4}}{\sigma^{4}}=\frac{\frac{1}{N}\sum_{I=1}^{N} {(X\left( i \right)-\bar{X})}^{4}}{{(\frac{1}{N}\sum_{I=1}^{N} {(X\left( i \right)-\bar{X})}^{2})}^{2}}$  Where µ4 is the 4^th^ central moment.Kurtosis is a measure of the “peakedness” of the distribution of values in the image ROI. A higher kurtosis implies that the mass of the distribution is concentrated towards the tail(s) rather than towards the mean. A lower kurtosis implies the reverse: that the mass of the distribution is concentrated towards a spike near the Mean value. |
| Maximum | Maximum = max(**X**)  The maximum gray level intensity within the ROI. |
| Mean Absolute Deviation | $\mathrm{MAD}=\frac{1}{N}\sum_{i=1}^{N} \left\vert X\left( i \right)-\bar{X} \right\vert$  Mean Absolute Deviation is the mean distance of all intensity values from the Mean Value of the image array. |
| Mean | $M\mathrm{ean}=\frac{1}{N}\sum_{i=1}^{N} X\left( i \right)$  The average gray level intensity within the ROI. |
| Median | The median gray level intensity within the ROI. |
| Minimum | Minimum = min(**X**) |
| Range | Range = max(**X**) - min(**X**)  The range of gray values in the ROI. |
| Robust Mean Absolute Deviation | $rMAD=\frac{1}{N10-90}\sum_{i=1}^{N10-90} (X10-90\left( i \right)-\bar{X}10-90)$  Robust Mean Absolute Deviation is the mean distance of all intensity values from the Mean Value calculated on the subset of image array with gray levels in between, or equal to the 10th and 90thpercentile. |
| Root Mean Squared | $\mathrm{RMS}=\sqrt{\frac{1}{N}\sum_{i=1}^{N} ({X\left( i \right)+c)}^{2}}$  Here, $c$ is optional value, defined by “voxelArrayShift”, which shifts the intensities to prevent negative values in **X**. This ensures that voxels with the lowest gray values contribute the least to RMS, instead of voxels with gray level intensity closest to 0.RMS is the square-root of the mean of all the squared intensity values. It is another measure of the magnitude of the image values. This feature is volume-confounding, a larger value of $c$ increases the effect of volume-confounding. |
| Skewness | $Skewness=\frac{\mu_{3}}{\sigma^{3}}=\frac{\frac{1}{N}\sum_{I=1}^{N} {(X\left( i \right)-\bar{X})}^{3}}{{(\sqrt{\frac{1}{N}\sum_{I=1}^{N} {(X\left( i \right)-\bar{X})}^{2}})}^{3}}$  Where µ3 is the 3^rd^ central moment.Skewness measures the asymmetry of the distribution of values about the Mean value. Depending on where the tail is elongated and the mass of the distribution is concentrated, this value can be positive or negative. |
| Total Energy | $\mathrm{TotalEnergy}=V_{voxel}\sum_{i=1}^{N} ({X\left( i \right)+c)}^{2}$  Here, $c$ is optional value, defined by “voxelArrayShift”, which shifts the intensities to prevent negative values in **X**. This ensures that voxels with the lowest gray values contribute the least to RMS, instead of voxels with gray level intensity closest to 0.Total Energy is the value of Energy feature scaled by the volume of the voxel in cubic mm. |
| Uniformity | $\mathrm{Uniformity}=\sum_{i=1}^{N_{I}} p({i)}^{2}$  Uniformity is a measure of the sum of the squares of each intensity value. This is a measure of the heterogeneity of the image array, where a greater uniformity implies a greater heterogeneity or a greater range of discrete intensity values. |
| Variance | $\mathrm{Variance}=\frac{1}{N}\sum_{i=1}^{N} ({X\left( i \right)-\bar{X})}^{2}$  Variance is the the mean of the squared distances of each intensity value from the Mean value. This is a measure of the spread of the distribution about the mean. By denition, variance = *σ_2_* |
| Notations:  **X** is an image of *N voxels* included in the ROI  P*i* is the first order histogram with *Nl* discrete intensity levels, in which *Nl* is the number of non-zero bins  p*i* is the normalized first order histogram and equal to $\frac{P_{i}}{\sum P_{i}}$(This definition is the same for the following sections) | |

| **GLCM feature** | **Definition** |
| --- | --- |
| Autocorrelation | Autocorrelation$=\sum_{i=1}^{N_{g}} \sum_{j=1}^{N_{g}} ijp(i,j)$  Autocorrelation is a measure of the magnitude of the neness and coarseness of texture. |
| Joint Average | $\mathrm{JointAverage}=u_{x}=\sum_{i=1}^{N_{g}} \sum_{j=1}^{N_{g}} p(i,j)i$  Returns the mean gray level intensity of the *i* distribution. |
| Cluster Prominence | $\mathrm{ClusterProminence}=\sum_{i=1}^{N_{g}} \sum_{j=1}^{N_{g}} {(i+j-u_{x}\left( i \right)-u_{y}\left( j \right))}^{4}p(i,j)$  ClusterProminence is a measure of the skewness and asymmetry of the GLCM.A higher values implies more asymmetry about the mean while a lower value indicates a peak near the mean value and less variation about the mean. |
| Cluster Shade | ClusterShade$=\sum_{i=1}^{N_{g}} \sum_{j=1}^{N_{g}} {(i+j-u_{x}\left( i \right)-u_{y}\left( j \right))}^{3}p(i,j)$  ClusterShade is a measure of the skewness and uniformity of the GLCM.A higher cluster shade implies greater asymmetry about the mean. |
| Cluster Tendency | ClusterTendency$=\sum_{i=1}^{N_{g}} \sum_{j=1}^{N_{g}} {(i+j-u_{x}\left( i \right)-u_{y}\left( j \right))}^{2}p(i,j)$  ClusterTendency is a measure of groupings of voxels with similar gray-level values. |
| Contrast | Contrast$=\sum_{i=1}^{N_{g}} \sum_{j=1}^{N_{g}} \left\vert i-j \right\vert^{2}p(i,j)$  Contrast is a measure of the local intensity variation,favoring values away from the diagonal(*i*=*j*). A larger value correlates with a greater disparity in intensity values among neighboring voxels. |
| Correlation | Correlation$=\frac{\sum_{i=1}^{N_{g}} \sum_{j=1}^{N_{g}} p\left( i,j \right)ij-\mu_{x}(i)u_{y}\left( j \right)}{\sigma_{x}{\left( i \right)\sigma}_{y}\left( j \right)}$  Correlation is a value between 0 (uncorrelated) and 1 (perfectlycorrelated) showing the linear dependency of gray level values to their respective voxels in the GLCM. |
| Difference Average | DifferenceAverage$=\sum_{k=0}^{N_{g}-1} kp_{x-y}(k)$  Dierence Average measures the relationship between occurrences of pairs with similar intensity values and occurrences of pairs with diering intensity values. |
| Difference Entropy | DifferenceEntropy$=\sum_{k=0}^{N_{g}-1} p_{x-y}(k){log}_{2}(p_{x-y}\left( k \right)+\epsilon)$  Dierence Entropy is a measure of the randomness/variability in neighborhood intensity value dierences. |
| Difference Variance | DifferenceVariance$=\sum_{k=0}^{N_{g}-1} ({1-DA)}^{2}p_{x-y}(k)$  Dierence Variance is a measure of heterogeneity that places higher weights on diering intensity level pairs that deviate more from the mean. |
| Joint Energy | JointEnergy$=\sum_{i=1}^{N_{g}} \sum_{j=1}^{N_{g}} \left[ p(i,j) \right]^{2}$  Energy is a measure of homogeneous patterns in the image. A greater Energy implies that there are more instances of intensity value pairs in the image that neighbor each other at higher frequencies. |
| Joint Entropy | JointEntropy$=-\sum_{i=1}^{N_{g}} \sum_{j=1}^{N_{g}} p(i,j){log}_{2}(p(i,j)+\in)$  Joint entropy is a measure of the randomness/variability in neighborhood intensity values. |
| Imc1 | Imc1$=\frac{HXY-HXY1}{max\{HX,HY\}}$ |
| Imc2 | Imc2$=\sqrt{1-e^{-2(HXY2-HXY)}}$ |
| Idm | Idm$=\sum_{i=1}^{N_{g}} \sum_{j=1}^{N_{g}} \frac{p(i,j)}{{1+\left\vert i-j \right\vert}^{2}}$  IDM (inverse dierence moment) is a measure of the local homogeneity of an image. IDM weights are the inverse of the Contrast weights (decreasing expo nentially from the diagonal *i* = *j* in the GLCM). |
| Idmn | Idmn$=\sum_{i=1}^{N_{g}} \sum_{j=1}^{N_{g}} \frac{p(i,j)}{1+(\frac{\left\vert i-j \right\vert^{2}}{N_{g}})}$  IDMN(inverse difference moment normalized) is a measure of the local homogeneity of an image. IDMN weights are the inverse of the Contrast weights (decreasing exponentially from the diagonal *i* = *j* in the GLCM). Unlike Homogeneity2, IDMN normalizes the square of the difference between neighboring intensity values by dividing over the square of the total number of discrete intensity values. |
| Id | Id$=\sum_{i=1}^{N_{g}} \sum_{j=1}^{N_{g}} \frac{p(i,j)}{1+\left\vert i-j \right\vert}$  ID (inverse dierence) is another measure of the local homogeneity of an image. With more uniform gray levels, the denominator will remain low, resulting in a higher overall value. |
| Idn | Idn$=\sum_{i=1}^{N_{g}} \sum_{j=1}^{N_{g}} \frac{p(i,j)}{1+(\frac{\left\vert i-j \right\vert}{N_{g}})}$  IDN(inverse difference normalized) is an other measure of the local homogeneity of an image. Unlike Homogeneity1, IDN normalizes the difference between the neighboring intensity values by dividing over the total number of discrete intensity values. |
| Inverse Variance | InverseVariance$=\sum_{i=1}^{N_{g}} \sum_{j=1}^{N_{g}} \frac{p(i,j)}{\left\vert i-j \right\vert^{2}},(i\neq j)$ |
| Maximum Probability | MaximumProbability$=max(p\left( i,j \right))$  Maximum Probability is occurrences of the most predominant pair of neighboring intensity values. |
| Sum Entropy | SumEntropy$=\sum_{K=2}^{2N_{g}} p_{x+y}(k){log}_{2} \left[ p_{x+y}(k)+\epsilon\right]$  Sum Entropy is a sum of neighborhood intensity value dierences. |
| Sum Squares | SumSquares$=\sum_{i=1}^{N_{g}} \sum_{j=1}^{N_{g}} \left( i-\mu_{x} \right)^{2}p(i,j)$  Sum of Squares or Variance is a measure in the distribution of neigboring in tensity level pairs about the mean intensity level in the GLCM. |
| Notations:  P(*i; j*) is the co-occurence matrix for *δ* (distance) and *α* (angle)  p(*i; j*) is the normalized co-occurence matrix  Ng is the number of discrete intensity levels in the image  $p_{x}\left( i \right)=\sum_{j=1}^{N_{g}} P(i,j)$is the marginal row probabilities  $p_{\mathrm{xy}}\left( j \right)=\sum_{j=1}^{N_{g}} P(i,j)$ is the marginal column probabilities  $u_{x}=\sum_{i=1}^{N_{g}} \sum_{j=1}^{N_{g}} P(i,j)i$ is the mean gray level intensity of *p_x_*  $u_{y}=\sum_{i=1}^{N_{g}} \sum_{j=1}^{N_{g}} P(i,j)j$ is the mean gray level intensity of *p_y_*  *σx* is the standard deviation of *p_x_*  *σy* is the standard deviation of *p_y_*  $p_{x+y}(k)=\sum_{i=1}^{N_{g}} \sum_{j=1}^{N_{g}} P(i,j)$, where *i + j* = k  $p_{x-y}(k)=\sum_{i=1}^{N_{g}} \sum_{j=1}^{N_{g}} P(i,j)$, where $\left\vert i-j \right\vert=k$  $HX=\sum_{i=1}^{N_{j}} p_{x}(i)\log_{2}(p_{x}\left( i \right)+\in)$ is the entropy of *p_x_*  $HY=\sum_{i=1}^{N_{j}} p_{y}(i)\log_{2}(p_{y}\left( i \right)+\in)$is the entropy of *p_y_*  $HXY=-\sum_{i=1}^{N_{g}} \sum_{j=1}^{N_{g}} p\left( i,j \right)\log_{2}\left( p\left( i,j \right)+\in\right)$is the entropy of p(*i; j*)  $HXY1=-\sum_{i=1}^{N_{g}} \sum_{j=1}^{N_{g}} p\left( i,j \right)\log_{2}\left( p\left( i,j \right)+\in\right)$  $HXY2=-\sum_{i=1}^{N_{g}} \sum_{j=1}^{N_{g}} p_{x}(i)p_{y}(j)\log_{2}\left( p_{x}(i)p_{y}(j)+\in\right)$ | |

| **GLRLM feature** | **Definition** |
| --- | --- |
| Gray Level NonUniformity | $GLN=\frac{\sum_{i=1}^{N_{g}} \left[ \sum_{j=1}^{N_{r}} \boldsymbol{P}(i,j\vert\theta) \right]^{2}}{\sum_{i=1}^{N_{g}} \sum_{j=1}^{N_{r}} \boldsymbol{P}(i,j\vert\theta)}$  GLN measures the similarity of gray-level intensity values in the image, where a lower GLN value correlates with a greater similarity in intensity values. |
| Gray Level NonUniformity Normalized | $GLNN=\frac{\sum_{i=1}^{N_{g}} {(\sum_{j=1}^{N_{r}} \boldsymbol{P}\left( i,j \vert\theta\right)}^{2}}{\sum_{i=1}^{N_{g}} \sum_{j=1}^{N_{r}} \boldsymbol{P}(i,j\vert\theta)}$  GLNN measures the similarity of gray-level intensity values in the image, where a lower GLNN value correlates with a greater similarity in intensity values.This is the normalized version of the GLN formula. |
| Gray Level Variance | $GLV=\sum_{i=1}^{N_{g}} \sum_{j=1}^{N_{r}} p\left( i,j \vert\theta\right))\left( i-\mu\right)^{2}$  GLV measures the variance in gray level intensity for the runs. |
| High Gray Level Run Emphasis | $HGLRE=\frac{\sum_{i=1}^{N_{g}} \sum_{j=1}^{N_{r}} \boldsymbol{P}(i,j\vert\theta)i^{2}}{\sum_{i=1}^{N_{g}} \sum_{j=1}^{N_{r}} \boldsymbol{P}(i,j\vert\theta)}$  HGLRE measures the distribution of the higher gray-level values, with a higher value indicating a greater concentration of high gray-level values in the image. |
| Long Run Emphasis | $LRE=\frac{\sum_{i=1}^{N_{g}} \sum_{j=1}^{N_{r}} \boldsymbol{P}(i,j\vert\theta)j^{2}}{\sum_{i=1}^{N_{g}} \sum_{j=1}^{N_{r}} \boldsymbol{P}(i,j\vert\theta)}$  LRE is a measure of the distribution of long run lengths, with a greater value indicative of longer run lengths and more coarse structural textures. |
| Long Run High Gray Level Emphasis | $LRHGLE=\frac{\sum_{i=1}^{N_{g}} \sum_{j=1}^{N_{r}} \boldsymbol{P}\left( i,j \vert\theta\right)i^{2}j^{2}}{\sum_{i=1}^{N_{g}} \sum_{j=1}^{N_{r}} \boldsymbol{P}(i,j\vert\theta)}$  LRHGLE measures the joint distribution of long run lengths with higher gray level values. |
| Long Run Low Gray Level Emphasis | $LRLGLE=\frac{\sum_{i=1}^{N_{g}} \sum_{j=1}^{N_{r}} \frac{\boldsymbol{P}\left( i,j \vert\theta\right)j^{2}}{i^{2}}}{\sum_{i=1}^{N_{g}} \sum_{j=1}^{N_{r}} \boldsymbol{P}(i,j\vert\theta)}$  LRLGLE measures the joint distribution of long run lengths with lower gray level values. |
| Low Gray Level Run Emphasis | $LGLRE=\frac{\sum_{i=1}^{N_{g}} \sum_{j=1}^{N_{r}} \frac{\boldsymbol{P}(i,j\vert\theta)}{i^{2}}}{\sum_{i=1}^{N_{g}} \sum_{j=1}^{N_{r}} \boldsymbol{P}(i,j\vert\theta)}$  LGLRE measures the distribution of low gray-level values, with a higher value indicating a greater concentration of low gray-level values in the image. |
| Run Entropy | $RE=\sum_{i=1}^{N_{g}} \sum_{j=1}^{N_{g}} p\left( i,j \vert\theta\right){log}_{2}(p\left( i,j \vert\theta\right)+\epsilon)$  Here, $\epsilon$ is an arbitrarily small positive number (≈ 2:2 × 10^-16^).RE measures the uncertainty/randomness in the distribution of run lengths and gray levels. A higher value indicates more heterofeaturesity in the texture patterns. |
| Run Length NonUniformity | $RLN=\frac{\sum_{j=1}^{N_{r}} {\sum_{i=1}^{N_{g}} \boldsymbol{(P}(i,j\vert\theta))}^{2}}{\sum_{i=1}^{N_{g}} \sum_{j=1}^{N_{r}} \boldsymbol{P}(i,j\vert\theta)}$  RLN measures the similarity of run lengths throughout the image, with a lower value indicating more homogeneity among run length sinthe image. |
| Run Length NonUniformity Normalized | $RLNN=\frac{\sum_{j=1}^{N_{r}} {(\sum_{i=1}^{N_{g}} \boldsymbol{P}(i,j\vert\theta))}^{2}}{\sum_{i=1}^{N_{g}} \sum_{j=1}^{N_{r}} \boldsymbol{P}(i,j\vert\theta)}$  RLNN measures the similarity of run lengths throughout the image, with a lower value indicating more homofeaturesity among run lengths in the image. This is the normalized version of the RLN formula. |
| Run Percentage | $RP=\sum_{i=1}^{N_{g}} \sum_{j=1}^{N_{r}} \frac{\boldsymbol{P}(i,j\vert\theta)}{N_{p}}$  RP measures the coarseness of the texture by taking the ratio of number of runs and number of voxels in the ROI. |
| Run Variance | $RV=\sum_{i=1}^{N_{g}} \sum_{j=1}^{N_{r}} p\left( i,j \vert\theta\right))\left( j-\mu\right)^{2}$  RV is a measure of the variance in runs for the run lengths. |
| Short Run Emphasis | $SRE=\frac{\sum_{i=1}^{N_{g}} \sum_{j=1}^{N_{r}} \left[ \frac{\boldsymbol{P}(i,j\vert\theta)}{i} \right]}{\sum_{i=1}^{N_{g}} \sum_{j=1}^{N_{r}} \boldsymbol{P}(i,j\vert\theta)}$  SRE is a measure of the distribution of short run lengths, with a greater value indicative of shorter run lengths and more fine textural textures. |
| Short Run High Gray Level Emphasis | $SRHGLE=\frac{\sum_{i=1}^{N_{g}} \sum_{j=1}^{N_{r}} \frac{\boldsymbol{P}\left( i,j \vert\theta\right)i^{2}}{j^{2}}}{\sum_{i=1}^{N_{g}} \sum_{j=1}^{N_{r}} \boldsymbol{P}(i,j\vert\theta)}$  SRHGLE measures the joint distribution of shorter run lengths with higher gray-level values. |
| Short Run Low Gray Level Emphasis | $\mathrm{SRLGLE}=\frac{\sum_{i=1}^{N_{g}} \sum_{j=1}^{N_{r}} \frac{\boldsymbol{P}\left( i,j \vert\theta\right)}{i^{2}j^{2}}}{\sum_{i=1}^{N_{g}} \sum_{j=1}^{N_{r}} \boldsymbol{P}(i,j\vert\theta)}$  SRLGLE measures the joint distribution of shorter run lengths with lowe rgray level values. |
| Notations:  $P\left( i,j \vert\theta\right)$ is the run length matrix of direction $\theta$  $p\left( i,j \vert\theta\right)$ is the normalized run length matrix  Ng is the number of discrete intensity values in the image  Nr is the number of discrete run lengths in the image  Np is the number of voxels in the image | |

| **GLSZM feature** | **Definition** |
| --- | --- |
| Gray Level NonUniformity | $GLN=\frac{\sum_{i=1}^{N_{g}} {(\sum_{j=1}^{N_{g}} \boldsymbol{P}\left( i,j \right))}^{2}}{\sum_{i=1}^{N_{g}} \sum_{j=1}^{N_{g}} \boldsymbol{P}\left( i,j \right)}$  GLN measures the variability of gray-level intensity values in the image, with a lower value indicating more homogeneity in intensity values. |
| Gray Level NonUniformity Normalized | $GLNN=\frac{\sum_{i=1}^{N_{g}} {(\sum_{j=1}^{N_{g}} \boldsymbol{P}\left( i,j \right))}^{2}}{\sum_{i=1}^{N_{g}} \sum_{j=1}^{N_{g}} \boldsymbol{P}\left( i,j \right)^{2}}$  GLNN measures the variability of gray-level intensity values in the image, with a lower value indicating a greater similarity in intensity values.This is the normalized version of the GLN formula. |
| Gray Level Variance | $GLV=\sum_{i=1}^{N_{g}} \sum_{j=1}^{N_{g}} p(i,j)\left( i-\mu\right)^{2}$  GLV measures the variance in gray level intensities for the zones. |
| High Gray Level Zone Emphasis | $HGLZE=\frac{\sum_{i=1}^{N_{g}} {\sum_{j=1}^{N_{s}} \boldsymbol{P}\left( i,j \right)i}^{2}}{\sum_{i=1}^{N_{g}} \sum_{j=1}^{N_{s}} P(i,j)}$  HGLZE measures the distribution of the higher gray-level values,with a higher value indicating a greater proportion of higher gray-level values and size zones in the image. |
| Large Area Emphasis | $LAE=\frac{\sum_{i=1}^{N_{g}} \sum_{j=1}^{N_{g}} \boldsymbol{P}(i,j)j^{2}}{\sum_{i=1}^{N_{g}} \sum_{j=1}^{N_{g}} \boldsymbol{P}(i,j)）}$  LAE is a measure of the distribution of large area size zones, with a greater value indicative of more larger size zones and more coarse textures. |
| Large Area High Gray Level Emphasis | $LAHGLE==\frac{\sum_{i=1}^{N_{g}} {\sum_{j=1}^{N_{S}} \boldsymbol{P}\left( i,j \right)i}^{2}j^{2}}{\sum_{i=1}^{N_{g}} \sum_{j=1}^{N_{s}} \boldsymbol{P}(i,j)}$  LAHGLE measures the proportion in the image of the joint distribution of larger size zones with higher gray-level values. |
| Large Area Low Gray Level Emphasis | $LALGLE=\frac{\sum_{i=1}^{N_{g}} \sum_{j=1}^{N_{s}} \frac{\boldsymbol{P}(i,j)j^{2}}{i^{2}}}{\sum_{i=1}^{N_{g}} \sum_{j=1}^{N_{s}} \boldsymbol{P}(i,j)）}$  LALGLE measures the proportion in the image of the joint distribution of larger size zones with lower gray-level values. |
| Low Gray Level Zone Emphasis | $LGLZE=\frac{\sum_{i=1}^{N_{g}} \sum_{j=1}^{N_{s}} \frac{\boldsymbol{P}(i,j)}{i^{2}}}{\sum_{i=1}^{N_{g}} \sum_{j=1}^{N_{s}} P(i,j)}$  LGLZE measures the distribution of lower gray-level size zones,with a higher value indicating a greater proportion of lower gray-level values and size zones in the image. |
| Size Zone NonUniformity | $SZN=\frac{\sum_{i=1}^{N_{g}} {(\sum_{j=1}^{N_{g}} \boldsymbol{P}\left( i,j \right))}^{2}}{\sum_{i=1}^{N_{g}} \sum_{j=1}^{N_{g}} \boldsymbol{P}(i,j)}$  SZN measures the variability of size zone volumes in the image, with a lower value indicating more homogeneity in size zone volumes. |
| Size Zone NonUniformity Normalized | $SZNN=\frac{\sum_{i=1}^{N_{g}} {(\sum_{j=1}^{N_{g}} \boldsymbol{P}\left( i,j \right))}^{2}}{\sum_{i=1}^{N_{g}} \sum_{j=1}^{N_{g}} {\boldsymbol{P}(i,j)}^{2}}$  SZNN measures the variability of size zone volumes throughout the image, with a lower value indicating more homogeneity among zone size volumes in the image. This is the normalized version of the SZN formula. |
| Small Area Emphasis | $SAE=\frac{\sum_{i=1}^{N_{g}} \sum_{j=1}^{N_{g}} \frac{\boldsymbol{P}(i,j)}{j^{2}}}{\sum_{i=1}^{N_{g}} \sum_{j=1}^{N_{g}} \boldsymbol{P}(i,j)）}$  SAE is a measure of the distribution of small size zones, with a greater value indicative of more smaller size zones and more fine textures. |
| Small Area High Gray Level Emphasis | $SAHGLE=\frac{\sum_{i=1}^{N_{g}} \sum_{j=1}^{N_{S}} \frac{\boldsymbol{P}(i,j)i^{2}}{j^{2}}}{\sum_{i=1}^{N_{g}} \sum_{j=1}^{N_{s}} \boldsymbol{P}(i,j)}$  SAHGLE measures the proportion in the image of the joint distribution of smaller size zones with higher gray-level values. |
| Small Area Low Gray Level Emphasis | $SALGLE=\frac{\sum_{i=1}^{N_{g}} \sum_{j=1}^{N_{s}} \frac{\boldsymbol{P}(i,j)}{i^{2}j^{2}}}{\sum_{i=1}^{N_{g}} \sum_{j=1}^{N_{s}} \boldsymbol{P}(i,j)}$  SALGLE measures the proportion in the image of the joint distribution of smaller size zones with lower gray-level values. |
| Zone Entropy | $ZE=-\sum_{i=1}^{N_{g}} \sum_{j=1}^{N_{g}} p(i,j){log}_{2}(p\left( i,j \right)+\in)$  Here, $\epsilon$ is an arbitrarily small positive number (≈ 2:2 × 10^-16^).  ZE measures the uncertainty/randomness in the distribution of zone sizes and gray levels. A higher value indicates more heterogeneneity in the texture patterns. |
| Zone Percentage | $ZP=\sum_{i=1}^{N_{g}} \sum_{j=1}^{N_{g}} \frac{\boldsymbol{P}(i,j)}{N_{p}}$  ZP measures the coarseness of the texture by taking the ratio of number of zones and number of voxels in the ROI. |
| Zone Variance | $ZV=\sum_{i=1}^{N_{g}} \sum_{j=1}^{N_{g}} p(i,j)\left( j-\mu\right)^{2}$  ZV measures the variance in zone size volumes for the zones. |
| Note:  P (*i; j*) is the size zone matrix  p (*i; j*) is the normalized size zone matrix  N*g* is the number of discrete intensity values in the image  N*s* is the number of discrete zone sizes in the image  N*p* is the number of *voxels* in the image | |

| **GLDM feature** | **Definition** |
| --- | --- |
| Dependence Entropy | $DE=-\sum_{i=1}^{N_{g}} \sum_{j=1}^{N_{d}} p\left( i,j \right){log}_{2} \left[ p\left( i,j \right)+\epsilon\right]$ |
| Dependence NonUniformity | DN$=\frac{\sum_{j=1}^{Nd} {(\sum_{i=1}^{Ng} p\left( i,j \right))}^{2}}{Nz}$  Measures the similarity of dependence throughout the image, with a lower value indicating more homogeneity among dependencies in the image. |
| Dependence NonUniformity Normalized | DNN$=\frac{\sum_{j=1}^{Nd} {(\sum_{i=1}^{Ng} p\left( i,j \right))}^{2}}{Nz}$  Measures the similarity of dependence throughout the image, with a lower value indicating more homogeneity among dependencies in the image. This is the normalized version of the DLN formula. |
| Dependence Variance | $DV=\sum_{i=1}^{N_{g}} \sum_{j=1}^{N_{d}} p\left( i,j \right)\left( i-\mu\right)^{2}$  Measures the variance in dependence size in the image. |
| Gray Level NonUniformity | $\mathrm{GLN}=\frac{\sum_{j=1}^{Ng} {(\sum_{i=1}^{Nd} p\left( i,j \right))}^{2}}{Nz}$  Measures the similarity of gray-level intensity values in the image, where a lower GLN value correlates with a greater similarity in intensity values. |
| Gray Level Variance | $\mathrm{GLV}=\sum_{i=1}^{N_{g}} \sum_{j=1}^{N_{d}} p\left( i,j \right)\left( i-\mu\right)^{2}$  Measures the variance in grey level in the image. |
| High Gray Level Emphasis | $\mathrm{HGLE}=\frac{\sum_{j=1}^{Ng} {\sum_{i=1}^{Nd} p\left( i,j \right)i}^{2}}{Nz}$  Measures the distribution of the higher gray-level values, with a higher value indicating a greater concentration of high gray-level values in the image. |
| Large Dependence Emphasis | $\mathrm{LDE}=\frac{\sum_{j=1}^{Ng} {(\sum_{i=1}^{Nd} p\left( i,j \right)j}^{2}}{Nz}$  A measure of the distribution of large dependencies, with a greater value indicative of larger dependence and more homogeneous textures. |
| Large Dependence High Gray Level Emphasis | LDHGLE $=\frac{\sum_{j=1}^{Ng} {(\sum_{i=1}^{Nd} p\left( i,j \right)ij}^{2}}{Nz}$  Measures the joint distribution of large dependence with higher gray-level values. |
| Large Dependence Low Gray Level Emphasis | LDLGLE$=\frac{\sum_{j=1}^{Ng} {\sum_{i=1}^{Nd} \frac{\boldsymbol{P}(i,j)}{i^{2}}}}{Nz}$  Measures the joint distribution of large dependence with lower gray-level values. |
| Low Gray Level Emphasis | LGLE$=\frac{\sum_{i=1}^{N_{g}} \sum_{j=1}^{N_{d}} \frac{\boldsymbol{P}(i,j)}{i^{2}}}{Nz}$  Measures the distribution of low gray-level values,with a higher value indicating a greater concentration of low gray-level values in the image. |
| Small Dependence Emphasis | SDE$=\frac{\sum_{i=1}^{N_{g}} \sum_{j=1}^{N_{d}} \frac{\boldsymbol{P}(i,j)}{i^{2}}}{Nz}$  A measure of the distribution of small dependencies,with a greater value indicative of smaller dependence and less homogeneous textures. |
| Small Dependence High Gray Level Emphasis | Measures the joint distribution of small dependence with higher gray-level values. |
| Small Dependence Low Gray Level Emphasis | SDLGLE$=\frac{\sum_{i=1}^{N_{g}} \sum_{j=1}^{N_{d}} \frac{\boldsymbol{P}(i,j)}{i^{2}j^{2}}}{Nz}$  Measures the joint distribution of small dependence with lowe rgray-level values. |
| N*g* be the number of discreet intensity values in the image  N*d* be the number of discreet dependency sizes in the image  N*z* be the number of dependency zones in the image, which is equal to $\sum_{i=1}^{Ng} {\sum_{j=1}^{Nd} \mathbf{P}\left( i,j \right)}$  **P**(*i*, *j*) be the dependence matrix  p(*i*, *j*) be the normalized dependence matrix, defined as p(*i*, *j*) = $\frac{\mathbf{P}(i,j)}{Nz}$ | |

**Table S5** Consistency analysis of radiological features among observers

|  | **Kappa** (95% CI) |  |  | **ICC** (95% CI) |
| --- | --- | --- | --- | --- |
| Density type | 0.960 (0.931-0.989) |  | Tumor size (mm) | 0.934 (0.911-0.950) |
| Distal ribbon sign | 0.968 (0.939-0.996) |  | Solid component size (mm) | 0.935 (0.919-0.947) |
| Shape | 0.887 (0.834-0.941) |  |  |  |
| Lobulation | 0.949 (0.912-0.986) |  |  |  |
| Spiculation | 0.953 (0.916-0.990) |  |  |  |
| Tumor-lung interface | 0.886 (0.803-0.969 ) |  |  |  |
| Bronchial change | 0.925 (0.888-0.962) |  |  |  |
| Vacuole | 0.963 (0.934-0.992) |  |  |  |
| Cavity/Cystic airspace | 0.881 (0.800-0.962) |  |  |  |
| Vascular convergence | 0.875 (0.817-0.933) |  |  |  |
| Halo sign | 0.852 (0.709-0.995) |  |  |  |
| Pleural tags | 0.960 (0.933-0.987) |  |  |  |
| Pleural indentation | 0.970 (0.947-0.994) |  |  |  |
| Satellite lesions | 0.925 (0.859-0.990) |  |  |  |
| ELLC | 1.000 |  |  |  |
| ERL | 1.000 |  |  |  |

**Table S6** The training cohort included collinearity test of multivariate logistic regression analysis variables

|  | Collinearity test statistics | |
| --- | --- | --- |
|  | Tolerance | Variance inflation factor |
| Sex | 0.669 | 1.494 |
| Smoking status | 0.664 | 1.505 |
| CEA | 0.873 | 1.146 |
| Clinical T stage | 0.400 | 2.502 |
| CTR | 0.528 | 1.895 |
| Density type | 0.601 | 1.663 |
| Tumor-lung interface | 0.846 | 1.182 |
| Spiculation | 0.663 | 1.508 |
| Bronchial change | 0.844 | 1.185 |
| Pleural tags | 0.742 | 1.347 |
| Pleural indentation | 0.802 | 1.246 |
| Vascular convergence | 0.758 | 1.319 |
| Halo sign | 0.814 | 1.228 |
| Distal ribbon sign | 0.674 | 1.484 |

**Table S7** Inter-observer and intra-observer ICC > 0.80

|  | ICC > 0.80 | |
| --- | --- | --- |
|  | repeatability | ratio |
| GTV | 1048/1218 | 86.0% |
| PTV5 | 1148/1218 | 94.3% |
| PTV10 | 1186/1218 | 97.4% |
| PTV15 | 1185/1218 | 97.3% |
| PTV20 | 1215/1218 | 99.8% |
| GPTV5 | 1192/1218 | 97.9% |
| GPTV10 | 1192/1218 | 97.9% |
| GPTV15 | 1189/1218 | 97.6% |
| GPTV20 | 1204/1218 | 98.9% |

**Table S8** The features used for radiomics model construction and their ICC

**① GTV**

Radscore=-0.474*original_firstorder_90Percentile+0.982*wavelet_LLL_glszm_LargeAreaHighGrayLevelEmphasis+-1.322*log_sigma_5_0_mm_3D_glszm_SmallAreaLowGrayLevelEmphasis+-0.232*original_gldm_LargeDependenceHighGrayLevelEmphasis+0.398*log_sigma_1_0_mm_3D_glszm_SizeZoneNonUniformity+0.426*wavelet_LLL_gldm_LargeDependenceLowGrayLevelEmphasis+0.173*original_glszm_SizeZoneNonUniformityNormalized+0.334*log_sigma_5_0_mm_3D_glszm_LowGrayLevelZoneEmphasis+1.15*wavelet_LLL_firstorder_Maximum+-0.051*original_glcm_ClusterShade+-0.608*wavelet_HLL_firstorder_Mean+-0.529*wavelet_LLL_gldm_LargeDependenceHighGrayLevelEmphasis+0.427*wavelet_HHL_glcm_Correlation+0.183*log_sigma_3_0_mm_3D_glszm_SizeZoneNonUniformity+0.402*log_sigma_3_0_mm_3D_glcm_Imc1+-0.344*wavelet_LLL_gldm_LowGrayLevelEmphasis+0.849*original_glszm_LargeAreaHighGrayLevelEmphasis+-0.366*original_firstorder_Skewness+-0.282*wavelet_LHL_glszm_GrayLevelVariance + -1.876

|  | ICC | |
| --- | --- | --- |
|  | Inter | Intra |
| original_firstorder_90Percentile | 0.989 | 0.995 |
| wavelet_LLL_glszm_LargeAreaHighGrayLevelEmphasis | 0.924 | 0.995 |
| log_sigma_5_0_mm_3D_glszm_SmallAreaLowGrayLevelEmphasis | 0.861 | 0.927 |
| original_gldm_LargeDependenceHighGrayLevelEmphasis | 0.936 | 0.967 |
| log_sigma_1_0_mm_3D_glszm_SizeZoneNonUniformity | 0.928 | 0.847 |
| wavelet_LLL_gldm_LargeDependenceLowGrayLevelEmphasis | 0.801 | 0.830 |
| original_glszm_SizeZoneNonUniformityNormalized | 0.943 | 0.928 |
| log_sigma_5_0_mm_3D_glszm_LowGrayLevelZoneEmphasis | 0.912 | 0.968 |
| wavelet_LLL_firstorder_Maximum | 0.972 | 0.889 |
| original_glcm_ClusterShade | 0.871 | 0.593 |
| wavelet_HLL_firstorder_Mean | 0.842 | 0.668 |
| wavelet_LLL_gldm_LargeDependenceHighGrayLevelEmphasis | 0.955 | 0.971 |
| wavelet_HHL_glcm_Correlation | 0.960 | 0.924 |
| log_sigma_3_0_mm_3D_glszm_SizeZoneNonUniformity | 0.959 | 0.956 |
| log_sigma_3_0_mm_3D_glcm_Imc1 | 0.926 | 0.898 |
| wavelet_LLL_gldm_LowGrayLevelEmphasis | 0.812 | 0.808 |
| original_glszm_LargeAreaHighGrayLevelEmphasis | 0.908 | 0.978 |
| original_firstorder_Skewness | 0.952 | 0.886 |
| wavelet_LHL_glszm_GrayLevelVariance | 0.990 | 0.982 |

**② PTV5**  Radscore=-0.643*log_sigma_2_0_mm_3D_gldm_DependenceVariance+0.85*log_sigma_3_0_mm_3D_glcm_Imc1+-0.256*log_sigma_2_0_mm_3D_gldm_LargeDependenceLowGrayLevelEmphasis+0.239*log_sigma_5_0_mm_3D_glszm_GrayLevelVariance+0.107*log_sigma_4_0_mm_3D_gldm_LargeDependenceLowGrayLevelEmphasis+-0.296*wavelet_HLH_firstorder_Kurtosis+0.424*wavelet_LLL_gldm_LargeDependenceHighGrayLevelEmphasis+0.218*log_sigma_2_0_mm_3D_glcm_Imc1+0.351*original_glcm_Correlation+0.301*wavelet_HHH_glcm_Imc1+0.383*log_sigma_5_0_mm_3D_glrlm_RunLengthNonUniformity+-0.221*log_sigma_5_0_mm_3D_gldm_DependenceVariance+0.24*wavelet_HHH_glszm_SizeZoneNonUniformityNormalized+0.473*wavelet_LLL_firstorder_Skewness+0.484*log_sigma_5_0_mm_3D_gldm_LargeDependenceLowGrayLevelEmphasis+-0.086*log_sigma_4_0_mm_3D_glcm_Imc1 + -1.732

|  | ICC | |
| --- | --- | --- |
|  | Inter | Intra |
| log_sigma_2_0_mm_3D_gldm_DependenceVariance | 0.972 | 0.962 |
| log_sigma_3_0_mm_3D_glcm_Imc1 | 0.913 | 0.905 |
| log_sigma_2_0_mm_3D_gldm_LargeDependenceLowGrayLevelEmphasis | 0.959 | 0.920 |
| log_sigma_5_0_mm_3D_glszm_GrayLevelVariance | 0.948 | 0.923 |
| log_sigma_4_0_mm_3D_gldm_LargeDependenceLowGrayLevelEmphasis | 0.970 | 0.816 |
| wavelet_HLH_firstorder_Kurtosis | 0.920 | 0.964 |
| wavelet_LLL_gldm_LargeDependenceHighGrayLevelEmphasis | 0.996 | 0.977 |
| log_sigma_2_0_mm_3D_glcm_Imc1 | 0.895 | 0.791 |
| original_glcm_Correlation | 0.928 | 0.970 |
| wavelet_HHH_glcm_Imc1 | 0.977 | 0.969 |
| log_sigma_5_0_mm_3D_glrlm_RunLengthNonUniformity | 0.996 | 0.989 |
| log_sigma_5_0_mm_3D_gldm_DependenceVariance | 0.828 | 0.814 |
| wavelet_HHH_glszm_SizeZoneNonUniformityNormalized | 0.754 | 0.839 |
| wavelet_LLL_firstorder_Skewness | 0.887 | 0.887 |
| log_sigma_5_0_mm_3D_gldm_LargeDependenceLowGrayLevelEmphasis | 0.980 | 0.949 |
| log_sigma_4_0_mm_3D_glcm_Imc1 | 0.883 | 0.892 |

**③ PTV10** Radscore=-0.031*log_sigma_3_0_mm_3D_gldm_DependenceVariance+0.159*log_sigma_3_0_mm_3D_glszm_ZoneEntropy+0.188*log_sigma_5_0_mm_3D_glszm_GrayLevelVariance+0.188*wavelet_HHH_glcm_Imc1+0.248*wavelet_LLL_gldm_DependenceEntropy+-0.06*log_sigma_3_0_mm_3D_gldm_LargeDependenceLowGrayLevelEmphasis+0.411*log_sigma_3_0_mm_3D_glcm_Imc1+-0.062*wavelet_LHH_glcm_Imc2+0.342*log_sigma_4_0_mm_3D_glrlm_RunLengthNonUniformity + -1.353

|  | ICC | |
| --- | --- | --- |
|  | Inter | Intra |
| log_sigma_3_0_mm_3D_gldm_DependenceVariance | 0.989 | 0.983 |
| log_sigma_3_0_mm_3D_glszm_ZoneEntropy | 0.960 | 0.950 |
| log_sigma_5_0_mm_3D_glszm_GrayLevelVariance | 0.910 | 0.872 |
| wavelet_HHH_glcm_Imc1 | 0.994 | 0.998 |
| wavelet_LLL_gldm_DependenceEntropy | 0.990 | 0.989 |
| log_sigma_3_0_mm_3D_gldm_LargeDependenceLowGrayLevelEmphasis | 0.925 | 0.889 |
| log_sigma_3_0_mm_3D_glcm_Imc1 | 0.981 | 0.952 |
| wavelet_LHH_glcm_Imc2 | 0.997 | 0.994 |
| log_sigma_4_0_mm_3D_glrlm_RunLengthNonUniformity | 0.995 | 0.997 |

**④ PTV15** Radscore=0.226*log_sigma_5_0_mm_3D_glszm_ZoneEntropy+-0.177*wavelet_HLH_firstorder_Kurtosis+0.372*original_shape_LeastAxisLength+0.224*log_sigma_3_0_mm_3D_glrlm_HighGrayLevelRunEmphasis+0.268*wavelet_LLL_gldm_DependenceEntropy+0.223*wavelet_HLL_glszm_ZoneEntropy+0.101*original_glcm_ClusterShade+-0.286*log_sigma_2_0_mm_3D_glszm_SmallAreaLowGrayLevelEmphasis+-0.329*wavelet_HHH_glcm_Imc2+-0.163*wavelet_LHH_glcm_Imc2+0.261*log_sigma_3_0_mm_3D_glcm_Imc1+-0.073*log_sigma_3_0_mm_3D_glszm_GrayLevelNonUniformityNormalized+-0.061*wavelet_LLH_glcm_Idmn+0.317*wavelet_HHH_glszm_SizeZoneNonUniformityNormalized+-0.011*log_sigma_5_0_mm_3D_firstorder_Minimum + -1.468

|  | ICC | |
| --- | --- | --- |
|  | Inter | Intra |
| log_sigma_5_0_mm_3D_glszm_ZoneEntropy | 0.986 | 0.982 |
| wavelet_HLH_firstorder_Kurtosis | 0.993 | 0.998 |
| original_shape_LeastAxisLength | 0.985 | 0.990 |
| log_sigma_3_0_mm_3D_glrlm_HighGrayLevelRunEmphasis | 0.953 | 0.965 |
| wavelet_LLL_gldm_DependenceEntropy | 0.996 | 0.996 |
| wavelet_HLL_glszm_ZoneEntropy | 0.995 | 0.997 |
| original_glcm_ClusterShade | 0.989 | 0.993 |
| log_sigma_2_0_mm_3D_glszm_SmallAreaLowGrayLevelEmphasis | 0.953 | 0.987 |
| wavelet_HHH_glcm_Imc2 | 0.999 | 1.000 |
| wavelet_LHH_glcm_Imc2 | 0.997 | 0.998 |
| log_sigma_3_0_mm_3D_glcm_Imc1 | 0.992 | 0.980 |
| log_sigma_3_0_mm_3D_glszm_GrayLevelNonUniformityNormalized | 0.988 | 0.945 |
| wavelet_LLH_glcm_Idmn | 0.842 | 0.977 |
| wavelet_HHH_glszm_SizeZoneNonUniformityNormalized | 0.980 | 0.982 |
| log_sigma_5_0_mm_3D_firstorder_Minimum | 0.967 | 0.845 |

**⑤ PTV20** Radscore=0.278*wavelet_LLL_firstorder_Kurtosis+0.056*wavelet_HLL_glszm_ZoneEntropy+0.134*original_shape_MinorAxisLength+-0.359*wavelet_LLH_glrlm_LongRunHighGrayLevelEmphasis+-0.021*log_sigma_5_0_mm_3D_gldm_LargeDependenceLowGrayLevelEmphasis+0.201*original_glcm_ClusterShade+-0.477*log_sigma_2_0_mm_3D_glszm_SmallAreaLowGrayLevelEmphasis+-0.053*wavelet_LLL_glszm_HighGrayLevelZoneEmphasis+0.513*wavelet_LLL_gldm_DependenceEntropy+-0.26*wavelet_LHH_glcm_Imc2+-0.234*log_sigma_3_0_mm_3D_gldm_LargeDependenceLowGrayLevelEmphasis+-0.404*wavelet_LLH_glcm_ClusterShade+-0.138*wavelet_HLL_glcm_Idmn+0.247*log_sigma_2_0_mm_3D_glszm_GrayLevelNonUniformity+0.468*wavelet_HHH_glszm_SizeZoneNonUniformityNormalized+-0.207*log_sigma_5_0_mm_3D_glszm_LargeAreaLowGrayLevelEmphasis + -1.535

|  | ICC | |
| --- | --- | --- |
|  | Inter | Intra |
| wavelet_LLL_firstorder_Kurtosis | 0.994 | 0.994 |
| wavelet_HLL_glszm_ZoneEntropy | 0.997 | 0.999 |
| original_shape_MinorAxisLength | 0.966 | 0.967 |
| wavelet_LLH_glrlm_LongRunHighGrayLevelEmphasis | 0.996 | 1.000 |
| log_sigma_5_0_mm_3D_gldm_LargeDependenceLowGrayLevelEmphasis | 0.960 | 0.882 |
| original_glcm_ClusterShade | 0.995 | 0.998 |
| log_sigma_2_0_mm_3D_glszm_SmallAreaLowGrayLevelEmphasis | 0.969 | 0.917 |
| wavelet_LLL_glszm_HighGrayLevelZoneEmphasis | 0.985 | 0.999 |
| wavelet_LLL_gldm_DependenceEntropy | 0.998 | 0.998 |
| wavelet_LHH_glcm_Imc2 | 0.999 | 0.999 |
| log_sigma_3_0_mm_3D_gldm_LargeDependenceLowGrayLevelEmphasis | 0.979 | 0.945 |
| wavelet_LLH_glcm_ClusterShade | 0.997 | 0.999 |
| wavelet_HLL_glcm_Idmn | 0.999 | 0.996 |
| log_sigma_2_0_mm_3D_glszm_GrayLevelNonUniformity | 0.988 | 0.993 |
| wavelet_HHH_glszm_SizeZoneNonUniformityNormalized | 0.994 | 0.994 |
| log_sigma_5_0_mm_3D_glszm_LargeAreaLowGrayLevelEmphasis | 0.997 | 0.998 |

**⑥ GPTV5**  Radscore=-0.539*log_sigma_2_0_mm_3D_glcm_ClusterShade+0.363*wavelet_LLL_gldm_LargeDependenceHighGrayLevelEmphasis+-0.143*log_sigma_2_0_mm_3D_glcm_MaximumProbability+-0.139*log_sigma_2_0_mm_3D_gldm_LargeDependenceLowGrayLevelEmphasis+0.103*original_glcm_Correlation+-0.349*log_sigma_5_0_mm_3D_glszm_SmallAreaLowGrayLevelEmphasis+0.176*wavelet_LLL_firstorder_Maximum+0.4*wavelet_LLL_glszm_LargeAreaHighGrayLevelEmphasis+0.063*log_sigma_3_0_mm_3D_glszm_ZoneEntropy+-0.26*log_sigma_1_0_mm_3D_glszm_SmallAreaLowGrayLevelEmphasis+-0.079*log_sigma_5_0_mm_3D_gldm_DependenceVariance + -1.523

|  | ICC | |
| --- | --- | --- |
|  | Inter | Intra |
| log_sigma_2_0_mm_3D_glcm_ClusterShade | 0.988 | 0.984 |
| wavelet_LLL_gldm_LargeDependenceHighGrayLevelEmphasis | 0.993 | 0.987 |
| log_sigma_2_0_mm_3D_glcm_MaximumProbability | 0.981 | 0.981 |
| log_sigma_2_0_mm_3D_gldm_LargeDependenceLowGrayLevelEmphasis | 0.993 | 0.990 |
| original_glcm_Correlation | 0.996 | 0.996 |
| log_sigma_5_0_mm_3D_glszm_SmallAreaLowGrayLevelEmphasis | 0.987 | 0.896 |
| wavelet_LLL_firstorder_Maximum | 0.972 | 0.875 |
| wavelet_LLL_glszm_LargeAreaHighGrayLevelEmphasis | 0.995 | 0.997 |
| log_sigma_3_0_mm_3D_glszm_ZoneEntropy | 0.992 | 0.996 |
| log_sigma_1_0_mm_3D_glszm_SmallAreaLowGrayLevelEmphasis | 0.874 | 0.988 |
| log_sigma_5_0_mm_3D_gldm_DependenceVariance | 0.826 | 0.793 |

**⑦ GPTV10**  Radscore=-0.047*log_sigma_5_0_mm_3D_glszm_SmallAreaLowGrayLevelEmphasis+-0.403*log_sigma_2_0_mm_3D_gldm_LargeDependenceLowGrayLevelEmphasis+-0.574*log_sigma_2_0_mm_3D_glcm_ClusterShade+0.277*log_sigma_5_0_mm_3D_glcm_Imc2+-0.458*log_sigma_3_0_mm_3D_firstorder_10Percentile+0.077*log_sigma_3_0_mm_3D_glszm_ZoneEntropy+0.198*log_sigma_2_0_mm_3D_glcm_ClusterProminence+0.1*original_firstorder_Mean+0.288*wavelet_LLL_gldm_LargeDependenceHighGrayLevelEmphasis+0.037*log_sigma_3_0_mm_3D_glszm_GrayLevelVariance + -1.597

|  | ICC | |
| --- | --- | --- |
|  | Inter | Intra |
| log_sigma_5_0_mm_3D_glszm_SmallAreaLowGrayLevelEmphasis | 0.820 | 0.899 |
| log_sigma_2_0_mm_3D_gldm_LargeDependenceLowGrayLevelEmphasis | 0.999 | 0.988 |
| log_sigma_2_0_mm_3D_glcm_ClusterShade | 0.990 | 0.987 |
| log_sigma_5_0_mm_3D_glcm_Imc2 | 0.998 | 0.998 |
| log_sigma_3_0_mm_3D_firstorder_10Percentile | 0.989 | 0.953 |
| log_sigma_3_0_mm_3D_glszm_ZoneEntropy | 0.987 | 0.994 |
| log_sigma_2_0_mm_3D_glcm_ClusterProminence | 0.997 | 0.990 |
| original_firstorder_Mean | 0.998 | 0.996 |
| wavelet_LLL_gldm_LargeDependenceHighGrayLevelEmphasis | 0.997 | 0.990 |
| log_sigma_3_0_mm_3D_glszm_GrayLevelVariance | 0.989 | 0.996 |

**⑧ GPTV15**

Radscore=-0.091*log_sigma_5_0_mm_3D_glszm_SmallAreaLowGrayLevelEmphasis+-1.363*log_sigma_2_0_mm_3D_glcm_ClusterShade+0.457*log_sigma_5_0_mm_3D_glszm_ZoneEntropy+-0.199*original_glszm_SmallAreaHighGrayLevelEmphasis+0.31*original_glcm_Correlation+-0.25*log_sigma_4_0_mm_3D_firstorder_10Percentile+0.358*wavelet_LLL_gldm_LargeDependenceHighGrayLevelEmphasis+-0.284*wavelet_HLH_firstorder_Kurtosis+0.374*log_sigma_5_0_mm_3D_glcm_Correlation+0.707*log_sigma_3_0_mm_3D_glcm_ClusterShade+0.026*original_firstorder_RootMeanSquared+0.991*log_sigma_2_0_mm_3D_gldm_SmallDependenceHighGrayLevelEmphasis + -1.731

|  | ICC > 0.80 | |
| --- | --- | --- |
|  | Inter | Intra |
| log_sigma_5_0_mm_3D_glszm_SmallAreaLowGrayLevelEmphasis | 0.923 | 0.868 |
| log_sigma_2_0_mm_3D_glcm_ClusterShade | 0.998 | 0.993 |
| log_sigma_5_0_mm_3D_glszm_ZoneEntropy | 0.994 | 0.993 |
| original_glszm_SmallAreaHighGrayLevelEmphasis | 0.999 | 0.573 |
| original_glcm_Correlation | 0.999 | 0.998 |
| log_sigma_4_0_mm_3D_firstorder_10Percentile | 0.988 | 0.990 |
| wavelet_LLL_gldm_LargeDependenceHighGrayLevelEmphasis | 0.998 | 0.993 |
| wavelet_HLH_firstorder_Kurtosis | 0.995 | 0.999 |
| log_sigma_5_0_mm_3D_glcm_Correlation | 0.999 | 0.997 |
| log_sigma_3_0_mm_3D_glcm_ClusterShade | 0.998 | 0.993 |
| original_firstorder_RootMeanSquared | 0.999 | 0.998 |
| log_sigma_2_0_mm_3D_gldm_SmallDependenceHighGrayLevelEmphasis | 0.999 | 0.999 |

**⑨ GPTV20**

Radscore=0.701*original_glcm_ClusterProminence+-0.001*log_sigma_2_0_mm_3D_gldm_LargeDependenceLowGrayLevelEmphasis + -1.188

|  | ICC 0.80 | | |
| --- | --- | --- | --- |
|  | Inter | Intra | |
| original_glcm_ClusterProminence | 1.000 | | 0.998 |
| log_sigma_2_0_mm_3D_gldm_LargeDependenceLowGrayLevelEmphasis | 1.000 | | 0.985 |

**Table S9** DeLong test results of GPTV10 compared with the other radiomics models in the three cohorts

| Model | Cohort | z-value | *P* value |
| --- | --- | --- | --- |
| GPTV10 vs GTV | Training | 0.567 | 0.5706 |
|  | Internal validation | 1.506 | 0.1320 |
|  | External validation | 1.277 | 0.2016 |
| GPTV10 vs PTV5 | Training | 0.750 | 0.4530 |
|  | Internal validation | 2.782 | 0.0054 |
|  | External validation | 3.611 | 0.0003 |
| GPTV10 vs PTV10 | Training | 3.208 | 0.0013 |
|  | Internal validation | 2.455 | 0.0141 |
|  | External validation | 4.158 | *P* < 0.001 |
| GPTV10 vs PTV15 | Training | 1.361 | 0.1735 |
|  | Internal validation | 3.359 | 0.0008 |
|  | External validation | 3.315 | 0.0009 |
| GPTV10 vs PTV20 | Training | 1.774 | 0.0761 |
|  | Internal validation | 3.573 | 0.0004 |
|  | External validation | 4.589 | *P* < 0.001 |
| GPTV10 vs GPTV5 | Training | 0.724 | 0.4694 |
|  | Internal validation | 1.327 | 0.1844 |
|  | External validation | 0.503 | 0.6147 |
| GPTV10 vs GPTV15 | Training | 1.502 | 0.1331 |
|  | Internal validation | 1.512 | 0.1305 |
|  | External validation | 3.387 | 0.0007 |
| GPTV10 vs GPTV20 | Training | 3.573 | 0.0004 |
|  | Internal validation | 0.665 | 0.5061 |
|  | External validation | 0.100 | 0.9202 |

**Table S10** DeLong test results of GTV and PTV radiomics models in the three cohorts

| Model | Cohort | z-value | *P* value |
| --- | --- | --- | --- |
| GTV vs PTV5 | Training | 0.968 | 0.3330 |
|  | Internal validation | 1.306 | 0.1916 |
|  | External validation | 2.397 | 0.0165 |
| GTV vs PTV10 | Training | 2.887 | 0.0039 |
|  | Internal validation | 1.051 | 0.2931 |
|  | External validation | 2.853 | 0.0043 |
| GTV vs PTV15 | Training | 1.554 | 0.1202 |
|  | Internal validation | 2.147 | 0.0318 |
|  | External validation | 2.670 | 0.0076 |
| GTV vs PTV20 | Training | 1.910 | 0.0561 |
|  | Internal validation | 2.337 | 0.0194 |
|  | External validation | 3.571 | 0.0004 |

**Table S11** DeLong test results of GTV and GPTV radiomics models in the three cohorts

| Model | Cohort | z-value | *P* value |
| --- | --- | --- | --- |
| GTV vs GPTV5 | Training | 1.053 | 0.2923 |
|  | Internal validation | 0.842 | 0.3999 |
|  | External validation | 0.868 | 0.3853 |
| GTV vs GPTV10 | Training | 0.567 | 0.5706 |
|  | Internal validation | 1.506 | 0.1320 |
|  | External validation | 1.277 | 0.2016 |
| GTV vs GPTV15 | Training | 0.335 | 0.7379 |
|  | Internal validation | 0.512 | 0.6089 |
|  | External validation | 0.979 | 0.3276 |
| GTV vs GPTV20 | Training | 3.173 | 0.0015 |
|  | Internal validation | 0.861 | 0.3891 |
|  | External validation | 1.541 | 0.1232 |

**Table S12** DeLong test results of clinical model, GPTV10 radiomics model and combined model in the three cohorts

| Model | Cohort | z-value | *P* value |
| --- | --- | --- | --- |
| Clinical vs GPTV10 | Training | 0.741 | 0.4584 |
|  | Internal validation | 1.340 | 0.1803 |
|  | External validation | 1.157 | 0.2472 |
| Clinical vs Combined | Training | 2.480 | 0.0131 |
|  | Internal validation | 2.068 | 0.0386 |
|  | External validation | 2.388 | 0.0169 |
| GPTV10 vs Combined | Training | 1.689 | 0.0912 |
|  | Internal validation | 0.051 | 0.9598 |
|  | External validation | 0.325 | 0.7451 |

**Supplementary Figures**

**
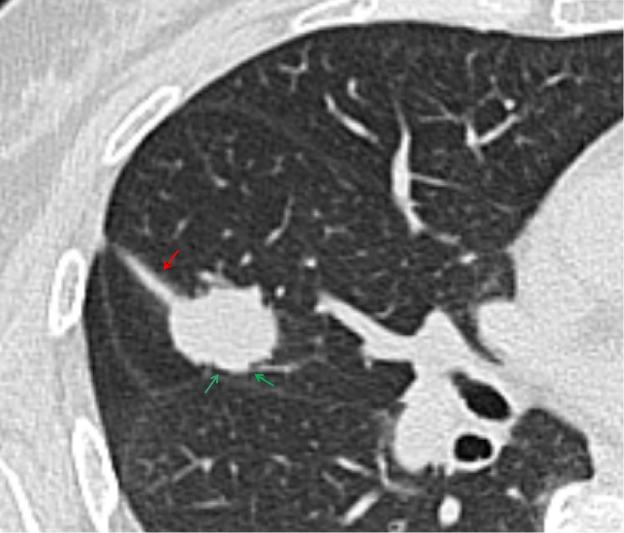

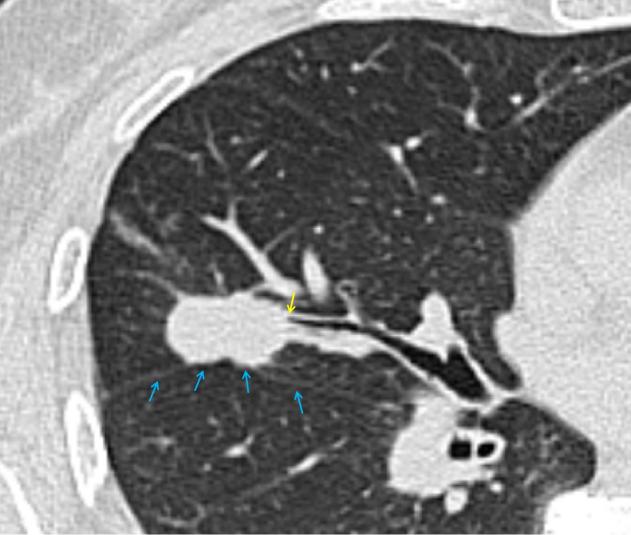
**

**
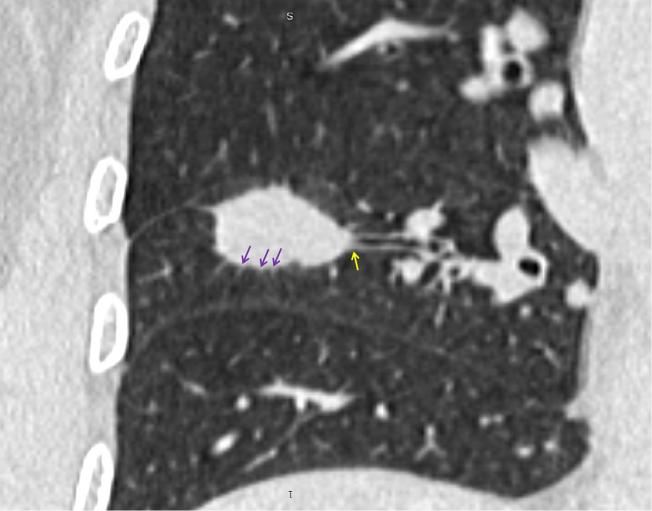

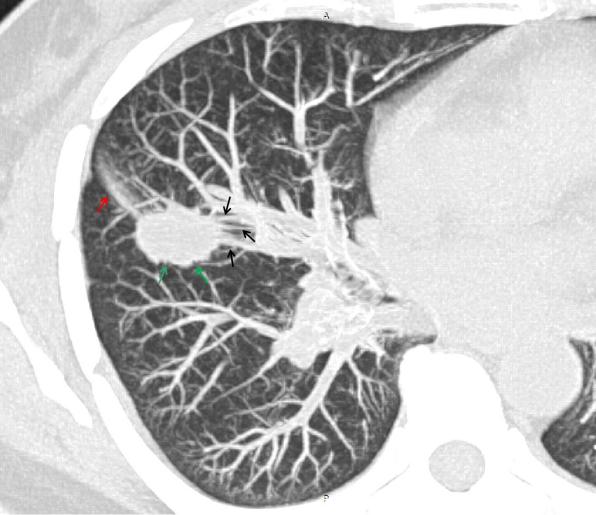
**

**Fig S1 to Fig S4** show a same patient, a 53-year-old female patient with lung adenocarcinoma and positive STAS status. The axial non-contrast computed tomography shows a solid nodule in the right middle lobe of the lung, CTR ≥ 50%, with distal ribbon sign (Figure S1, red arrow), lobulation sigh (Figure S1, green arrow), interlobar pleura indentation sigh (Figure S2, blue arrow), bronchial change (Figure S2 and S3, yellow arrow), spiculation sigh (Figure S3, purple arrow). The maximal intensity projection (Figure S4) shows a vascular convergence sign (black arrow), lobulation sigh (green arrow) and distal ribbon sigh (red arrow).


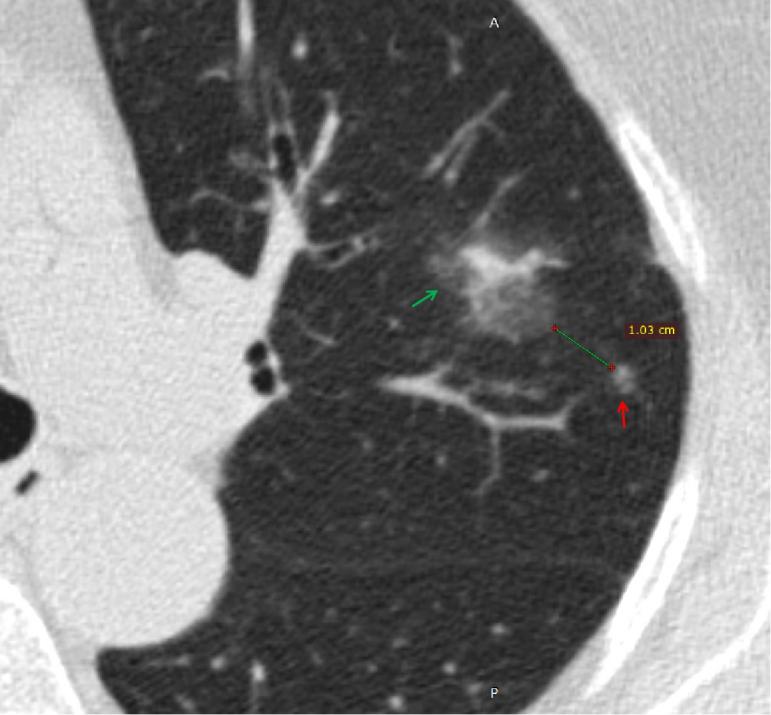


**Fig S5** shows a 77-year-old female patient with lung adenocarcinoma and negative STAS status. The axial non-contrast computed tomography shows a mixed ground glass nodule in the left upper lobe of the lung (green arrow) with satellite lesion sign (red arrow), the distance between the nodule and the satellite lesion is 1.03cm.


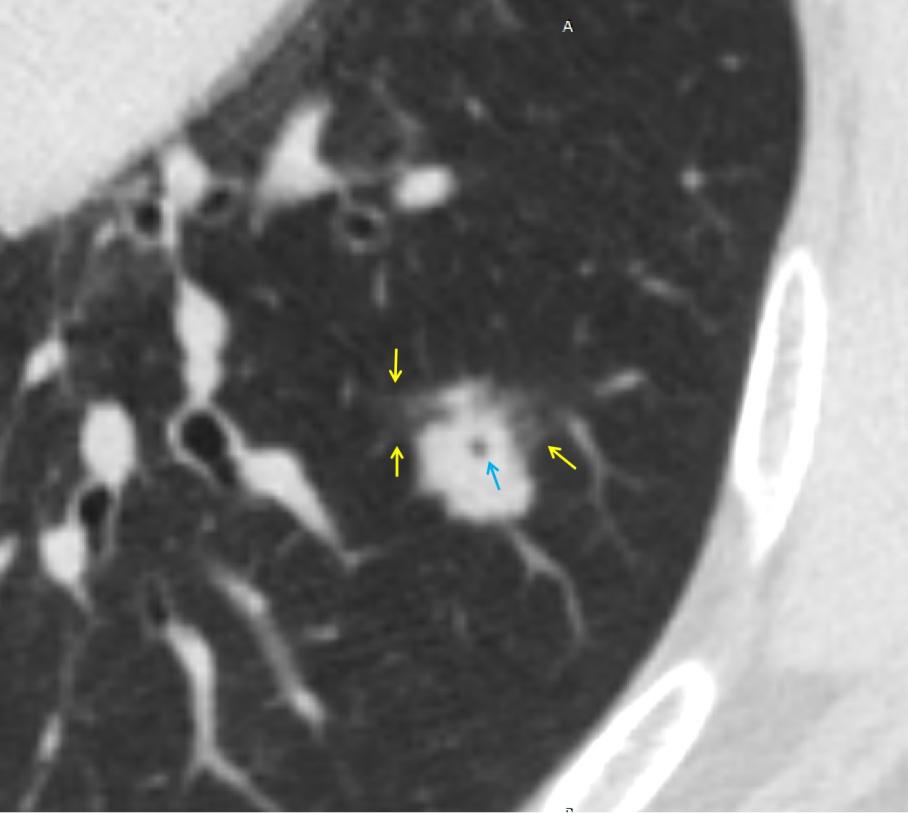


**Fig S6** shows a 74-year-old male patient with invasive mucinous adenocarcinoma and negative STAS status. The axial non-contrast computed tomography shows a solid nodule in the left lower lobe of the lung with halo sign (yellow arrow) and vacuole sign (blue arrow).

**
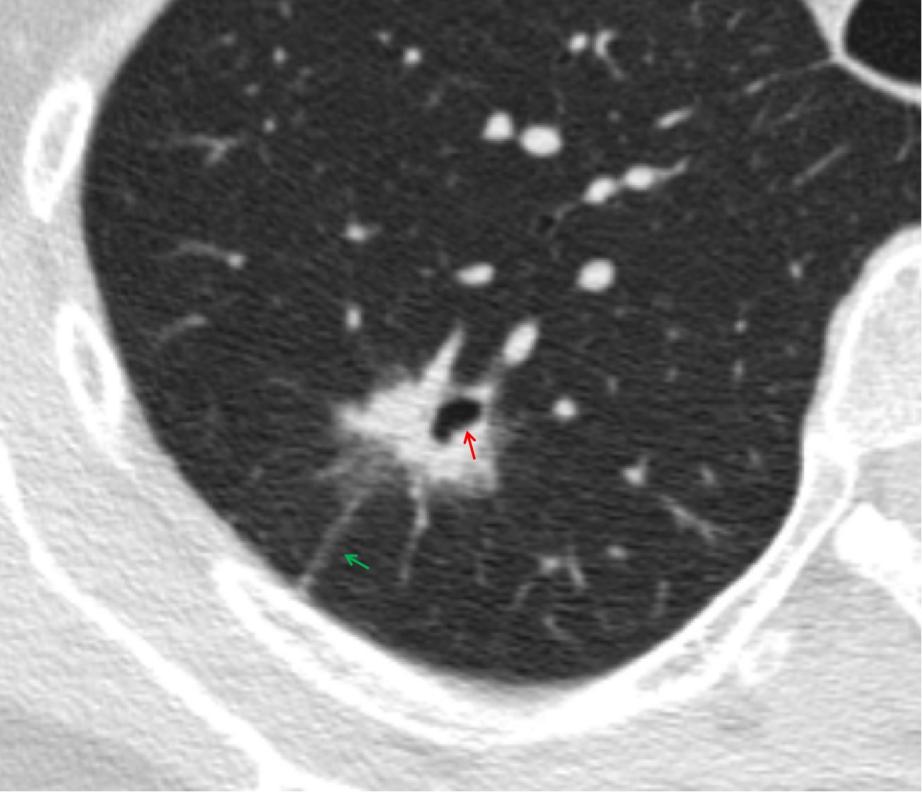
**

**Fig S7** shows a 62-year-old female patient with invasive adenocarcinoma and negative STAS status. The axial non-contrast computed tomography shows a mixed ground glass nodule in the right upper lobe of the lung with well-defined interface, pleural tags sigh (green arrow) and cavity or cystic airspace (red arrow).

**Fig S8** LASSO regression model selects the optimal feature subset to construct the radiomics model.

**Fig A** shows the cross-validation curve, and 10-fold cross-validation is used to select the optimal regularization parameter λ value. **Fig B** is the regression coefficient of LASSO. Each color line represents the curve of the regression coefficient of the radiomics features with λ value. Under the optimal regularization parameter λ value, the features whose coefficients are not equal to 0 are taken as the optimal feature subset to construct the radiomics model.


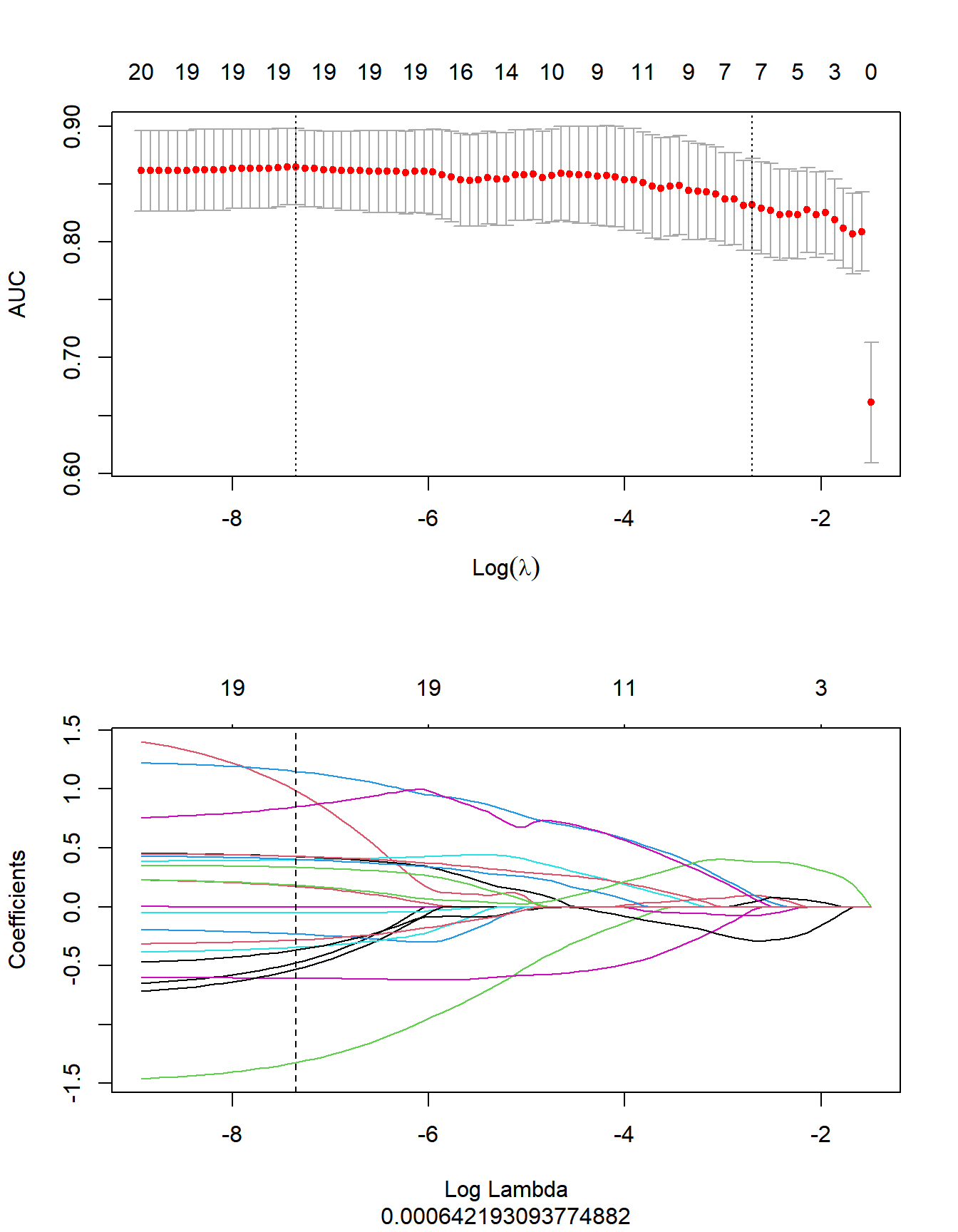

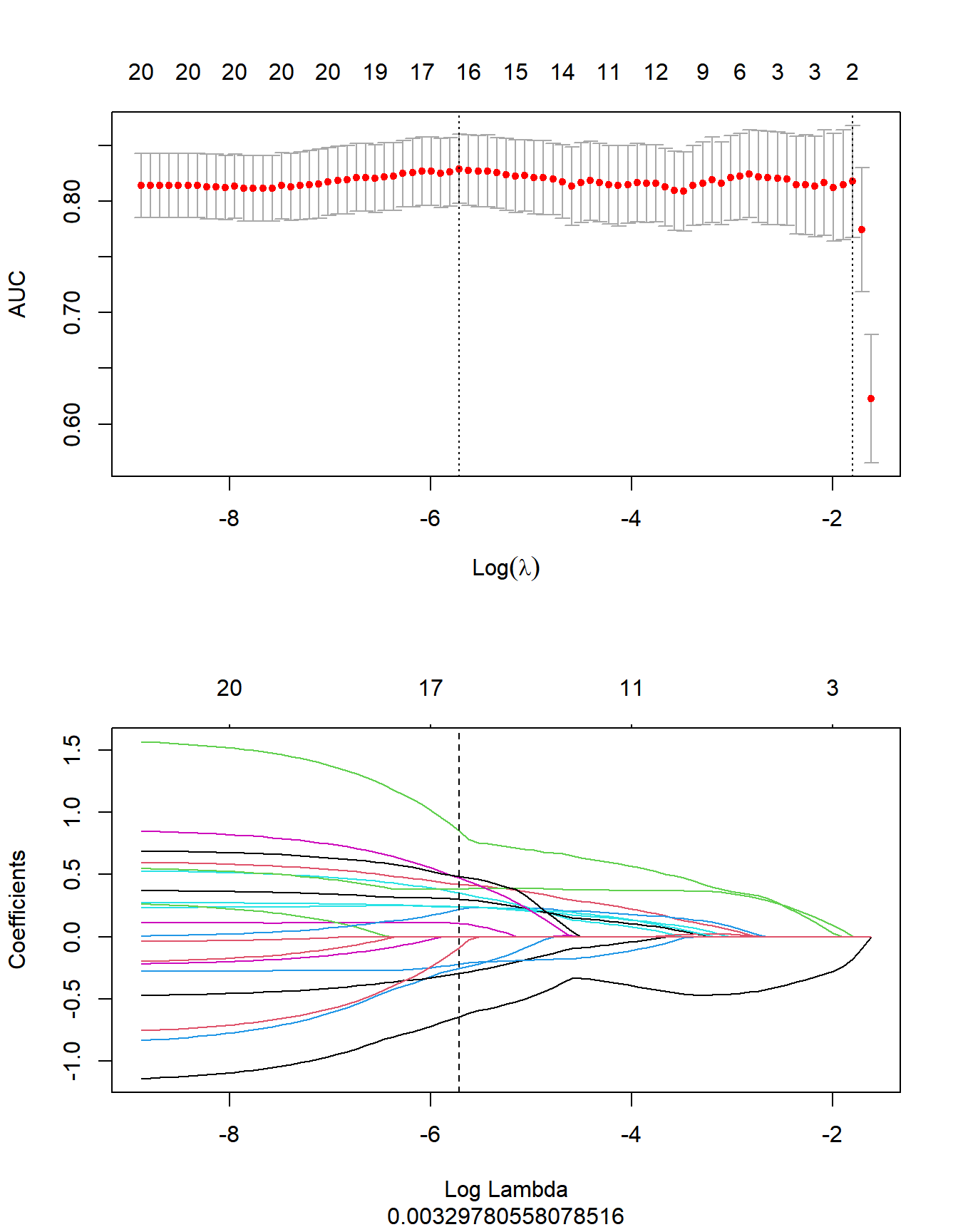


BA

AA

**GTV** **PTV5**


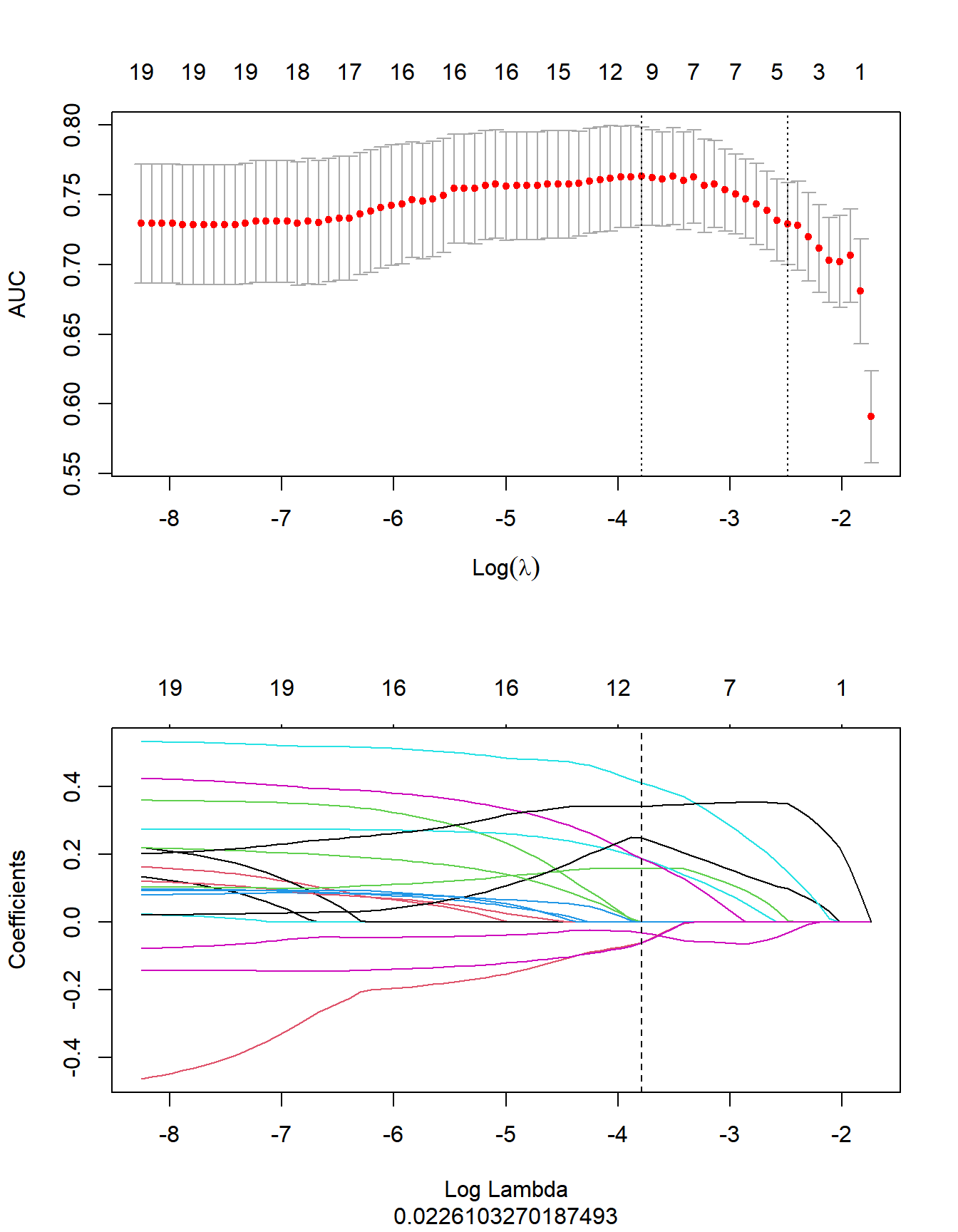

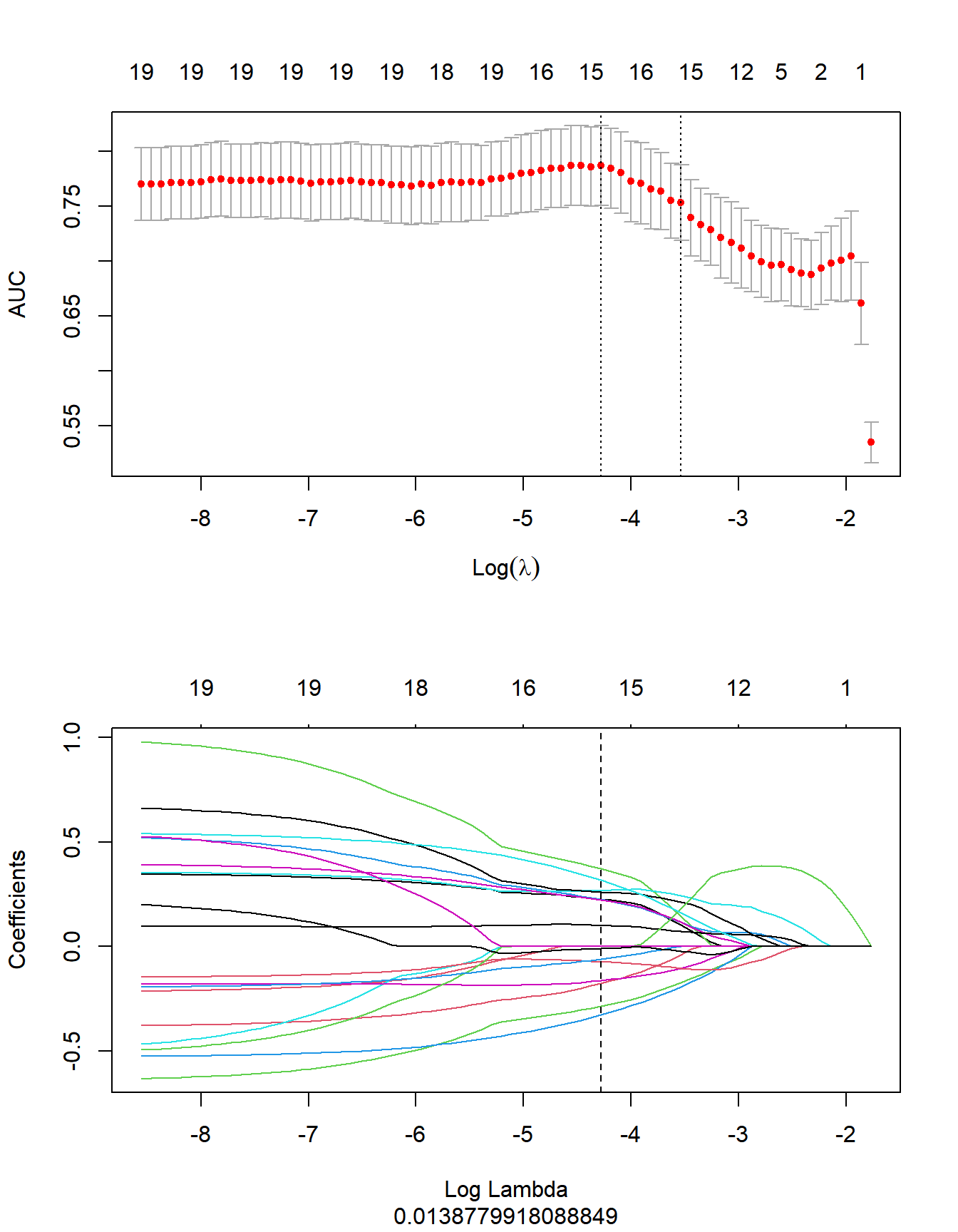


**PTV10** **PTV15**


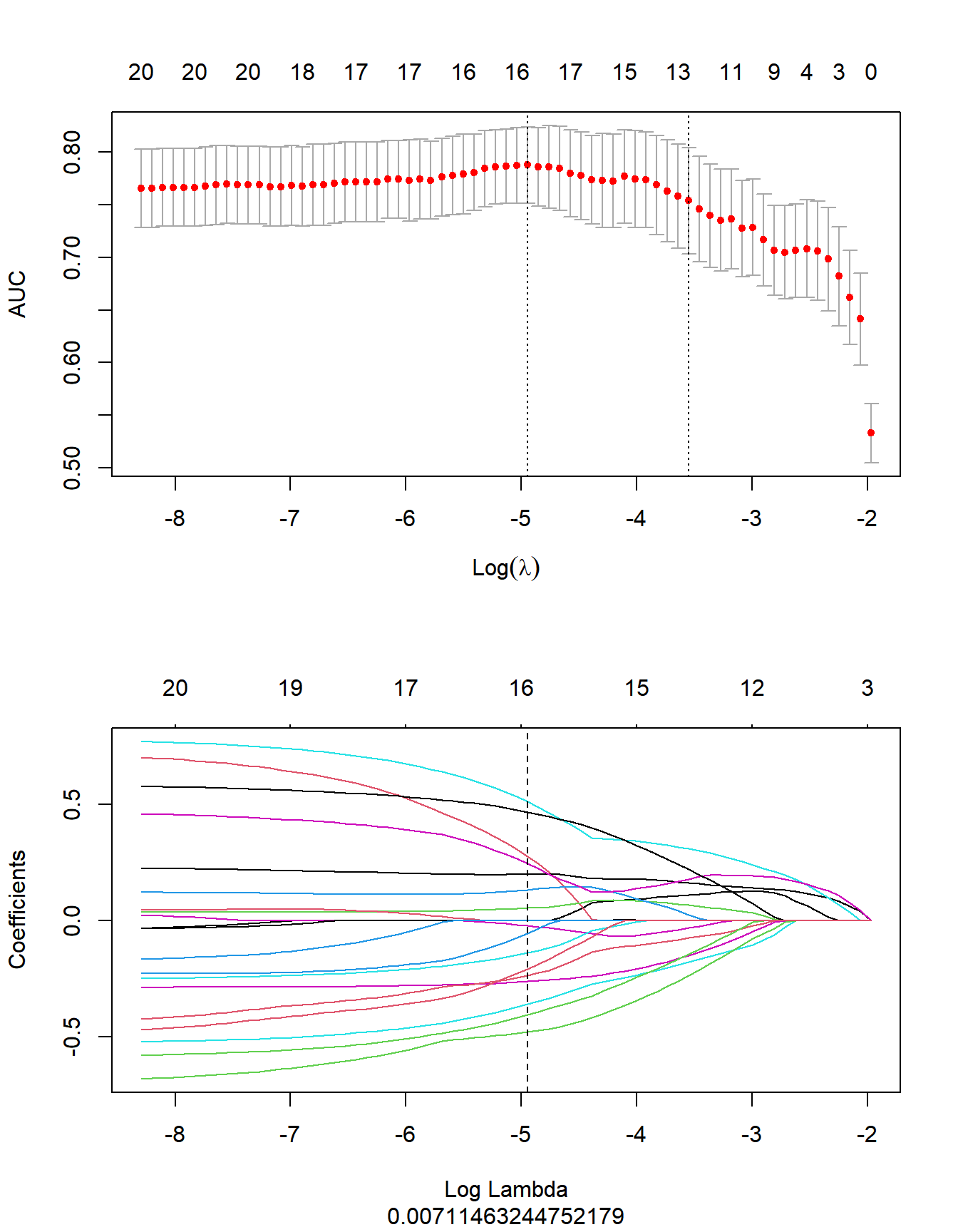

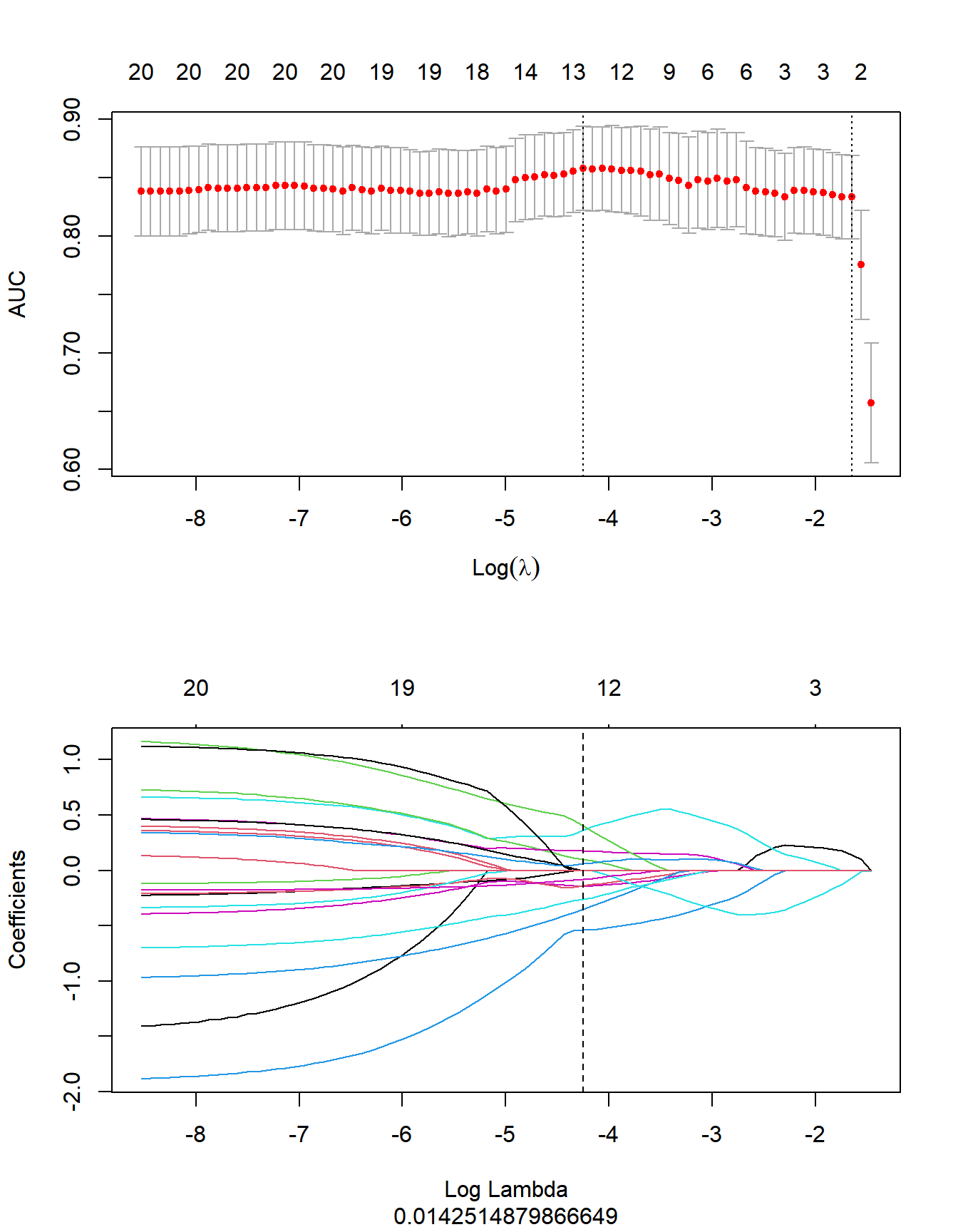


**PTV20** **GPTV5**


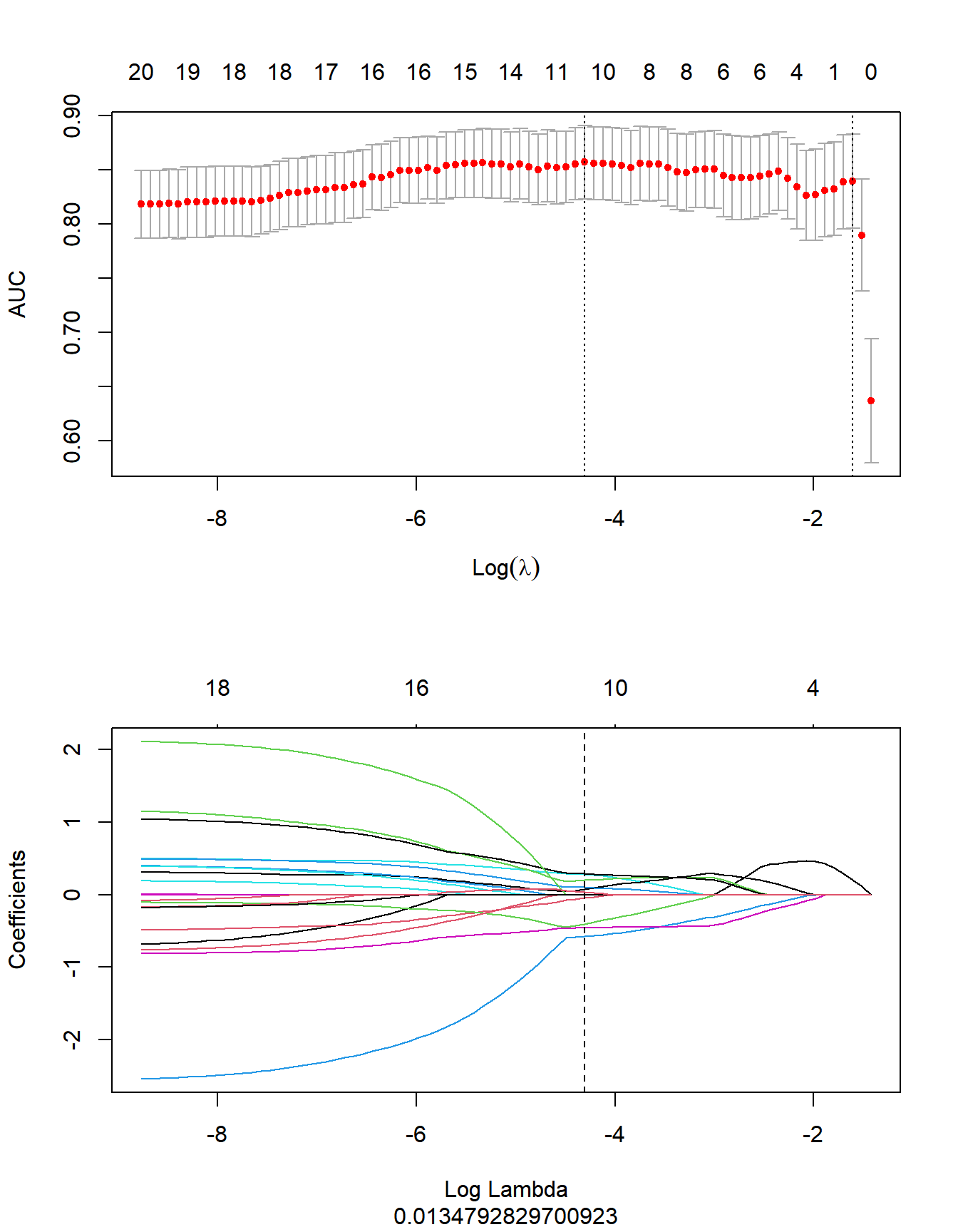

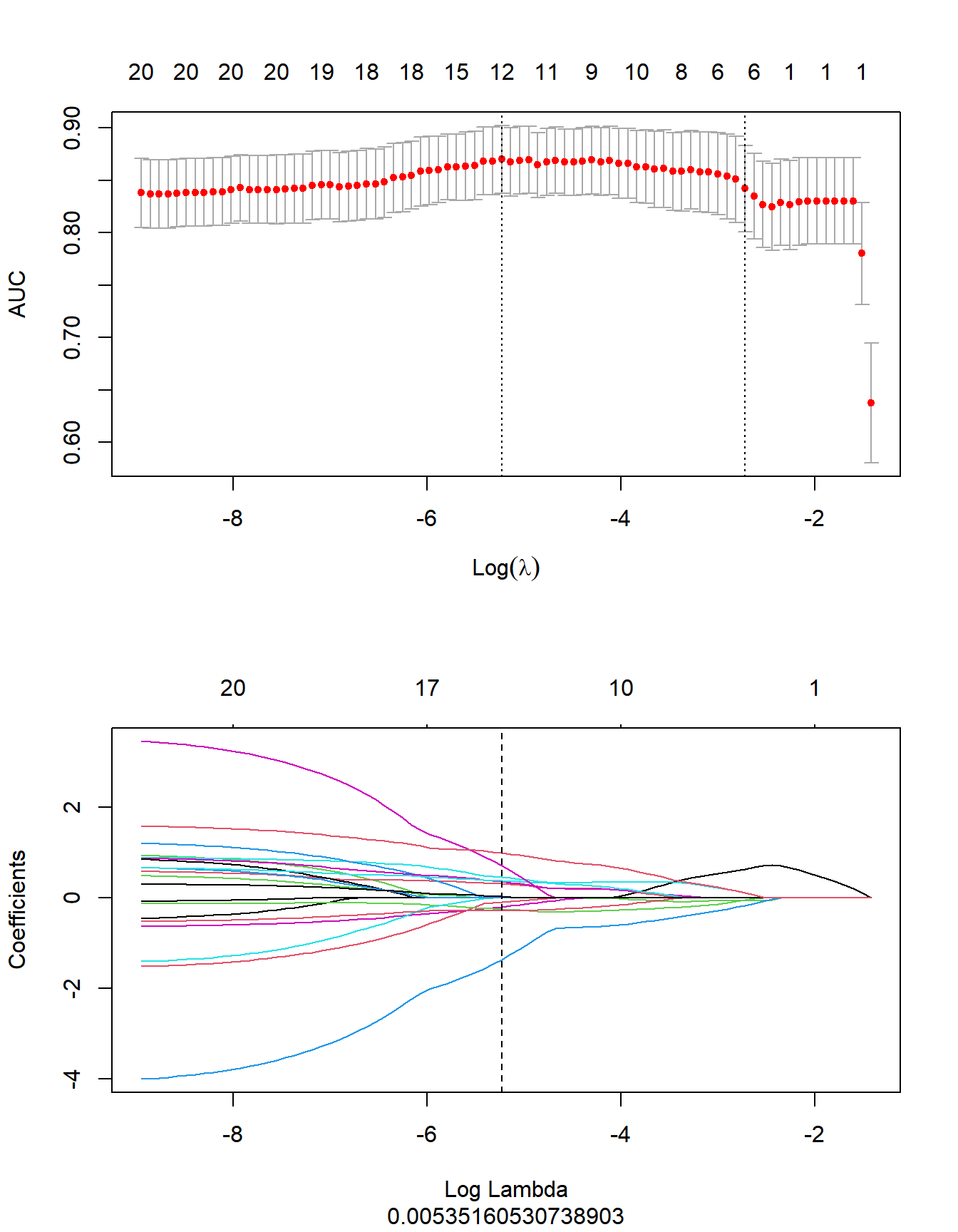


**GPTV10**  **GPTV15**


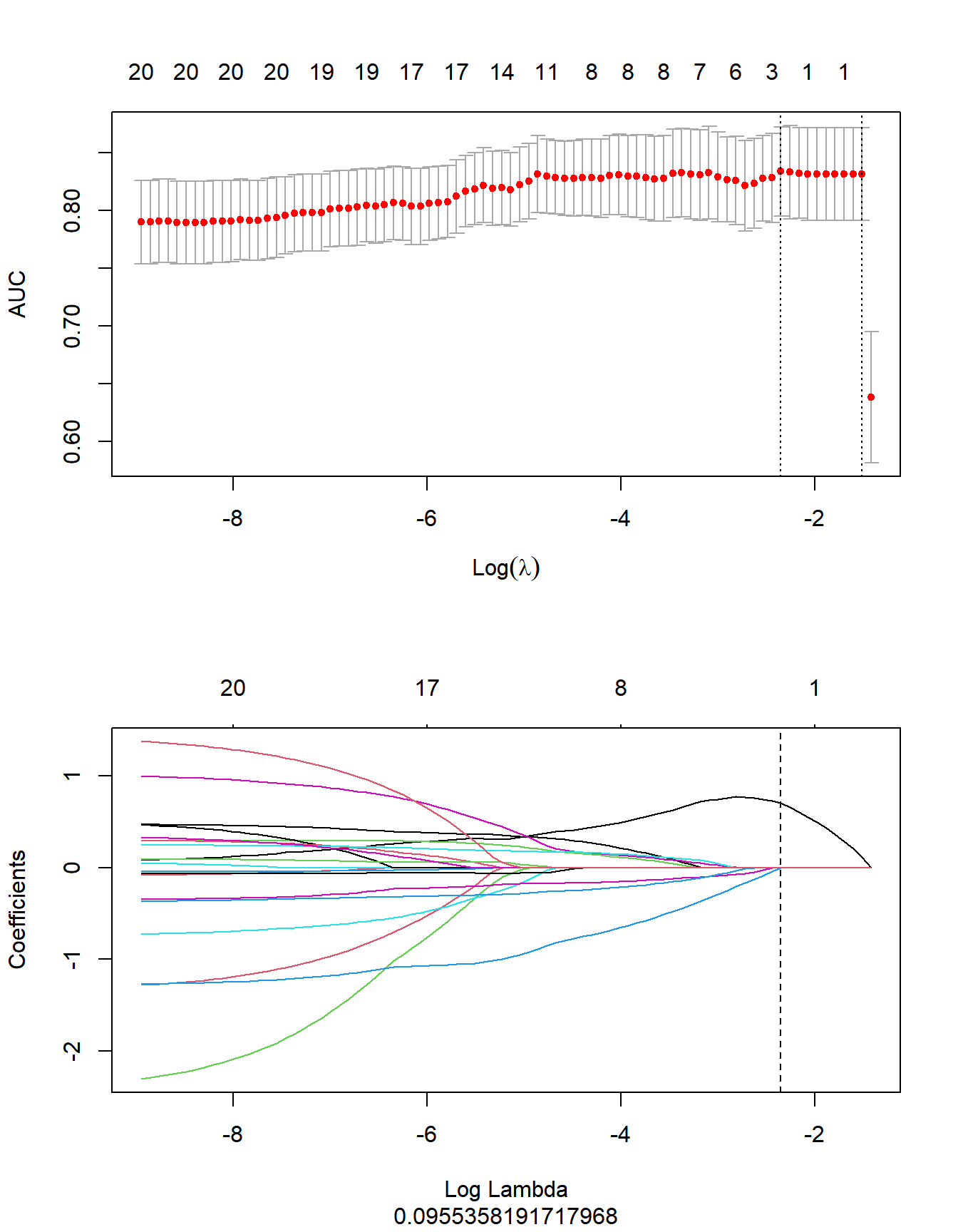


**GPTV20**

**Fig S9** The histogram of optimal radiomics feature subset and corresponding regression coefficient.The ordinate is the optimal radiomics feature subset selected after LASSO regression dimension reduction, and the abscissa is its corresponding regression coefficient.


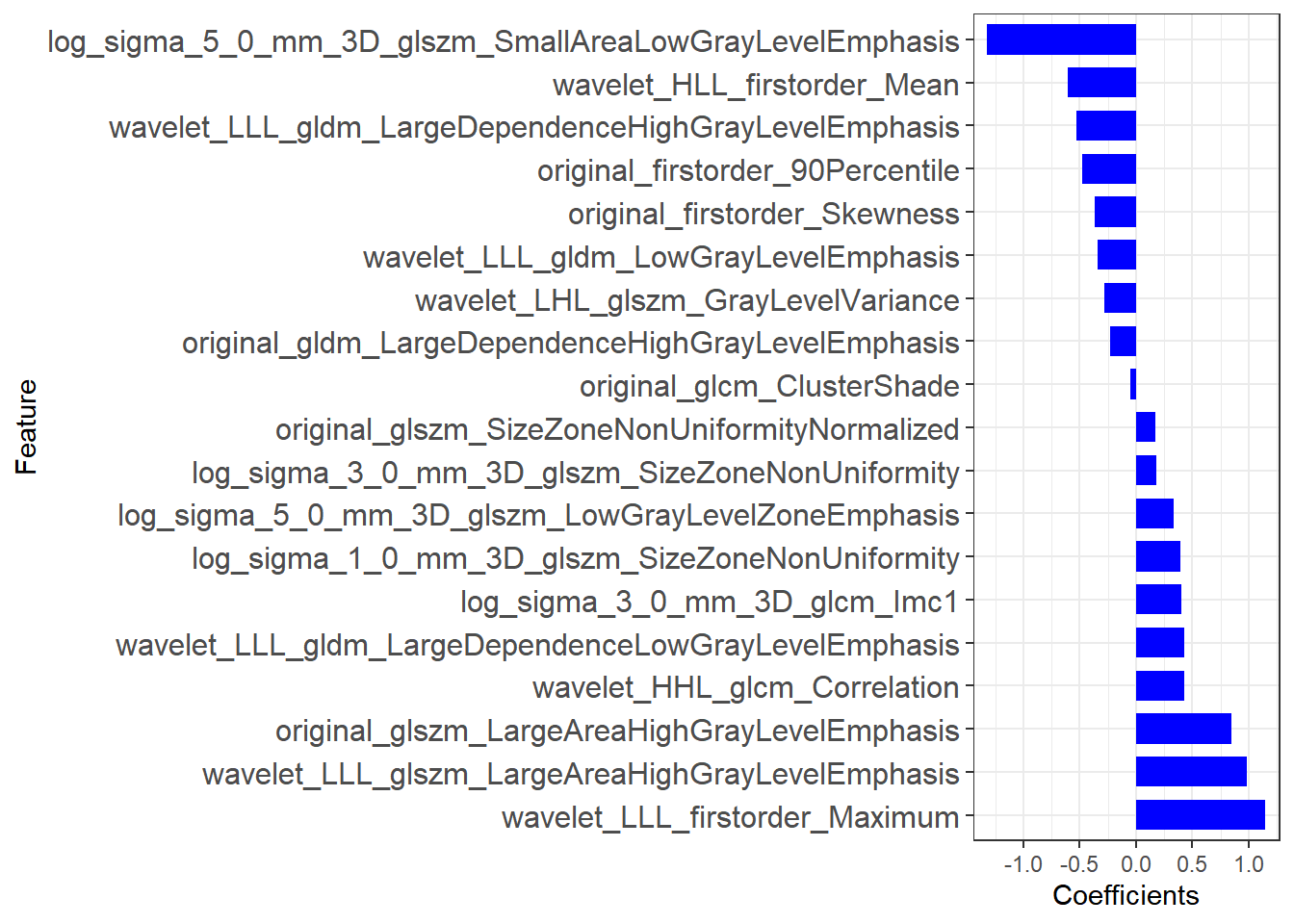

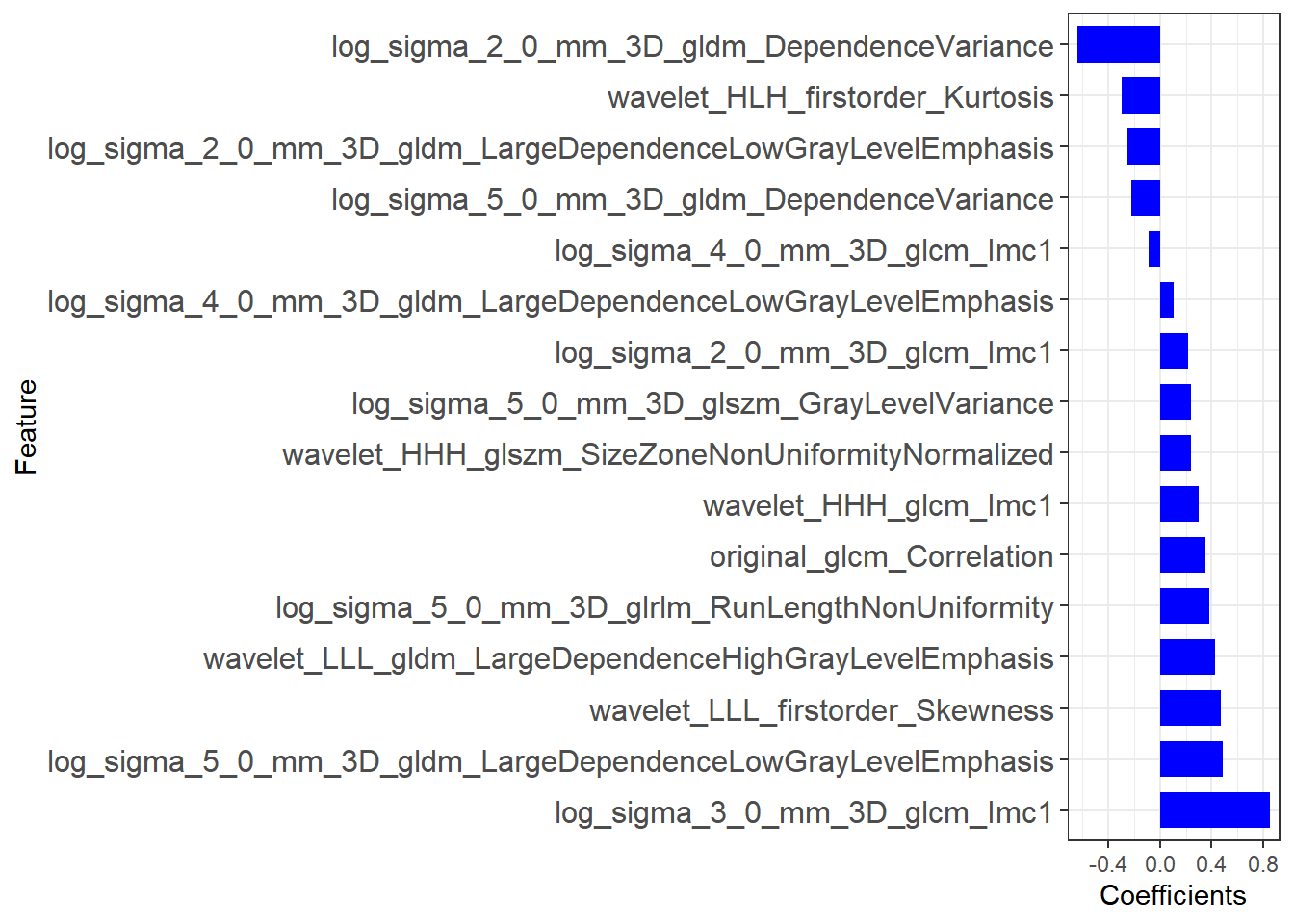


**GTV** **PTV5**


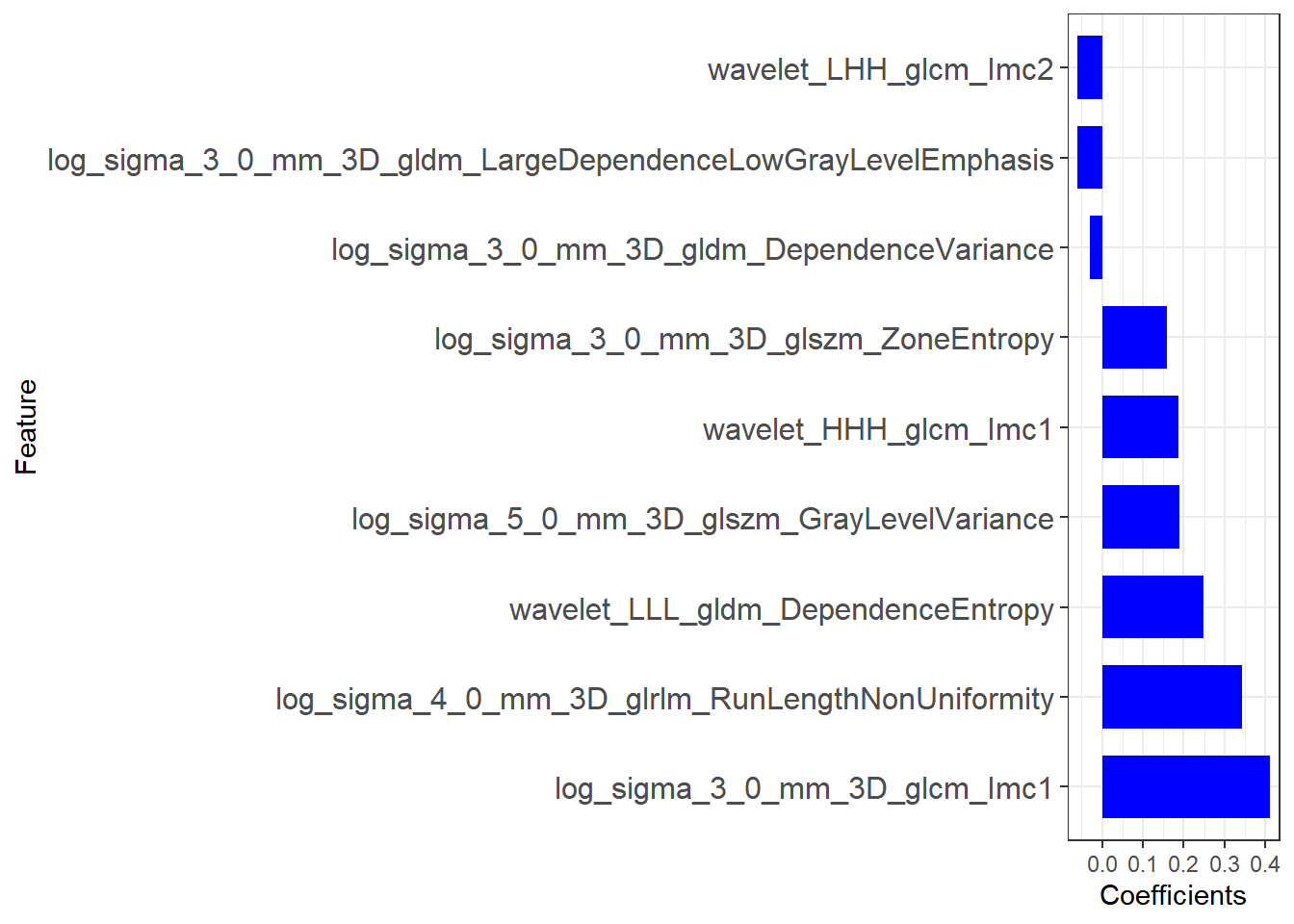

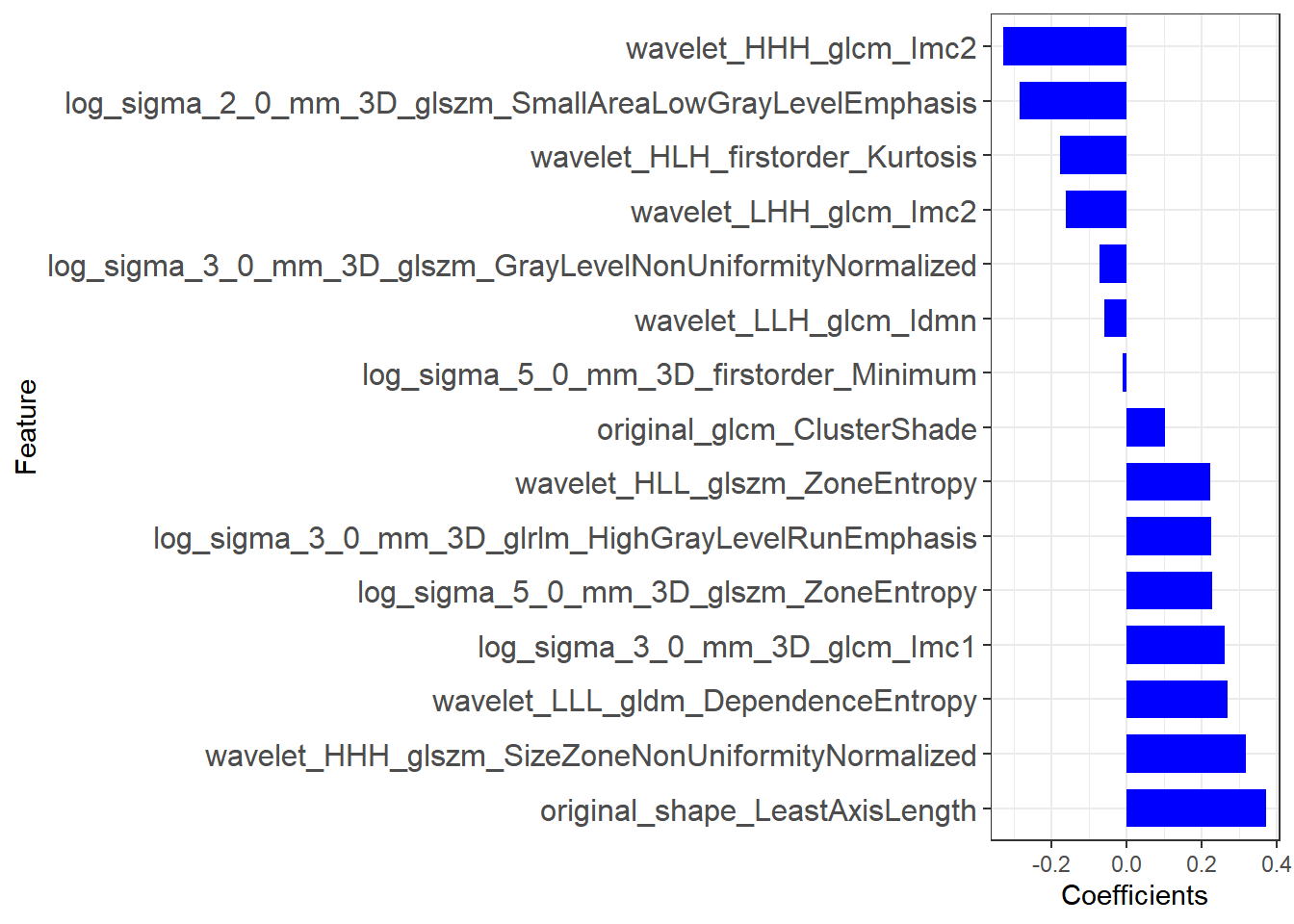


**PTV10**  **PTV15**


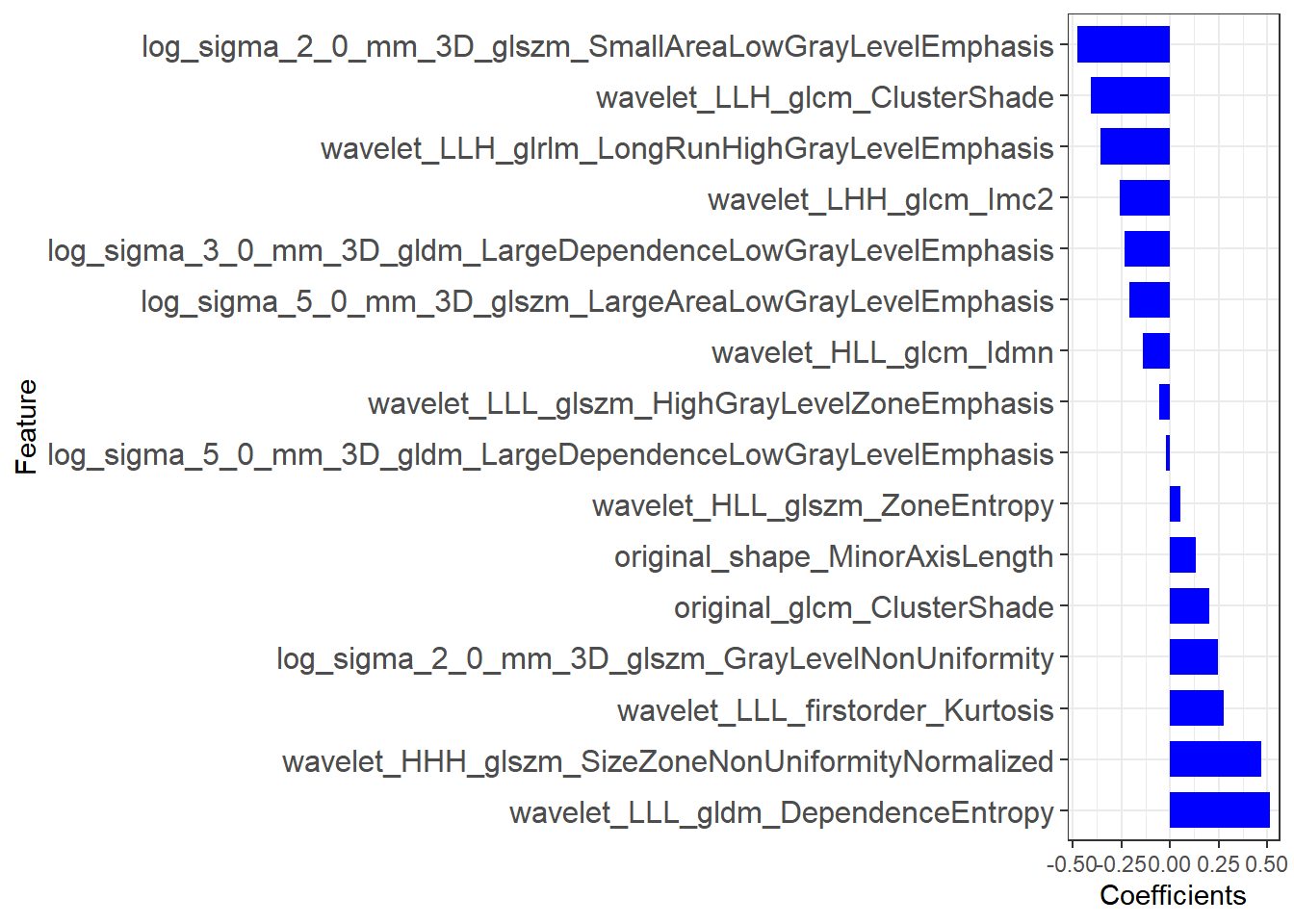

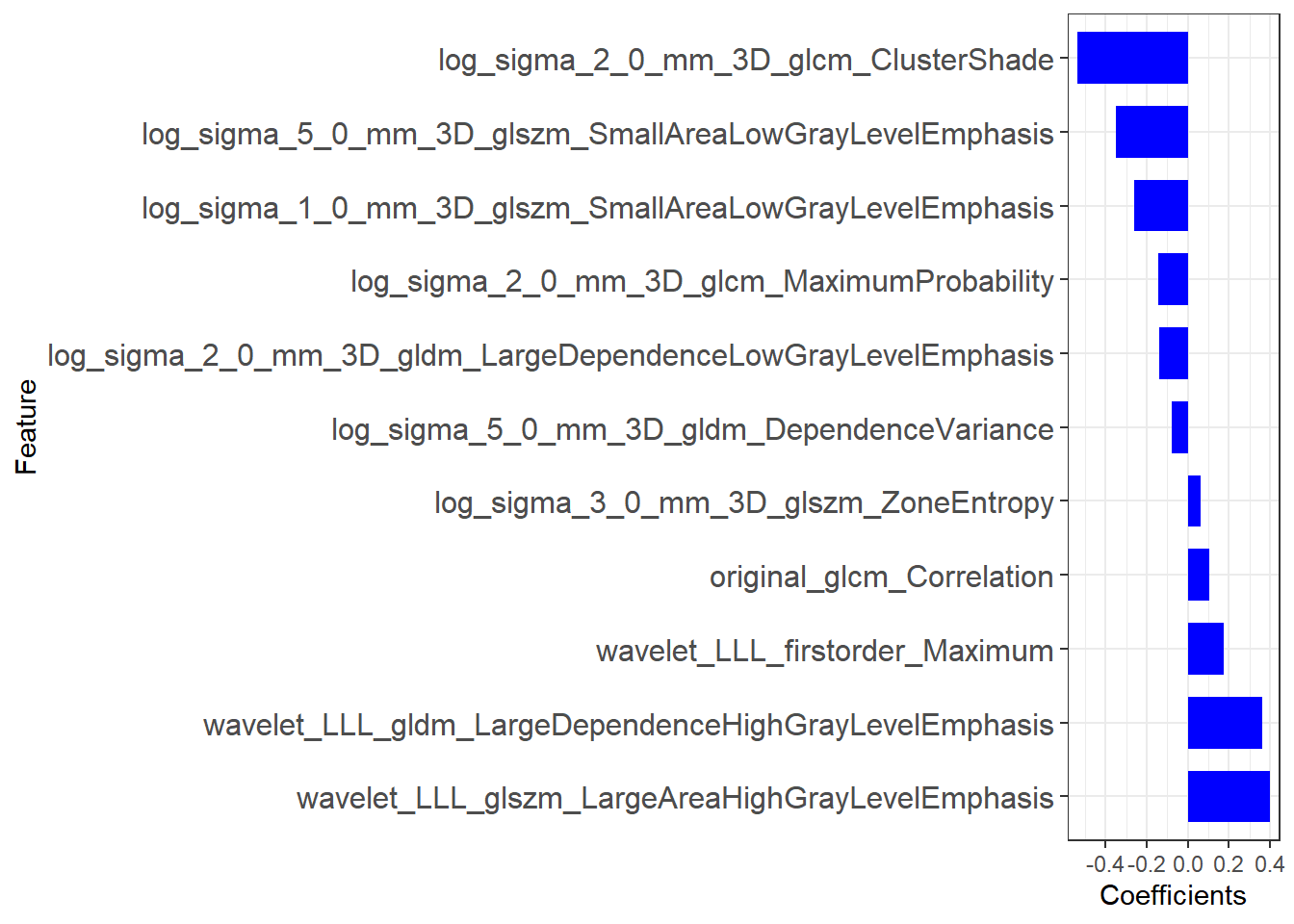


**PTV20** **GPTV5**


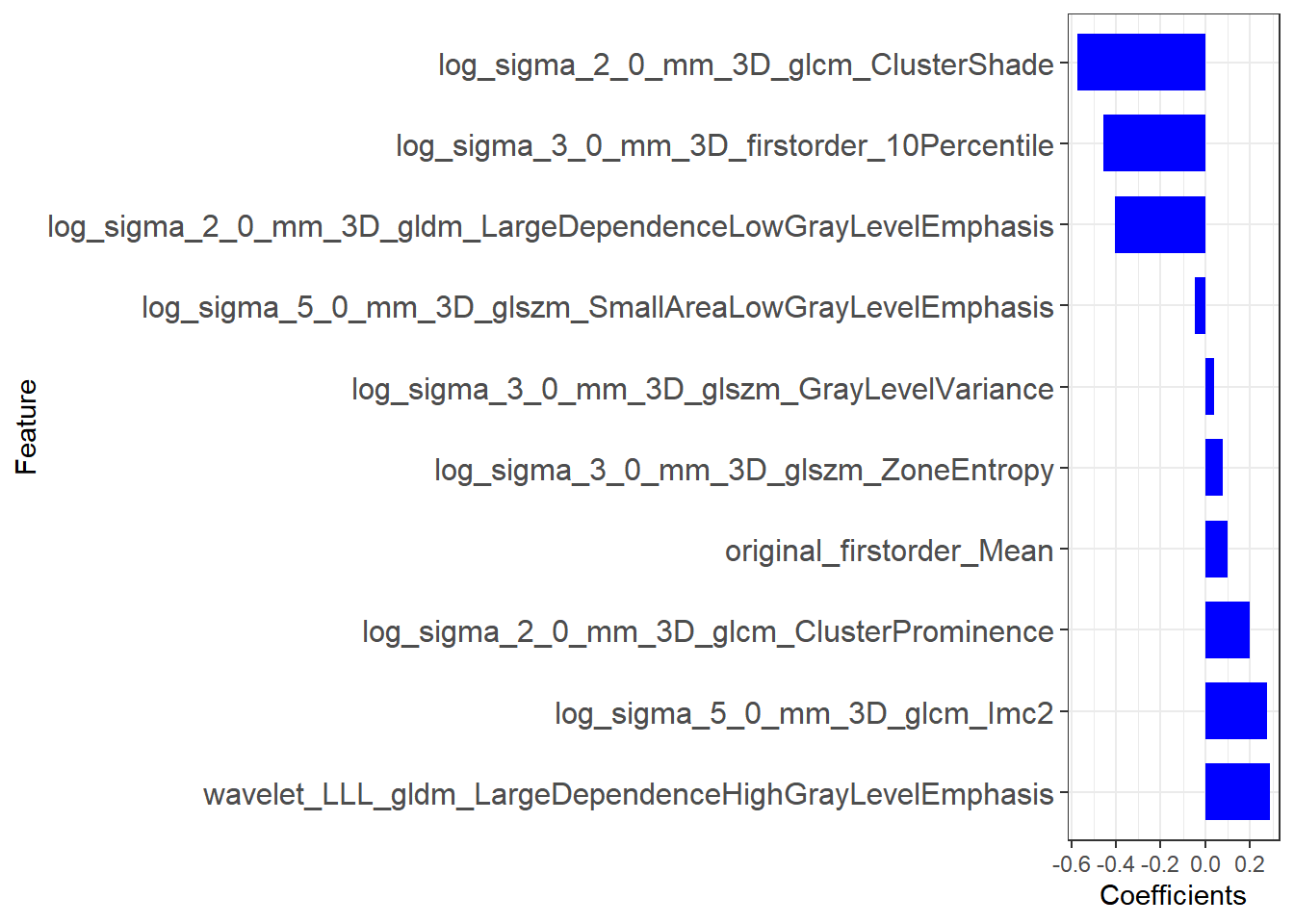

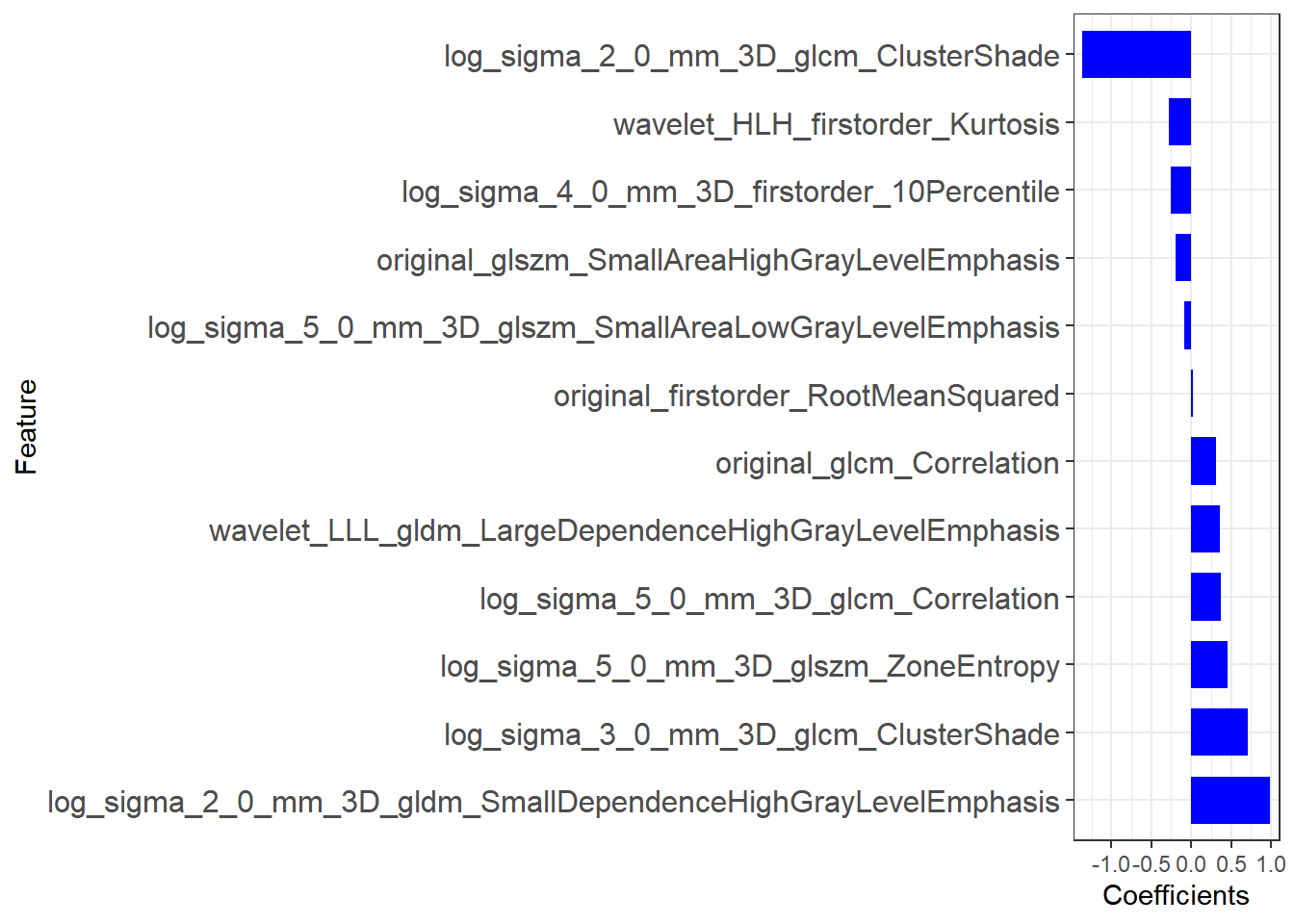


**GPTV10** **GPTV15**


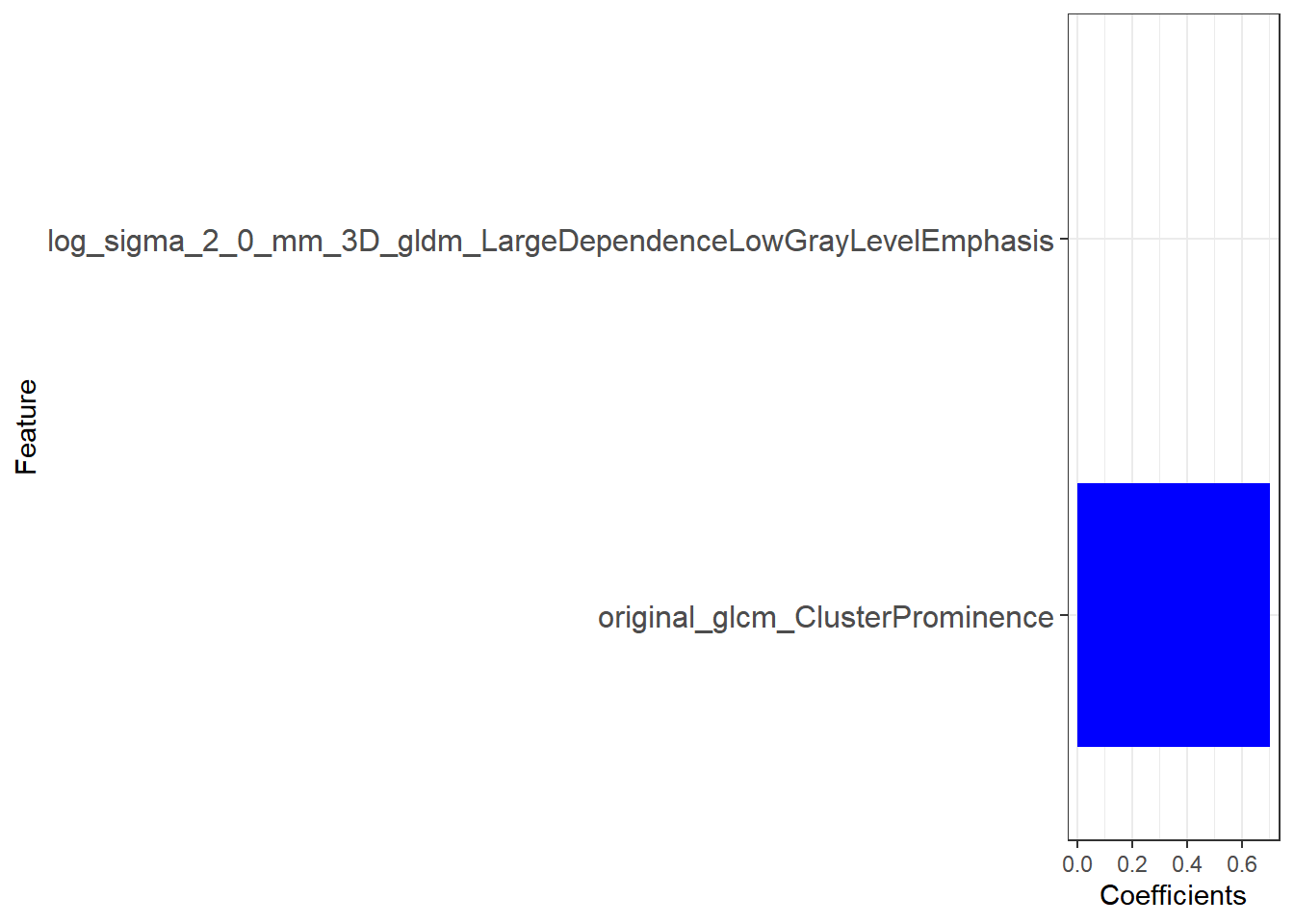


**GPTV20**

**Fig S10** Rad-score box scatter plot to predict the STAS status of clinical stage IA NSCLC.The abscissa Label 0 represents STAS negative, Label 1 represents STAS positive, and the ordinate represents Rad-score.


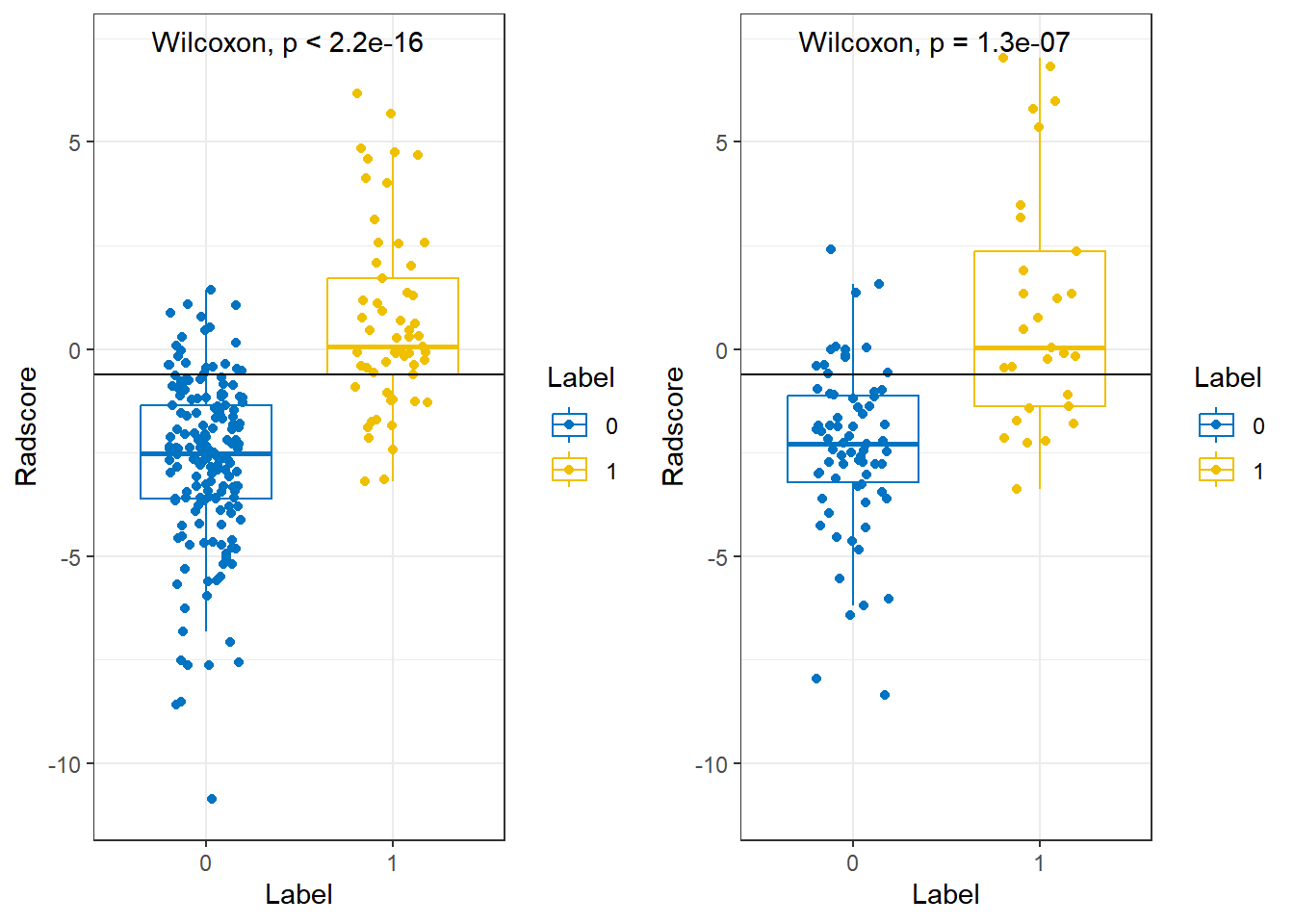

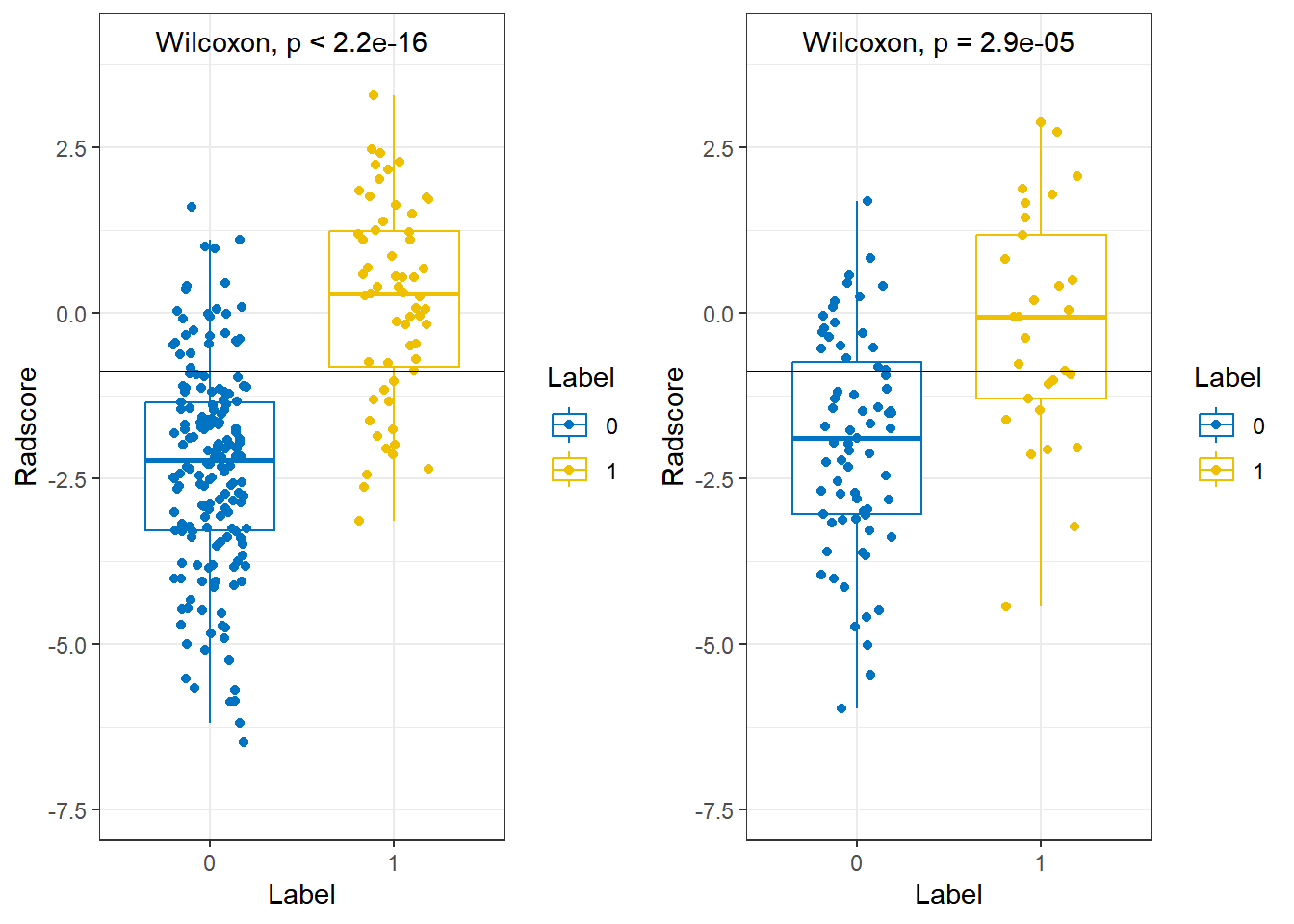


**GTV**  **PTV5**


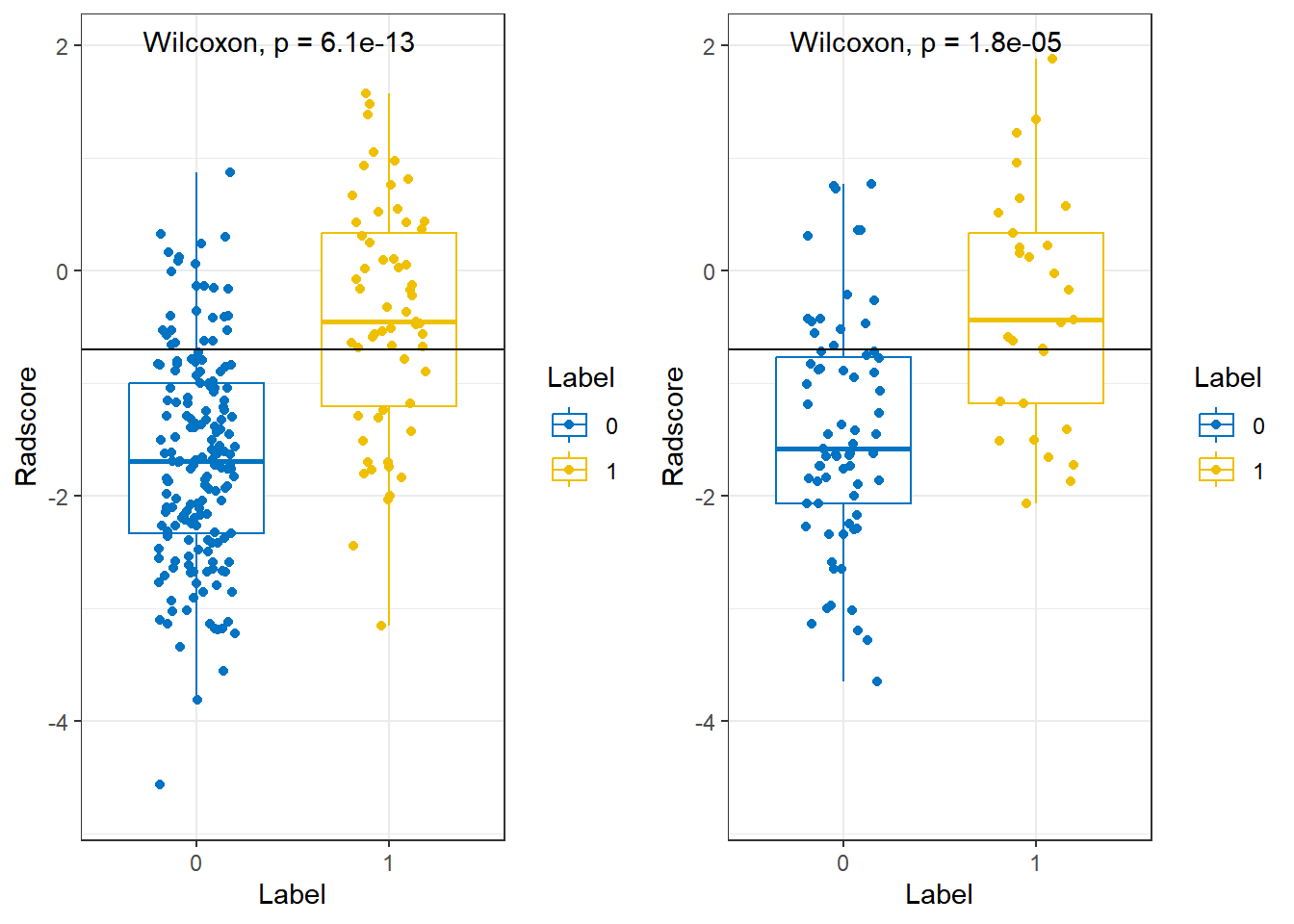

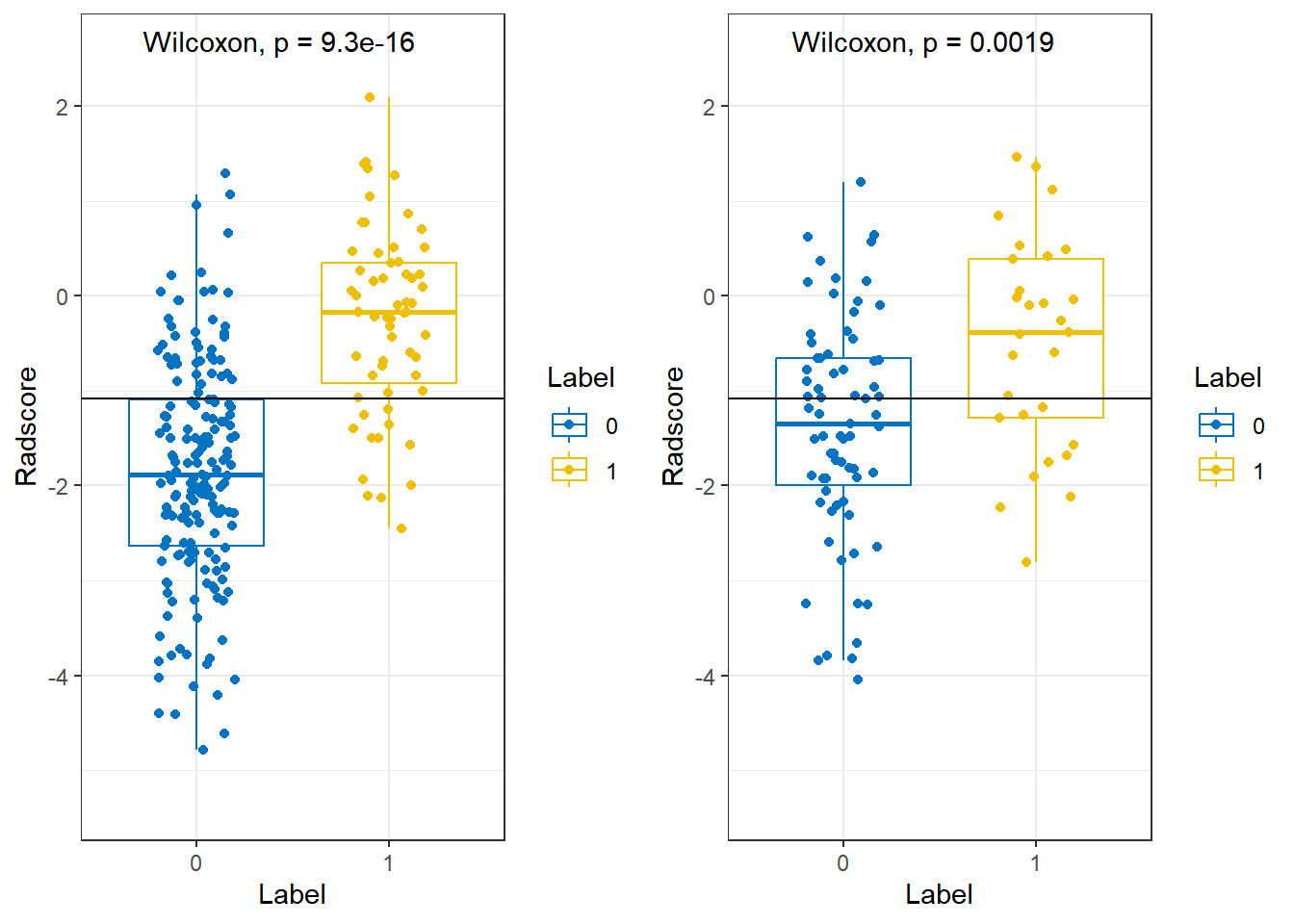


**PTV10**   **PTV15**


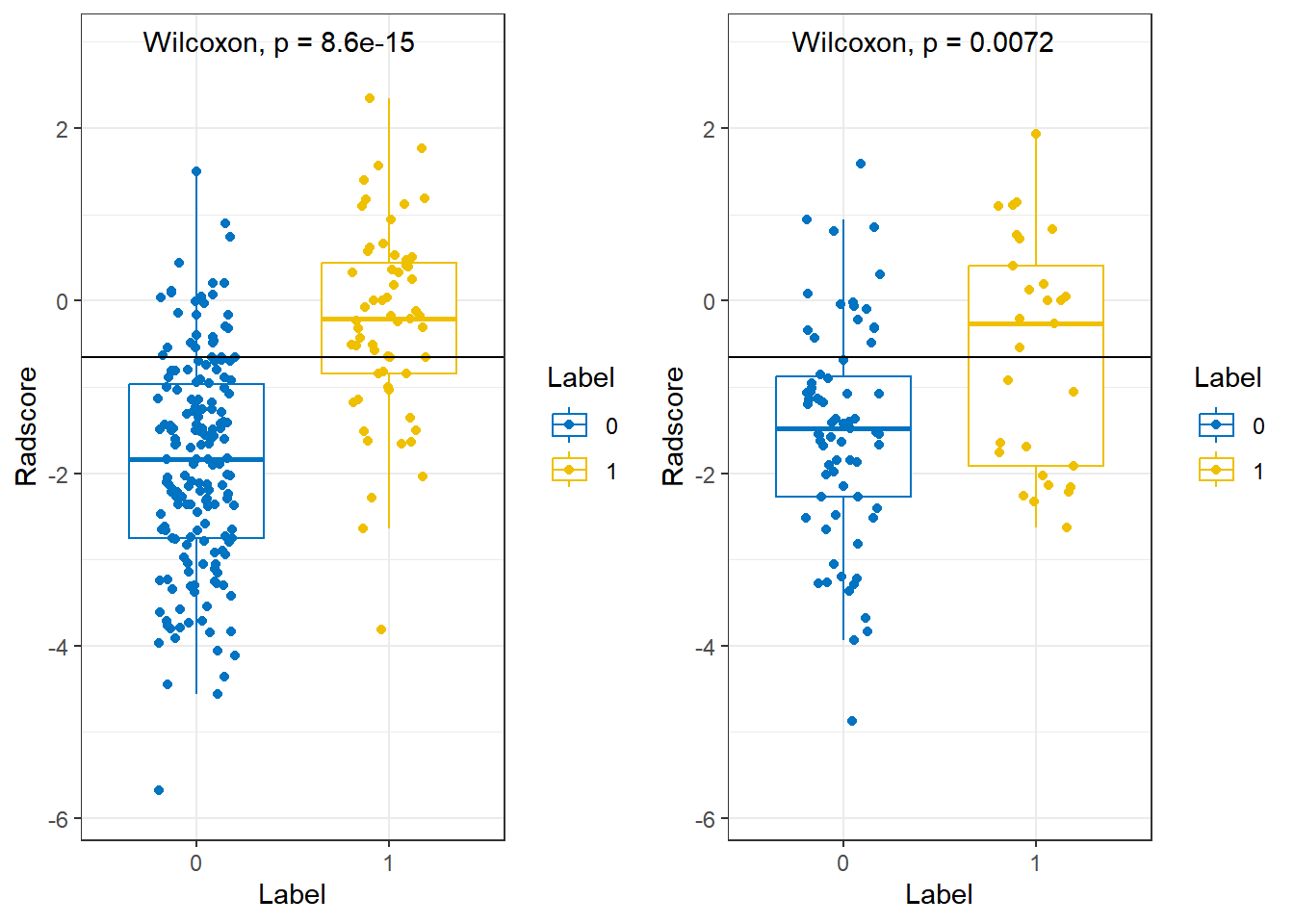

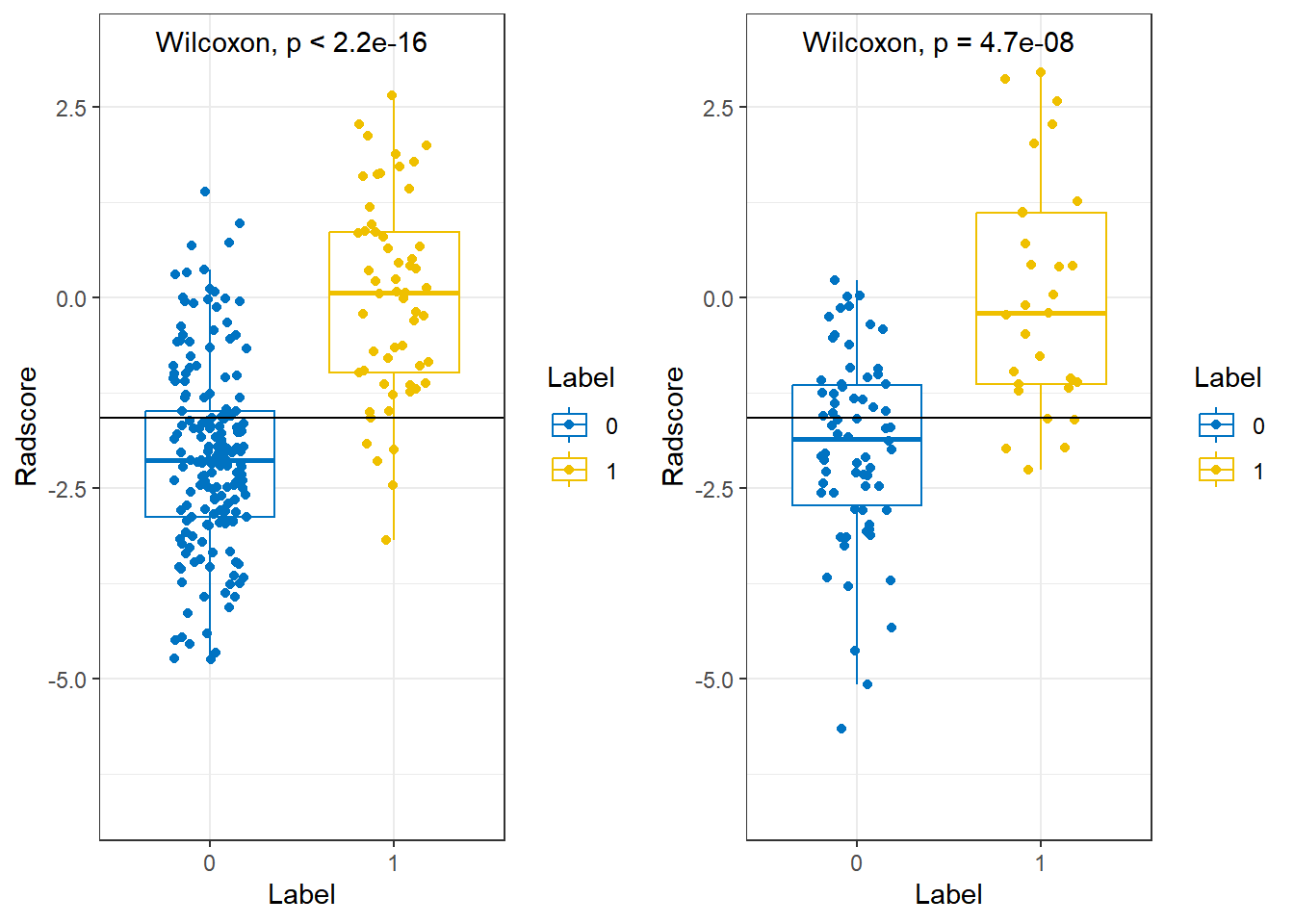


**PTV20**  **GPTV5**


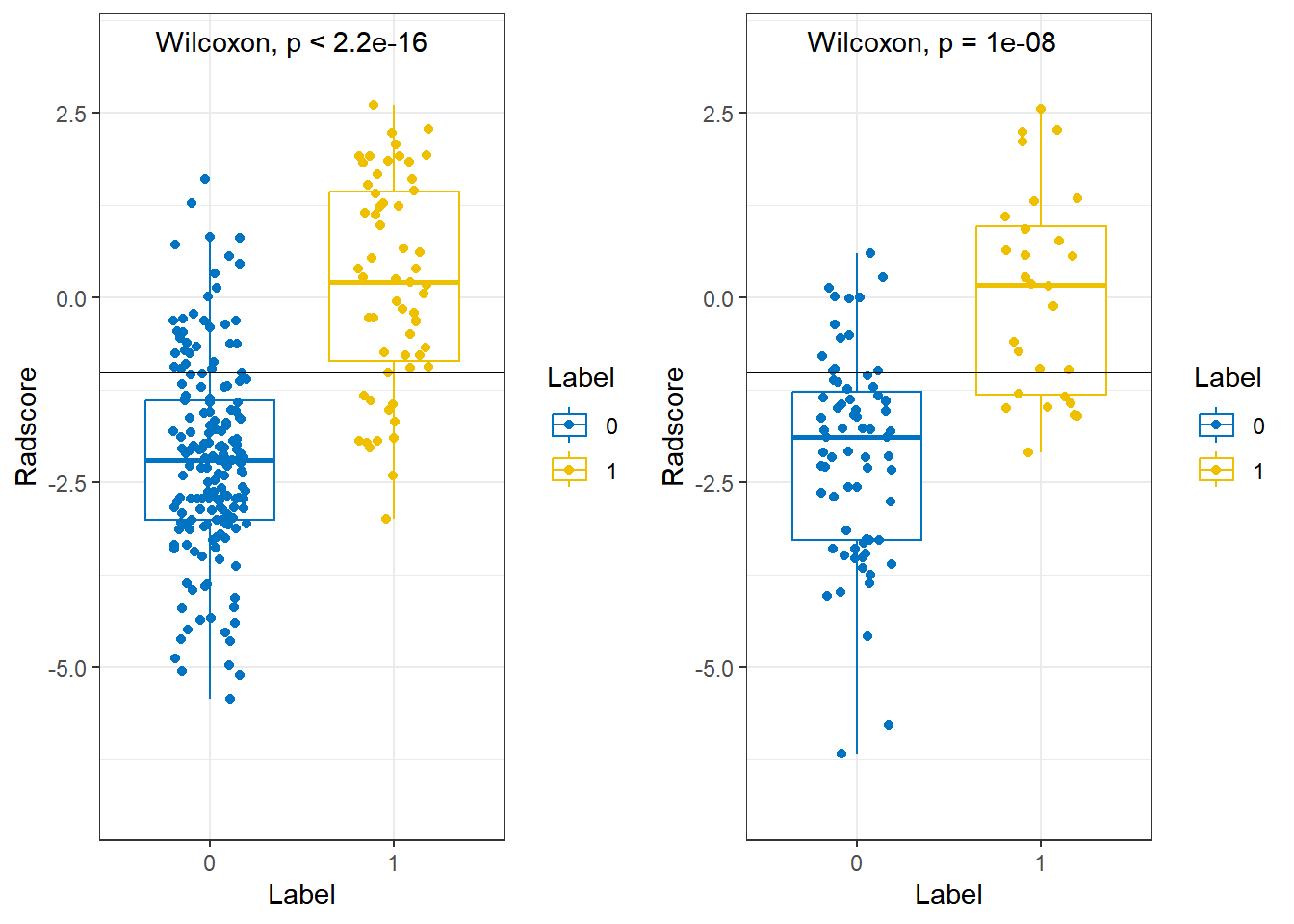

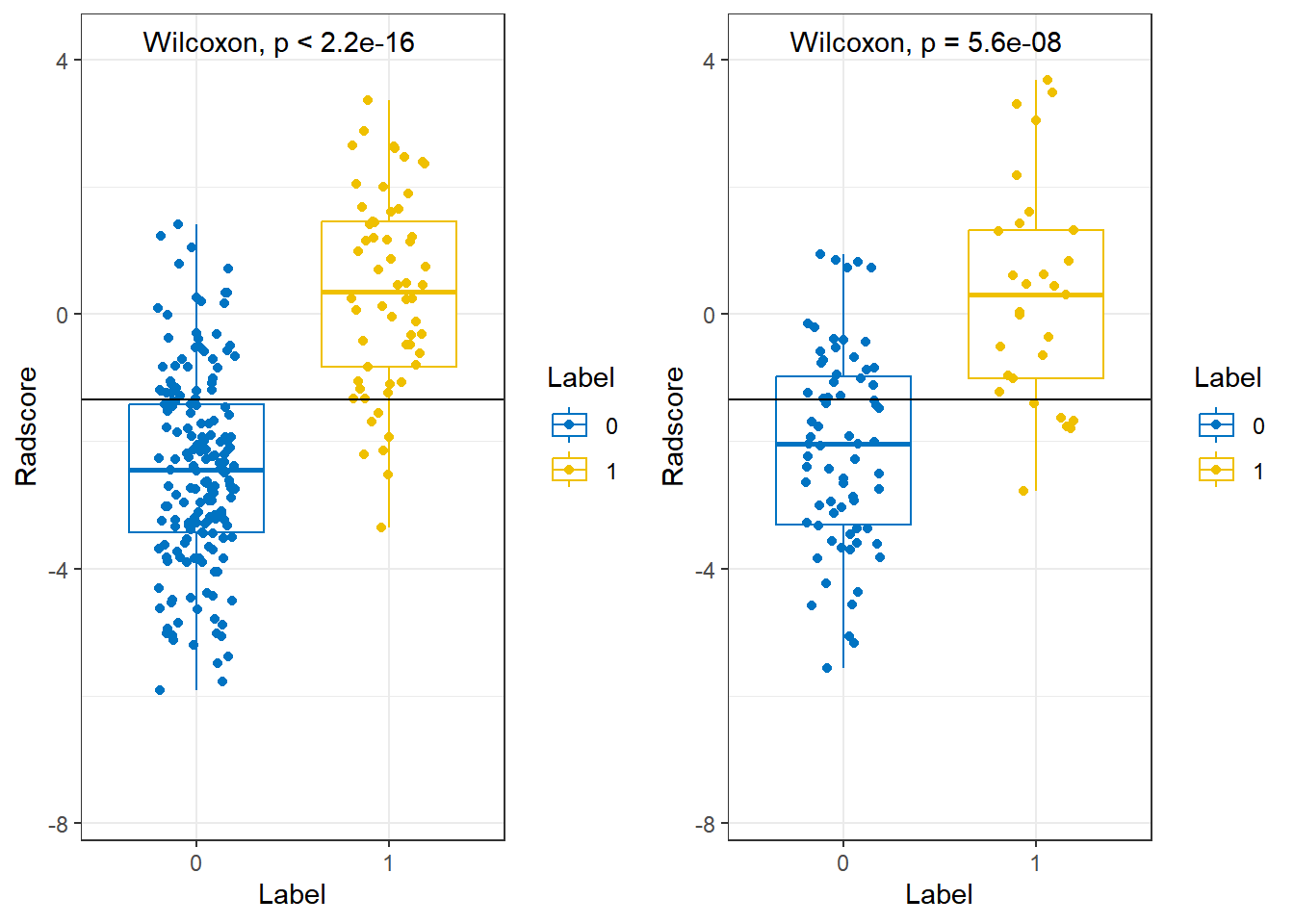


**GPTV10**  **GPTV15**


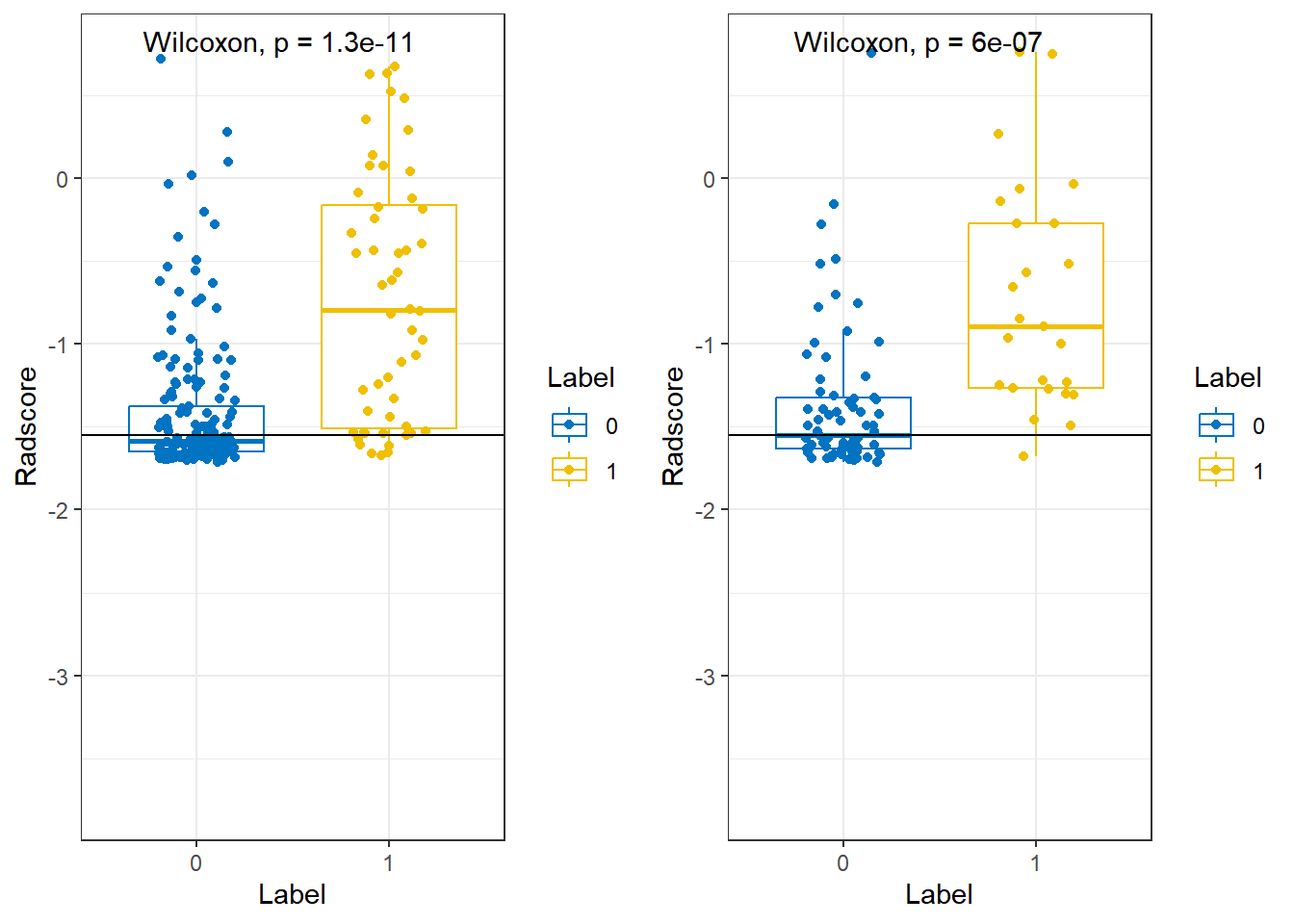


**GPTV20**
